# Co-evolution based machine-learning for predicting functional interactions between human genes

**DOI:** 10.1038/s41467-021-26792-w

**Published:** 2021-11-09

**Authors:** Doron Stupp, Elad Sharon, Idit Bloch, Marinka Zitnik, Or Zuk, Yuval Tabach

**Affiliations:** 1 Department of Developmental Biology and Cancer Research, The Institute for Medical Research Israel-Canada, The Hebrew University of Jerusalem, 9112001 Jerusalem, Israel; 2 Department of Biomedical Informatics, Harvard University, Boston, MA 02115 USA; 3 Department of Statistics and Data Science, The Hebrew University of Jerusalem, Jerusalem, 9190501 Israel

**Keywords:** Machine learning, Phylogeny, Protein function predictions, Phylogenetics

## Abstract

Over the next decade, more than a million eukaryotic species are expected to be fully sequenced. This has the potential to improve our understanding of genotype and phenotype crosstalk, gene function and interactions, and answer evolutionary questions. Here, we develop a machine-learning approach for utilizing phylogenetic profiles across 1154 eukaryotic species. This method integrates co-evolution across eukaryotic clades to predict functional interactions between human genes and the context for these interactions. We benchmark our approach showing a 14% performance increase (auROC) compared to previous methods. Using this approach, we predict functional annotations for less studied genes. We focus on DNA repair and verify that 9 of the top 50 predicted genes have been identified elsewhere, with others previously prioritized by high-throughput screens. Overall, our approach enables better annotation of function and functional interactions and facilitates the understanding of evolutionary processes underlying co-evolution. The manuscript is accompanied by a webserver available at: https://mlpp.cs.huji.ac.il.

## Abstract (web summary)

With the rise in number of eukaryotic species being fully sequenced, large scale phylogenetic profiling can give insights on gene function, Here, the authors describe a machine-learning approach that integrates co-evolution across eukaryotic clades to predict gene function and functional interactions among human genes.

## Introduction

The genomic revolution has resulted in the sequencing of thousands of species, with many more being sequenced every year. This explosion of genomic data from a diverse set of species can be readily analyzed using comparative genomics approaches to study the crosstalk between genes, function, traits, and the species harboring them. One such approach is phylogenetic profiling, an established method for identifying functionally related genes and protein–protein interactions (PPIs) in both prokaryotes and eukaryotes^1–10^. Phylogenetic profiling is based on the hypothesis that functionally related genes are associated with similar evolutionary pressures and thus were lost or retained together throughout evolution. For example, genes related to cilia were identified and classified by comparing the proteome of non-ciliated organisms to species with either a prototypical or modified cilia^4,11^. Similarly, others successfully identified mitochondrial genes based on their evolutionary pattern of loss and retention in different species^5,12^.

In recent years, following the ever-increasing number of sequenced organisms, it has become possible to apply co-evolutionary analysis at the clade level (e.g., animals, mammals, fungi), comparing signals at different evolutionary scales. It was hypothesized that functionally related genes might show different co-evolution patterns in specific clades. This phenomenon may stem from genes becoming functionally related later in evolution (e.g. the function was first introduced in the last common ancestor of some clade). Other more interesting co-evolutionary processes may also be found when inspecting co-evolution at the clade level. For example, a group of genes functionally interacting in a common ancestor may lose their co-evolution in some but not all subclades further down the tree.

With the aim to better capture co-evolution, “clade-wise” phylogenetic profiling approaches were developed^3,7,10^. Shin and Lee showed how integrating phylogenetic profiles across domains of life improved the prediction of functionally interacting genes. Later, Sherill-Rofe and Rahat^3^ identified DNA repair-related genes by integrating seven clade-wise co-evolution signals, revealing the applicability of such methods. However, these approaches either had a low resolution—focusing on domains of life—or were applicable only to prediction on gene sets. It was recently demonstrated that clade-wise co-evolution can improve functional interaction prediction between human genes at the eukaryotic clade level^10^. Furthermore, the performance of classic phylogenetic profiling has been shown to saturate when more species are added^13^. In contrast, clade-wise phylogenetic profiling has the potential to improve performance when more species are sequenced.

It was previously hypothesized that different types of pathways (e.g. metabolic, signaling) might co-evolve in different manners. Some biological processes may be more amenable to change than others, leading to a variety of co-evolutionary phenomena. For example, signaling pathways may rewire more often throughout evolution than metabolic pathways, thus generating different patterns in the phylogenetic profiles for different pathway types^14^. This represents how the interaction context, in this case pathway type, can be inferred directly from co-evolutionary patterns. Accordingly, clade-wise signals can potentially be used to boost the prediction of functionally related genes as well as the prediction of the interaction context.

One particular application of phylogenetic profiling is in assigning function to less studied genes. Many human genes are largely uncharacterized and are thus referred to as the “Ignorome”. Pandey et al.^15^ studied the brain Ignorome and found that approximately 70% of studies address the top 5% most studied genes, with 20% of genes barely mentioned in the literature. Others have found similar patterns in other Ignoromes, focusing on why some genes are highly researched while others are generally overlooked. These works identified the time of first description to be the most prominent factor (i.e. a rich get richer phenomena)^16,17^. More recently, the NeXtprot consortium identified ~2000 human genes with no known function^18^. Phylogenetic profiling presents an unbiased approach to annotation of gene function, allowing us to better understand the function of Ignorome genes.

Phylogenetic profiling entails evolutionary insights into how pathways evolved. Previous works identified evolutionary insights from phylogenetic profiling by linking organism traits with groups of co-evolved genes. This is based on the hypothesis that a coordinated loss of functionally related genes in specific organisms suggests that these organisms underwent a major phenotypic change. For example, loss of heme biogenesis genes in ticks and parasitic nematodes was associated with adaptation to an environmental source of heme^2^. Similarly, Li et al. analyzed mitochondrial genes with respect to known losses of the mitochondrial genome^5^ and Dey and others inspected cilia pathways in ciliated and non-ciliated organisms^4,5,11^. However, these findings were either driven by manual inspection^2,4^ or were limited by the complexity of the model^5,19^. At the macro level of pathway co-evolution, Dey et al.^14^ identified pathway types that were identifiable by phylogenetic profiling and characterized pathway types by divergence and evolutionary age, identifying a relationship between co-evolution and general function as stated above.

Here, we present a supervised machine-learning approach to phylogenetic profiling utilizing “clade-wise” co-evolution of functionally related genes. This approach predicts functional interactions between human genes and the interaction context (i.e. the biological function) in which the functional interaction takes place. We then extend this method to annotate genes function, focused on the functional annotation of less-studied genes. Based on the predictions for each pathway type, we prioritize function and interaction partners for these genes with specific examples of DNA repair candidate genes as validated by existing evidence in the literature. Finally, we inspect the evolutionary insights revealed by our method at the pathway level, pathway type, and the macro all pathways level. These evolutionary insights lead us to identify the importance of parasitic species in the predictions of our approach, and potentially other phylogenetic profiling approaches. We explore this phenomenon and show how it manifests in the loss of multiple biological functions in parasitic clades. The paper is accompanied by a webserver that allows a user to explore the functional interaction predictions for all human genes. We present three analyses corresponding to those found in the paper: functional interactions for single genes and gene sets and function annotation for genes. This webserver can be accessed at: https://mlpp.cs.huji.ac.il

## Results

### Clade-wise phylogenetic profiling outperforms traditional approaches

Clade-wise phylogenetic profiling (PP) takes into consideration the co-evolution of genes in different evolutionary scales, from the kingdom to the species level^3,7,10^. In addition, it was shown that different pathway types might show different co-evolutionary patterns, e.g. metabolic pathways being more conserved throughout evolution while signaling pathways often rewire^14^. Accordingly, we sought to utilize clade-wise PP to improve the predictive power of PP and enable the prediction of the interaction context by developing a supervised machine-learning based approach (Machine-Learning based Phylogenetic Profiling—MLPP). This approach integrates the phylogenetic profiling signals from 49 clades throughout 1154 species encompassing the eukaryotic tree of life. As the tree of life is hierarchical by nature, the clades for the analysis were chosen to cover the entire eukaryotes spaces, while reducing overlap and co-linearity between input features (see “Methods”, Supplementary Table 1, Supplementary Fig. 1).

We first computed a species-by-gene matrix representing the sequence similarity of every gene in each species to its human ortholog, with a given row comprising the phylogenetic profile of a single gene (see “Methods”). Then, for each clade, we calculated the covariance between the phylogenetic profiles for each pair of genes as features to the machine-learning algorithm. Thus, for each gene pair, we used 49 clade-wise covariances of the genes’ phylogenetic profiles as features. We then trained a binary classification model to predict gene pair functional interactions defined as co-occurrence in any Reactome pathway^20^. We used the same 49 features to train additional models to predict the interaction contexts for each context separately. The interaction context refers to the ways and functions in which genes are functionally related and is hereby defined as the co-occurrence of genes in some pathway type (12 Reactome top-level pathways, e.g., Metabolism, Immune System and 28 high level Gene Ontology terms) or protein complex co-occurrence in Reactome (see “Methods”)

We compared the performance of several machine-learning algorithms and positive-unlabeled frameworks^21–24^, choosing a random forest classification algorithm (similar to Claesen et al.^23^) on the basis of performance and robustness to unlabeled data (see “Methods”, Supplementary Methods, Supplementary Figs. 2, 3). To determine the added benefit of using clades in comparison to random sets of organisms, we compared the real clades to randomized clades. The comparison revealed that the tree structure and clade-specific evolution are indeed important to the performance of the method (Supplementary Text 1, Supplementary Table 2). The method is also robust to the choice of blast pre-processing (See “Methods”, Supplementary Table 3).

**Fig. 1:**
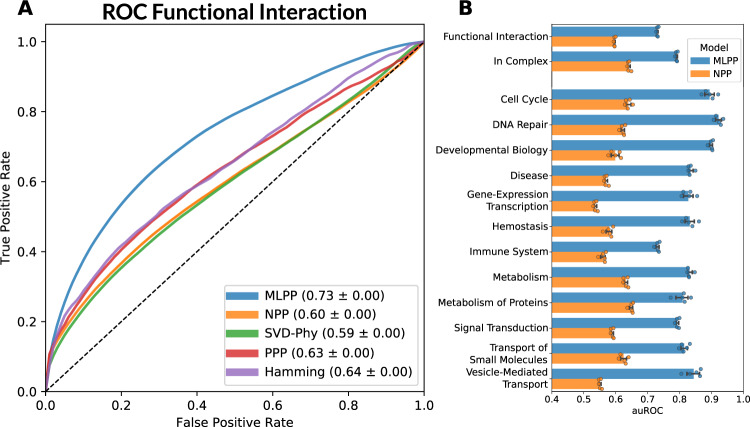
Method comparison for prediction of functional interactions. Our model (MLPP) was compared against other phylogenetic profiling approaches in terms of the receiver operator curve (ROC) and area under the curve (AUC) in predicting pairs of functionally interacting genes. Additionally, MLPP could predict the interaction context - complex co-occurrence, or one of 12 top-level pathways from Reactome. The model outperformed other approaches in predicting functional interaction (**A**, **B**) and the interaction context (**B**) when compared in 5-times cross-validation. Error bars denote the 95% confidence intervals using 1000 bootstrap samples. MLPP—machine-learning phylogenetic profiling, NPP—normalized phylogenetic profiling, SVD-Phy—singular value decomposition phylogenetic profiling, PPP—PrePhyloPro, Hamming—binarized phylogenetic profiling with Hamming distance. Source data are provided as a Source Data file.

To test the performance of our method, we compared it to four established PP methods: normalized Phylogenetic Profiling (NPP)^1^, SVD-Phy^25^, PrePhyloPro (PPP)^26^ and the Hamming distance on a binarized phylogenetic profile (BPP)^9,26^. These four methods do not take clades into consideration and are based solely on similarity metrics between genes’ phylogenetic profiles. We showed that our method, trained on functional interactions from Reactome, outperformed the others in terms of auROC (Fig. 1A) as well as partial auROC (at FPR < 0.1) and average precision (Supplementary Table 4, Supplementary Fig. 5), achieving a 14%, 3%, and 10% increase relative to the next best methods, respectively.

As several biases may obscure this comparison, we performed cross-validation with stratification in addition to random allocation to train and test splits. Previous studies found that functional interaction prediction models tend to overfit to genes found in pairs in both the training and test set (but not in the same pairs). Gene pairs in the test set were thus stratified to having both, just one, or neither of the genes in the training set as previously suggested^27^ (see “Methods”, Supplementary Fig. 5, Supplementary Table 4). In addition, genes with high sequence similarity (for example, paralogous genes) tend to be functionally related and co-evolved. However, this relationship is easily captured without PP and thus produces optimistic results for predicting functional interaction. We thus stratified gene pairs for this phenomenon (see “Methods”). When filtering out gene pairs with high-sequence similarity, differences in performance were even more pronounced (Supplementary Table 4, Supplementary Fig. 5). Another consideration is that of gene age. More recent genes (i.e. first appeared in a common ancestor closer to humans) may prove more difficult for co-evolutionary based methods. This can be attributed to greater similarity between closer organisms, leading to high phylogenetic profiling similarity between these genes regardless of function. We thus stratified for this phenomenon and showed that indeed the model’s performance is reduced for the subset of genes found only in Metazoa and Chordata (See “Methods”, Supplementary Fig. 4). However, these gene pairs constitute only a small portion of functional interactions in Reactome (1% for Metazoa specific and 0.5% of Chordata specific, mutually inclusive) and a high percentage of paralogous pairs (20% for Metazoa, 17% for Chordata, 5% for all genes).

In addition to functional interactions, our model predicts for each gene pair the interaction context. Previous studies showed that interactions belonging to different interaction contexts may show a globally different phylogenetic profile^14^. The interaction context represents additional information about the functional interaction of a gene pair, such as the pathway type. We showed that our approach outperformed the other PP methods in predicting pathway types from Reactome and high-level terms from GO and achieved high auROC, partial auROC and average precision in cross-validation and stratifications as described above (Fig. 1B compared to NPP, see Supplementary Information for additional comparisons).

Further comparisons of temporal splits and external validation databases revealed similar gains in performance. We assessed the performance of our functional interaction model, which was trained on Reactome (Feb. 2019), in predicting functional interactions from a future version of Reactome (Jan. 2021). Our model was robust to these temporal changes (Supplementary Fig. 6). Additionally, we externally validated our functional interaction model performance, trained on functional interactions from Reactome, in predicting functional interactions from both datasets similar and dissimilar to Reactome. Our model was robust for predicting PPIs from BioGrid—the Biological General Repository for Interaction Datasets^28^ (Supplementary Fig. 7A–D) and from IntAct—the EMBL-EBI Molecular Interaction Database^29^ (Supplementary Fig. 7E–H); functional interactions from the Kyoto Encyclopedia of Genes and Genomes (KEGG) database^30^ (Supplementary Fig. 8); and protein complex co-occurrence from CORUM^31^ (Supplementary Fig. 9A–D), and IntAct Complex^29^ (Supplementary Fig. 9E–H) databases. For protein complex co-occurrence, we also compared PP approaches to the “In Complex” interaction context model trained on complex co-occurrence in Reactome (Supplementary Fig. 10). These external validations were robust for each dataset both at the whole dataset and excluding functional interactions found in Reactome, and when excluding functional interactions between paralogous genes.

**Fig. 2:**
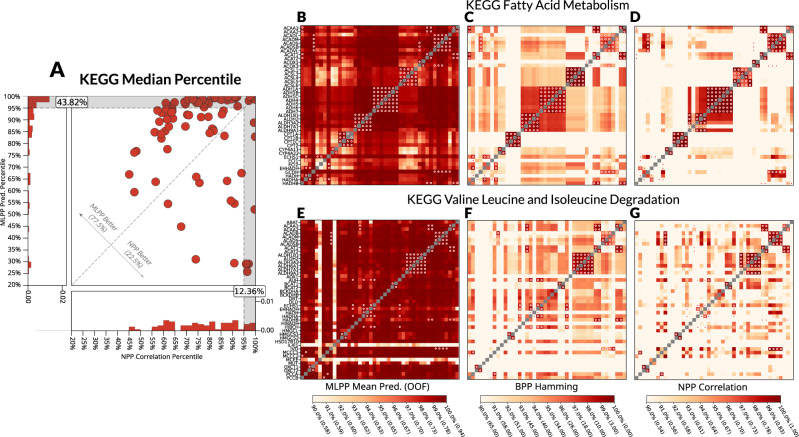
Method comparison at the pathway level. The MLPP model was compared to the NPP model in terms of predicting pathways by the median percentile of the score in the pathway on the KEGG database (**A**). Specific examples of fatty acid metabolism (**B**–**D**) and valine leucine and isoleucine degradation (**E**–**G**) pathways are given, comparing MLPP (**B**, **E**), BPP (**C**, **F**) and NPP (**D**, **G**). MLPP—machine-learning phylogenetic profiling, NPP—normalized phylogenetic profiling, BPP—binarized phylogenetic profiling with Hamming distance. For D-I, the color represents the percentile with scores per method in brackets, while red dots are paralogous proteins. Source data are provided as a Source Data file.

As phylogenetic profiling is commonly used to understand functional interactions at the pathway level, we compared the different methods at this level. For each pathway, we calculated the pairwise score for all pairs of genes in the pathway. To enable a comparison between the different methods, the scores of all gene pairs for each method were normalized by conversion to percentiles. Comparing the median percentile per pathway for all KEGG pathways, MLPP (functional interactions model) outperformed the NPP method in 77.5% of cases and identified 43.8% of pathways at the 95% percentile level (Fig. 2A, Supplementary Fig. 11A). For example, for the KEGG^30^ pathway Fatty Acid Metabolism, MLPP predicted its pairwise interactions at higher percentiles (Fig. 2B, the redder, the higher the percentile) than the BPP (Fig. 2C) and NPP (Fig. 2D) methods. A similar comparison is shown for the KEGG Valine Leucine and Isoleucine pathway (Fig. 2E–G). Larger gains in performance occurred when accounting for sequence similarity (Supplementary Fig. 11C) and similarly when compared to the BPP method (Supplementary Fig. 11B, D) and when comparing using the CORUM database of complexes (Supplementary Fig. 11E–H).

**Fig. 3:**
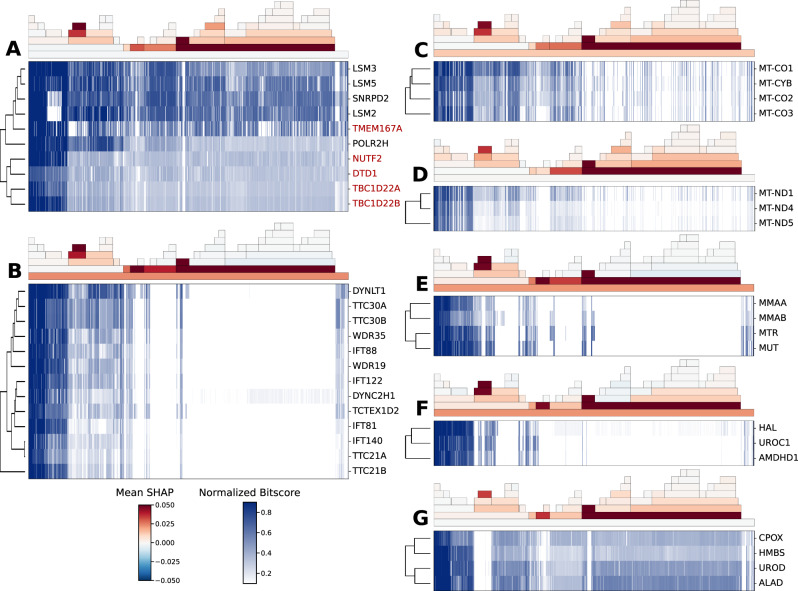
Co-evolved gene clusters. Model predictions for functional interaction were clustered using hierarchical clustering and cut at specific heights to produce clusters. For each of the clusters A–G, the top part is the clade importance for each clade calculated using the mean SHAP value, with species ordered from close (left) to distant (right) from human. The bottom part is the phylogenetic profile as self-hit normalized bitscores. Subfigures correspond to the co-evolved genes in the pathways mRNA splicing (**A**), cilia (**B**), mitochondrial respiratory complexes III and IV (**C**), NADH dehydrogenase (**D**), B12 metabolism (**E**), histidine metabolism (**F**) and heme biogenesis (**G**). Red entries in A denote genes not known to take part in mRNA splicing.

We then applied our method to identify modules of functionally interacting genes. For this, we clustered genes by their predicted functional interactions (i.e. predicted probability of interaction across all gene pairs) and extracted tightly interconnected modules by hierarchical clustering (see “Methods”). We identified many modules of known functionally interacting genes (Fig. 3). Some of the modules, such as ciliary^5,14^ and heme biogenesis genes^2^, were described previously as highly co-evolved (Fig. 3B, G, respectively). However, we also identified clusters where the signal was indeed contained in only a subset of the clades and thus more easily found by our method. For example, the mitochondrial respiratory complex III and IV genes (Fig. 3C) and NADH dehydrogenase (Fig. 3D) have a strong co-evolutionary signal in Fungi. Other clusters, such as the B12 metabolism cluster (Fig. 3E) and the Histidine catabolism cluster (Fig. 3F), show signals in both Fungi and Nematoda. Finally, the cluster in Fig. 3A contains genes related to mRNA splicing as well as some genes with no previous association to splicing (in red). Many of the modules found contained mostly genes with high sequence similarity (Supplementary Fig. 12) such as alcohol dehydrogenase enzymes (A), receptor/ion channel subunits (B, D, G), ribosome subunits (E, F), the exosome (C), collagen subunits (H) and histones (I). As previously stated, these modules were expected as genes with high sequence similarity are highly co-evolved and are often functionally related.

### Functional annotation of the Ignorome

Next, we applied our method to predict biological function for less studied genes. Many genes (termed the “Ignorome”) are less studied and rarely mentioned if at all in the literature^15^. This makes understanding their function challenging. Our systematic and unbiased approach does not depend on additional data, generalizes well to genes found only in the test set (see “Methods”) and, accordingly could help capture the function of the Ignorome. Hence we focused on genes lacking functional annotation in Uniprot^32^. These genes were additionally required to be in the lower 20% of PubMed mentions (less than 10 papers) or belong to ~2000 genes identified by the neXtProt consortium as genes with an unknown function^18^ (Supplementary Fig. 13).

**Fig. 4:**
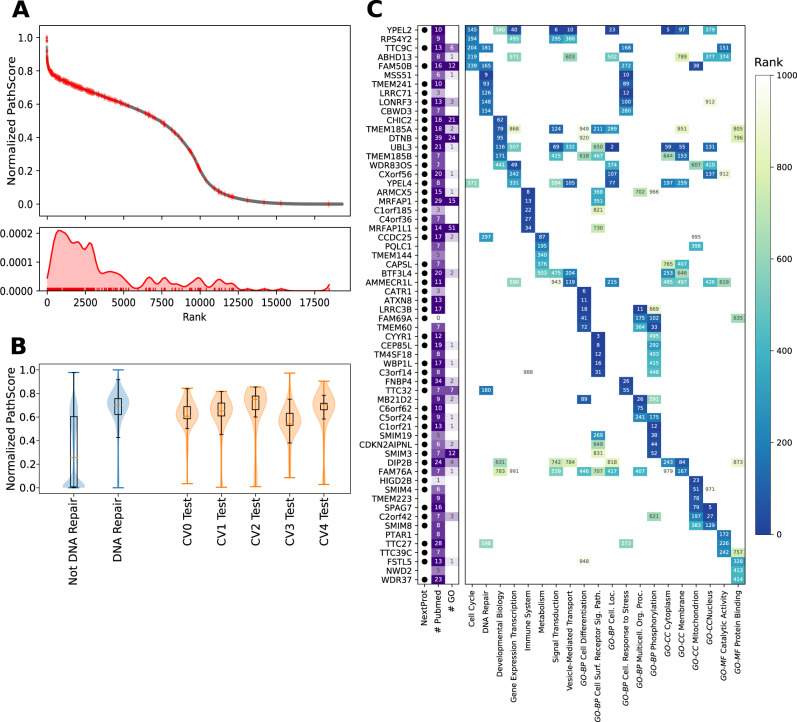
Assigning genes with unknown functions to pathways types. PathScore is a random-walk based measure identifying the importance of genes in a given network. The PathScore is calculated per label for all genes and prioritizes genes not known to belong to that pathway (gray) having equivalent or higher PathScores than known genes (red). Shown for the pathway type DNA repair (**A**). The PathScore is higher for genes belonging to a specific pathway and generalizes genes in the pathway found only in the test set. Shown for DNA repair (**B**, 240 known genes). Similar performance metrics appear in Supplementary Figs. 14-15 for the rest of the pathway types considered. The boxplot extends from the lower to upper quartile values of the data, with an orange line at the median. Whiskers denote 1.5 times the interquartile range. The top five less studied genes (having no function in UniProt in addition to low number of Pubmed mentions or appearing in NextProt uncharacterized genes set) are picked out for each pathway type and presented with their descending PathScore rank for the pathway types from Reactome and GO (**C**). Genes are annotated by whether they appear in the uncharacterized gene set in NextProt (by a dot to denote appearance in the list), number of Pubmed mentions (“# Pubmed”) and number of associated GO terms (“# GO”). GO—gene ontology. Source data are provided as a Source Data file.

To predict gene function, we utilized a random-walk based prioritization of genes, which we call the “PathScore”. Using our MLPP approach described above, we generated the full predicted functional interaction network for each of the interaction-context pathway type models. We then scored genes according to the equilibrium distribution of random walks on this network (see “Methods”). The score given for each gene signals its importance for this pathway type according to its connectivity in the predicted functional interaction network (see “Methods”). Shown for DNA Repair, PathScore ranks genes known to belong to this pathway type at the top (Fig. 4A) and is robust across train and test splits in multiple cross-validations (Fig. 4B, similar analyses can be found for the rest of the interaction context models in Supplementary Figs. 14,15). By inspecting the less studied genes, we identified tens of genes at the top 250 PathScore ranks for each pathway type, yielding one or more annotations to 238 Ignorome genes (Fig. 4C, Supplementary Data 3).

Specifically, for DNA-repair type pathways, we identified several potentially functionally related genes. Out of the first 50 genes ranked for DNA repair, we identified nine genes annotated as related in Reactome (22 for top 200). In addition, we identified nine genes that are known to be related to DNA repair but were not found in Reactome. These include *EXO5* (rank 8), an exonuclease related to DNA-repair and genome stability^33^; *C17orf53* (rank 16), previously an Ignorome gene, which was recently identified to be involved in homologous recombination repair^34^; the DNA polymerase *DNTT* (rank 18); the telomerase *TERT* (rank 23); the SMC5-6 complex gene *SMC5* (rank 47, also *EID3* in rank 55)^35^; and genes previously prioritized as related to DNA repair (*ELP6*^36^—rank 33, *PIGN*^37^—rank 34, *NUDT15*^38^—rank 36, *STK19*^39^—rank 46). These serve as a strong external validation of the PathScore prioritization approach. In addition we identified 18 genes in the top 200 that were prioritized by several CRISPR assays to be related to DNA repair^39^ (rank in brackets)—*GPATCH8* (3), *SCNM1* (7), *OMA1* (10), *AOC2* (50), *RCE1* (71), *ALG3* (92), *THUMPD1* (111), *DPH6* (115), *PIGW* (119), *TYW1* (131), *VPS16* (132), *PPOX* (143), *DUSP12* (146), *ISCA2* (158), *NAALADL2* (187), *POLA2* (194).

This approach also highlighted hundreds of genes that may be functionally related to several pathway types. Overall, we identified 1554 non-Ignorome and 58 Ignorome genes at the top 250 ranks for more than one pathway type. For example, the Yippee-type proteins *YPEL1*, *YPEL2*, and *YPEL4* were ranked high in Cell Cycle, Disease, Gene Expression, Homeostasis, Metabolism of Proteins, Signal Transduction, Transport of Small Molecules and Vesicle-mediated Transport with *YPEL1* and *YPEL2*, ranking in the top 250 for these eight pathway types. The Yippee family proteins are putative zinc-fingers known to be related to the centromere^40^.

### MLPP uncovers evolutionary insights underlying pathway co-evolution

**Fig. 5:**
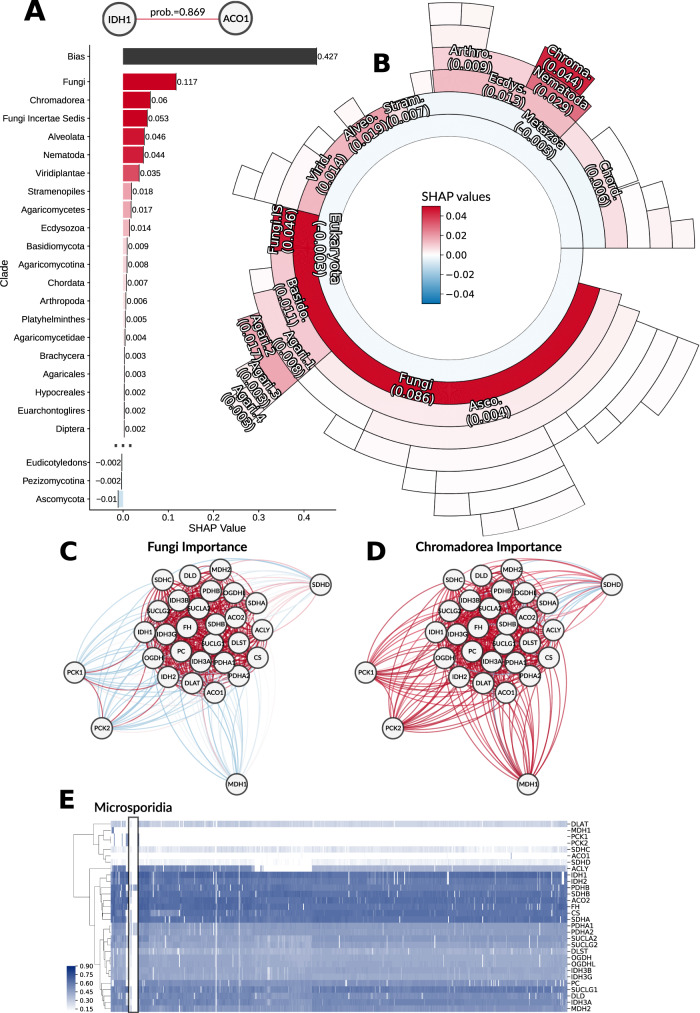
Evaluating clade importance—citric acid cycle case study. SHAP values were calculated for all gene pairs in the Citric Acid Cycle from KEGG. A specific example is provided here for the gene-pair *ACO1*-*IDH1* (**A**). Bars represent the SHAP values calculated for the specific clade and colored by the value. SHAP bias term is the probability without knowing the value for any of the clades. Mean SHAP values were calculated across all gene pairs (**B**). Shown are clades with a mean SHAP value above 0.002 and colored by a mean SHAP value. Species go from close to human at 0 degrees to more distant counter clockwise. Clade abbreviations are found in Supplementary Material. All pairwise interactions are shown in a network for Fungi and Chromadorea, the top two most important clades by SHAP values (**C**, **D**, respectively). Edges are colored similar to clades in A. The normalized bitscore matrix for Fungal species for all genes in the pathway highlight their loss at the Microsporidia clade (**E**).

Phylogenetic profiling can be backtracked to produce evolutionary insights into pathway evolution. These insights include important loss events^5,12^ and analyses on different pathway types^14^. Our method enables similar evolutionary inference by calculating the contribution of each clade (feature) to the prediction of functional interaction for a gene pair. Clade contribution to the prediction is calculated using the SHAP method for tree-based models^41,42^. SHAP values are calculated by considering the change in predictions when the clade is present or absent from the model through all possible combinations. For example, for the gene-pair *ACO1*-*IDH1* from the citric acid cycle (TCA), the probability for functional interaction is 0.87. The probability can be decomposed by SHAP to 0.117 for Fungi, 0.06 for Chromadorea, −0.01 for Ascomycota, and so forth (with a bias term of 0.427, Fig. 5A; clades with a SHAP value less than 0.002 in absolute value are not shown). The interpretation of these values is conceptually similar to the interpretation of the coefficients and intercept (bias) of a linear model. The evolutionary inference is made at the clade level and thus cannot point to the timing of specific loss events such as previously described, e.g. in ref. ^5^. Nevertheless, it can reveal clades where loss events may have happened, the pathway’s first introduction, or loss of co-evolution at the common ancestor level. Moreover, our model allows for a unified assessment of these evolutionary insights across all gene pairs, pathways and pathway types. We thus present insights into functional interactions and pathway evolution at these three levels.

As an example, we focus on the citric acid cycle (TCA) pathway. The model identifies Fungi and Chromadorea as the clades with the highest importance for predictions in this pathway (Fig. 5B). These clades are complementary in predicting some of the functional interactions. While Fungi is the single most informative clade, it failed to predict the interactions of the genes *PCK1* and *PCK2* with the rest of the TCA genes. However, these interactions are well captured in Chromadorea (Fig. 5C, D). This may relate to the function of *PCK1/2* as these genes control gluconeogenesis from TCA intermediate metabolites and are thus peripheral in the pathway.

Overall, the phylogenetic profile of TCA in Fungi revealed that most genes are conserved throughout the clade except for the known loss of the pathway in the Microsporidia parasites^43^ (Fig. 5E, demarcated with a box). Thus, the model links the phenotypic change in Microsporidians to the loss event by utilizing the importance measure provided by our method, demonstrating its applicability in identifying evolutionary insights. Two additional examples of pathway evolutionary insights are provided; methylmalonic acid metabolism and histidine metabolism (Fig. 3E, F, respectively, the top part of each subfigure). In these pathways, the model identified specific loss events in nematodes by clade importance. Overall we showed that our model could capture specific loss events similar to those found in the previous approaches^5^.

**Fig. 6:**
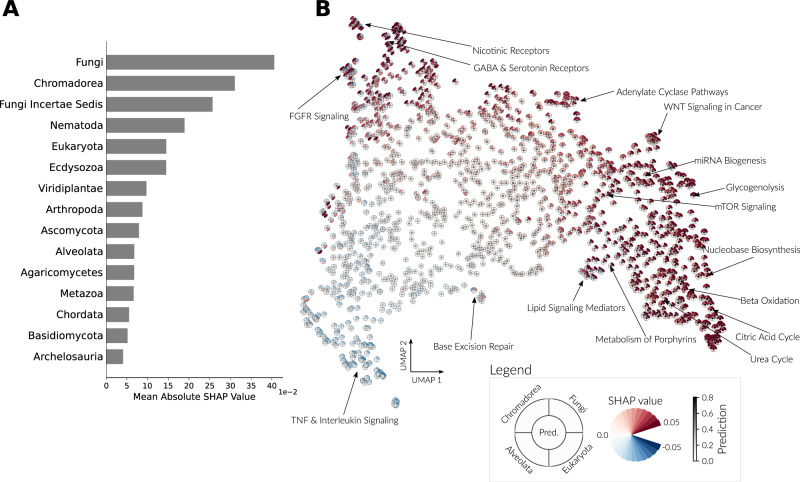
Clade importance yields evolutionary insights. Clade importance by SHAP values was calculated for the test set of a single cross-validation in the functional interaction model, revealing the clades with the highest mean absolute importance (**A**). Clade importance was averaged for each Reactome pathway and projected into two dimensions using UMAP (**B**). For each pathway, a marker is shown with the average SHAP value for one of four specific clades by the color of a quarter of the marker. The average predicted probability across gene pairs is shown by the color of the circle in the middle. Source data are provided as a Source Data file.

Next, we sought to derive insights at a higher level of pathway co-evolution. We first assessed the general informativeness of clades in predicting functional interactions. Overall, for functional interactions the most critical clades were Fungi (mean absolute SHAP value of 0.04), Nematoda (0.022) and their subclades Fungi Incartae sedis (0.03) and Chromadorea (0.033) (Fig. 6A, from the top by decreasing importance as the mean absolute SHAP values). Unexpectedly, these specific clades had a higher importance than using all Eukaryotes (0.018), suggesting that specific clades may prove more informative in general, both for our approach and for phylogenetic profiling in general.

For the interaction context, different pathway types had different clade importance (Supplementary Fig. 16). It was initially expected that the more “ancient” pathway types would rely more on distant organisms and vice versa. This hypothesis was recapitulated in our analysis. For example, the Metabolism model assigns higher importance to distant clades such as Alveolates, Stramenopiles, Fungi, and all Eukaryotes, while the Immune System model assigns higher importance to clades closer to humans such as Metazoa and Ecdysozoa. However, some pathways displayed a counterintuitive attribution of clade importance. For example, the Signal Transduction model assigns high importance to all Eukaryotes while expected to rewire often and thus be more informative in organisms closer to human.

We then examined the clade importance of gene pairs and pathways. For this, average SHAP values per pathway were projected using UMAP^44^. This analysis clusters together pathways with a similar clade importance attribution (Fig. 6B). For example, many metabolic pathways (Fig. 6B, bottom right) give high importance to Chromadorea and Fungi. Similarly, a group of receptor types, complexes, and signaling pathways give high importance to all Eukaryotes and Chromadorea (Fig. 6B, top left). These differences highlight the added value of clade-wise phylogenetic profiling, which is able to detect co-evolution in subsets of the Eukaryotic tree. UMAP projection of gene-pair SHAP values identified similar insights. Here, clusters of gene pairs with similar clade importance show that the highest-scoring pairs gave high importance to both Fungi and Nematoda (Supplementary Fig. 17).

Overall, our method enables one to uncover specific patterns across gene pairs, specific pathways, and all pathways level thus shedding light on pathway evolution. These insights can be categorized into two types. First, identifying clades with gene loss events that translate to meaningful phenotypical effects and, second, shedding light on the underlying evolutionary processes behind pathways of various kinds. These include per pathway gain, differences among clades in pathway losses and the informativeness of various clades for phylogenetic profiling of functional interactions in general and specific pathway types particularly.

### Analysis of parasitic organisms’ signal in phylogenetic profiling

Many of the most informative clades described above, such as Chromadorea, Stramenopiles, Alveolata, and Fungi Incertae Sedis contain a large percentage of parasitic organisms. Parasitic organisms are known to undergo vast gene losses and drastically diverge from their free-living counterparts^45–48^. We thus hypothesized that these insights may be related, identifying parasitic organisms across the tree of life as a key signal in phylogenetic profiling.

**Fig. 7:**
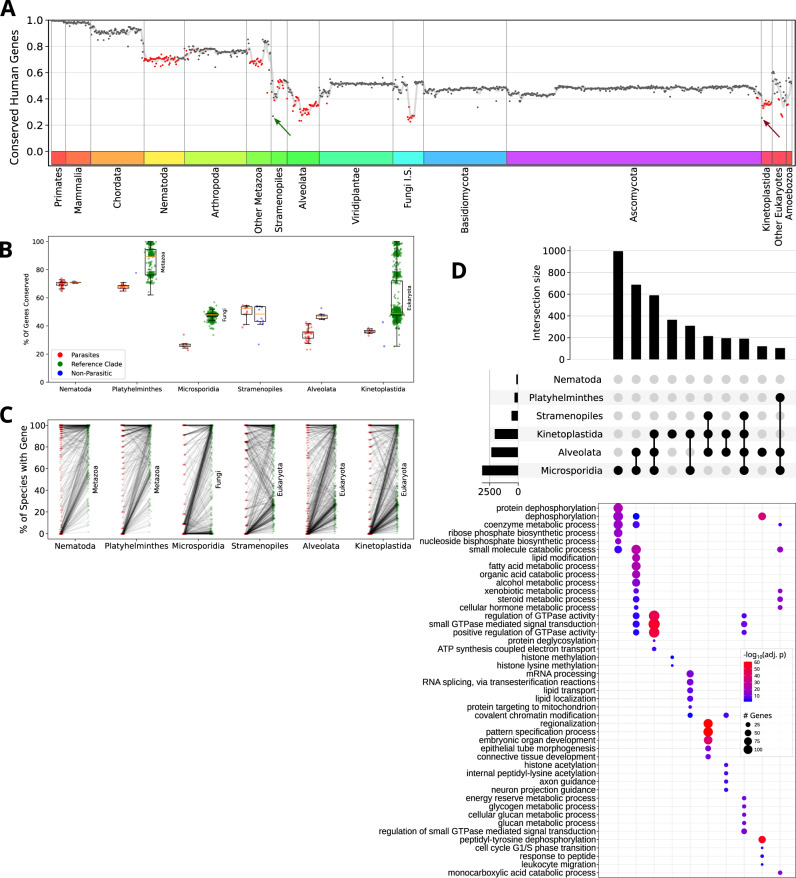
Parasitic clades provide a key phylogenetic profiling signal. The proportion of human genes found in each organism is shown on the y-axis, with parasites marked in red. Two non-parasitic organisms with lower conserved genes fraction are highlighted in green (*Nannochloropsis gaditana*, in Stramenopiles) and red (*Perkinsela*, in Kinetoplastida) arrows (**A**). The fraction of conserved genes was then compared for six clades with many parasitic organisms between parasites, non-parasitic organisms and a reference (parent) clade. Comparisons were made for each clade between parasitic organisms and the reference and non-parasitic organisms by a two-sided Mann–Whitney test; p-values are displayed for significant comparisons (*p* < 0.05). The boxplot extends from the lower to upper quartile values of the data, with an orange line at the median. Whiskers denote 1.5 times the interquartile range. (**B**). In addition to the species level, comparisons were made by pairing the average conservation for each gene in the parasitic organisms (red) and reference clade (green) with a line connecting them (**C**). Genes that were fully lost, or with low conservation in at least one parasitic clade but highly conserved across all Eukaryotes, were tested for losses combinatorics across these clades. Genes in the top 10 intersections were checked for gene ontology overrepresentation (biological process ontology). The top five terms by FDR adjusted p-value are shown for each combination (**D**). The upper panel presents the number of genes in each clade or intersection, with the relevant clades marked by black circles. The lower panels show the number and significance in the most relevant pathways. FDR—false discovery rate. Source data are provided as a Source Data file.

Parasitic organisms (see “Methods”, Supplementary Table 5) are generally less conserved with respect to humans than free-living organisms (Fig. 7A, in red). The lowest percent of orthologs are found in parasitic organisms in Alveolates, Microsporidia (Fungi Incertae Sedis, denoted as Fungi I.S) Kinetoplastids and intestinal flagellates (Hexamitidae, denoted as other eukaryotes) (Fig. 7A, in red). Only two organisms show similar loss levels, one micro-algae (*Nannochloropsis gaditana* in Stramenopiles, marked with a green arrow) and one endosymbiotic Kinetoplastida (*Perkinsela*, marked with a red arrow). For the endosymbiont, the same rational of gene loss related to host adaptation was previously described^49^.

Six parasites containing clades were further compared to non-parasitic (free-living or mutualistic) organisms in the same clades and a reference parent clade (Fig. 7B, C, a full list of organisms considered parasitic can be found in Supplementary Table 5, see “Methods”). Parasitic clades showed a statistically significant reduction in conservation level (as compared to human) from both the non-parasitic organisms (Nematoda, Alveolata) and the reference clade (all but Stramenopiles, 2-sided Mann–Whitney test, Fig. 7B). Moreover, many of the lost genes in parasitic organisms were highly conserved in non-parasitic organisms in the reference clade (Fig. 7C).

Thus, we were interested in mapping the genes lost in each clade and how this signal translates across clades and into pathways. We analyzed genes that are highly conserved in all Eukaryotes but have low conservation in at least one parasitic clade (see “Methods”), ending up with 4114 genes (Fig. 7D). The presence and absence of each gene in each clade were considered, and all clade combinations underwent enrichment analysis for Gene Ontology (GO) biologic processes (see “Methods”). Parasitic clades often lack orthologs for specific metabolic, signaling, and developmental pathways (Fig. 7D, top 10 clade combinations by number of genes in the intersection). By inspecting the different combinations, we found that some pathways were enriched in certain combinations. For example, mRNA splicing genes were lost both in Kinetoplastida and Microsporidia, while GTP signaling genes were lost in either the 2nd, 3rd, or 8th combination (from the left), all consisting of Microsporidia and Alveolates. Hence, some pathways can be identified by their loss pattern in parasitic organisms.

However, a sensitivity analysis of excluding parasitic organisms in the training of the method results in conflicting evidence. On one hand, while many of the clades with highest importance contain parasites (as mentioned above), excluding parasites or these clades causes only a mild reduction in performance (see “Methods”, Supplementary Table 6). On the other hand, clade importance does indeed shift from these clades to others (Supplementary Fig. 18). This suggests that while parasitic organisms do indeed contribute to the model presented in this work, other signals exist in phylogenetic profiling which can reach a similar performance.

## Discussion

Evolutionary approaches are one of the primary sources to understand gene function and interactions^1–6,8–10^. Here we present a machine-learning approach to phylogenetic profiling and demonstrate its utility in predicting functional interactions. By using clade-wise phylogenetic profiling, our approach predicted genes’ functional interactions and revealed evolutionary insights underlying the prediction. We applied our method to predict putative functions for less studied genes and explored the evolutionary signal found in parasitic organisms.

Our method extends phylogenetic profiling by using machine-learning to recovering signals found within individual clades, in addition to using the whole eukaryotic tree of life. This clade-wise phylogenetic profiling approach hypothesizes that for some functional interactions, the co-evolutionary signal is better captured in a subset of the tree. This can stem from genes first appearing later in evolution (e.g., metazoan or mammalian specific) or, more interestingly, from genes that only function together in a subset of the tree. Opposite cases may also exist, where a pathway dissembles, i.e. consists of genes found in human that are not functionally related and are thus no longer co-evolving in a specific clade, while still co-evolving in other clades.

Our work goes beyond previous insights into the utility of clade-wise phylogenetic profiling, which were limited in their clade resolution or applicability to the whole interactome^3,7,10^. We present a fully formed clade-wise phylogenetic profiling approach that, together with a supervised machine-learning approach, considerably improved the performance in predicting functional interactions.

In addition, our approach provides insights into the importance of clades for specific predictions, thus identifying the underlying evolutionary signal. These evolutionary insights are essential for understanding how pathways may have evolved and produce a broader perspective on predictions. This provides an practical and scalable alternative to computationally expensive methods for performing full inference on the phylogenetic tree^5,19,50^. We present here evolutionary insights on several distinct levels. At the single pathway level, our approach identifies clades with a strong co-evolutionary signal (such as independent loss events). This is analogous to the insights derived from full inference on the phylogenetic tree. By inspecting clade importance for interaction context models, we recapitulate hypotheses stated in the literature such as having more recently evolved pathway types (e.g., immune pathways) assign more importance to metazoan clades. Finally, at the macro level, we consider which clades are the most important for prediction and, by proxy, the most evolutionary informative. Our analyses extend the variety of evolutionary insights available from phylogenetic profiling, going all the way from pairwise functional interactions to mapping evolutionary insights for all pathways together.

Several of the most important pathways identified by our method contain parasitic organisms. However, a different hypothesis may be considered in which clades with high variability yield the strongest signal, while parasitic species provide only one such example. This is further corroborated by our analysis of the exclusion of parasitic organisms from model training. As an illustration, we found that nematodes are one of the most important clades for the model, where nematodes have been previously identified to be highly divergent concerning their evolutionary distances regardless of parasitic status^51,52^

Many human genes are still mostly uncharacterized. In recent years this problem garnered attention and solutions were rigorously discussed^15–17^. One such solution is characterizing these genes by utilizing unbiased (or minimally biased) information from mRNA-sequencing and other high-throughput experimental approaches^15,17^. Phylogenetic profiling provides one such unbiased approach to understand gene function through functional interactions captured by co-evolution. Although not completely unbiased, our approach predicts many promising functional annotations for less studied genes that can be further explored through computational and experimental techniques. Particularly, for DNA repair, external validation reveals that many prioritized genes are indeed related, paving the way for research on other top ranked genes in that list.

However, some limitations of our method remain. First, the low performance of our method for young genes inhibits the utilization of our approach for such genes. This is possibly caused by the high reliance of our method on more distant clades and not on the lack of a co-evolutionary signal. Second, the supervised nature of our approach suggests that it may be less suitable for less studied genes. While our analyses on stratifications revealed that our method is indeed robust both in cross-validation and external validation on unseen genes, some performance loss is noted. This may be alleviated in future work by using unsupervised machine-learning approaches, such as autoencoders building on the work of SVD-Phy^25^. Finally, our approach for functional annotation, PathScore, was intentionally limited to large pathway types and is unsuited to small pathways due to the supervised nature. Modifying PathScore to utilize other network propagation algorithms, such as methods allowing for multiple specific seeds, may enable it to be utilized for smaller pathways, similar to other approaches in the literature^53^.

As many more species are sequenced, we believe that looking at evolution systematically and across different evolutionary scales using machine-learning based approaches will result in further improvements in performance and reveal predictions and evolutionary insights. This work emphasizes the major potential of massive genomic analysis in understating genotype-phenotype interaction and the crosstalk between gene, function and evolution. This work is accompanied by a webserver that enables exploration of the predictions in this paper, for both functional interactions and functional annotation prediction and is available at: https://mlpp.cs.huji.ac.il.

## Methods

### Phylogenetic profile construction

To model gene co-evolution, we generated gene phylogenetic profiles similar to those described previously^1–3,54^. First, we downloaded the proteomes of 1154 species (see Source Data) from UniProt as FASTA files using the programmatic API^32^ (accessed at 28.12.2018, https://www.uniprot.org/uniprot/?query=proteome:Proteome_ID&format=fasta). To further enrich these proteomes, we extended each species FASTA with its proteome from the NCBI’s RefSeq non-redundant protein database^53^ (accessed 25.12.2018, ftp://ftp.ncbi.nlm.nih.gov/blast/db/). We then constructed a reference set of human proteins such that each human gene had a single representative protein. We retrieved the human proteome from UniProt (accessed at 25.12.2018) and selected the corresponding longest protein for each human gene as a reference.

We used BLAST^55^ to align each human protein with its one-directional best hit in each of the 1154 species. For this, blastp was executed on the command-line^56^ (version 2.7.1) with the arguments “-max_target_seqs 1” to retain only the top hit per gene per species. The output of this step is a matrix B such that each element B_i,j_ contains the blast bitscore between a human gene (as a reference protein) *i* and the blast best hit at species *j*. Bitscores lower than a set threshold of 60 were set to zero. An analysis of several bitscore (40, 60, 100) and *E*-value (1e−3) thresholds is presented in Supplementary Table 3, showing robustness to the chosen threshold. We then normalized each row (gene) by dividing by the bitscore of the human protein self-hit to account for protein length and evolutionary distance from the reference organism^57^.

Taxonomic annotation of species was retrieved from UniProt taxonomy^32^ (accessed 01.09.2019, https://www.uniprot.org/taxonomy/). Taxonomic lineage information was split into clades, clades with less than 10 species were filtered out. Next, clades were sorted by decreasing size and were filtered based on Jaccard similarity such that clades with a Jaccard similarity greater than 0.8 with a larger clade were deemed as redundant and filtered out. Both the redundant and non-redundant set of clades can be found in Supplementary Table 1 (non-redundant marked in blue).

### Pathway and interaction data gathering and pre-processing

Pathways and gene sets were obtained from Reactome (Croft et al. 2011), Kyoto Encyclopedia of Genes and Genomes (KEGG)^30^, CORUM^31^ and UniProt GOA^58^. Reactome pathways were downloaded as a GMT file (reactome.org/download/current/ReactomePathways.gmt.zip, accessed 25.01.2019) with gene symbols, and pathway descriptions (reactome.org/download/current/ReactomePathways.txt, accessed 05.02.2019). For temporal splits, Reactome data was re-acquired 12.01.2021. Pathway hierarchy was downloaded as an adjacency list (reactome.org/download/current/ReactomePathwaysRelation.txt, accessed 05.02.2019). Reactome complexes were retrieved using the REST API and filtered to keep only protein components (queried at 12.02.2019). KEGG pathways were retrieved from MSigDB v6.2 as a GMT file with gene symbols (http://software.broadinstitute.org/gsea/downloads.jsp, accessed 28.11.2018). CORUM complexes were downloaded from the website and converted to a GMT file with gene symbols (https://mips.helmholtz-muenchen.de/corum/download/allComplexes.txt.zip, accessed 12.02.2019). UniProt GOA was downloaded as GAF (ftp://ftp.ebi.ac.uk/pub/databases/GO/goa/HUMAN/goa_human.gaf.gz, accessed 04.02.2019) and matched with the gene ontology term description to generate a GMT file^59,60^ (as OBO, http://geneontology.org/docs/download-ontology/, accessed 04.02.2019).

A standardized pre-processing pipeline was applied to each of the pathways and gene sets sources. Each GMT was filtered for a gene set with at least three genes and at most 50 genes. Next, an adjacency list was generated for each gene set assuming the gene set is fully connected (i.e. all pairwise connections were considered). These adjacency lists were joined to produce the list of all gene-pair pathway co-occurrences for each source. For Reactome, an additional step was taken by partitioning the pathways by top-level pathways. Top-level pathways were filtered keeping those with at least 5000 gene pairs, thus ending up with 12 top-level pathways remaining. A similar attribution of top-level pathways was produced for GO terms ending up with 28 pathway types – 17 GO BP (biological process), 6 GO CC (cellular compartment), and 5 GO MF (molecular function) terms.

Interaction data was obtained from IntAct^29^ and BioGrid^28^. IntAct complexes and BioGrid pairwise interactions (as SIF files) were retrieved from PathwayCommons^61^ (version 10, https://www.pathwaycommons.org/archives/PC2/v10/, accessed at 04.08.2018).

### Model training and prediction

Machine-learning models were trained in a multi-label one vs. all fashion using scikit-learn^62^ and lightGBM^63^. We compared a decision tree, logistic regression, naive Bayes and random forest (using lightGBM), and chose lightGBM, which achieved the highest performance (Supplementary Fig. 3). The lightGBM model was further tested on a different positive-unlabeled framework for predicting hidden positive links (Supplementary “Methods”). For each label (functional interaction, i.e. any pathway co-occurrence, or interaction context, i.e. specific pathway type co-occurrence or protein complex co-occurrence), a model was trained to predict the label using the covariance measured between the gene-pair profiles in each of the 49 clades (as described in Section 8.1). The model was trained in a five-fold repeated cross-validation (CV) fashion to assess performance. Each CV fold consisted of a random sample of known (positive) gene pairs matched with random negative pairs. Random negative pairs were chosen such that the number of genes in the negative pairs set approximately matched those in the positive pairs set to preserve the topology. For each cross-validation fold, a stratification was performed on the gene content in the train and test splits. Park and Marcotte^27^ have shown the influence of the individual genes in the interacting pair when performing a pairwise interaction prediction. Therefore, we performed a similar stratification to that suggested in their original paper by taking 30% of the genes in the positive and negative pairs as a designated test set only genes. We then split the test set into three: C1 – where both genes were present in some pair in the training set, C2—where only one of the genes in the pair was present in the training set, and C3—where neither were used in training. In addition, as paralogous genes have similar phylogenetic profiles and may act as “data leakage”, each gene chosen to be a test set exclusive was grouped with all genes with a high sequence similarity to it (bitscore > 60) from the genes in the CV fold, which were designated as test-set exclusive as well. The final lightGBM random forest model was used to predict probabilities for all ~200 million possible gene pairs in each of the labels both as the average across CV folds and for each CV fold by itself.

### Positive-unlabeled learning

Functional interaction prediction is a classic problem of positive-unlabeled learning. Annotations of functional interactions include only confirmed true interactions and lack confirmed non-interacting pairs. Supervised learning can be used in such cases, treating unlabeled pairs as negative; however, several approaches have been developed to treat the unlabeled data more appropriately^21–24^. Thus, we simulated a situation in which known positives are hidden (as unlabeled) and checked how well different methods recover these positives. We compared four methods (a) a vanilla light gradient boosting machine (LGBM), a standard LGBM classifier with unlabeled as negatives, (b) PUBag, a positive-unlabeled bagging of LGBM trees in which the positive are constant and the unlabeled are sampled for each model in the bagging procedure, inspired by Mordet and Vert et al. bagging SVM^22^, (c) AdaSample, an adaptive sampling approach in which negatives and positives are selected for each classifier in the ensemble by the probability of belonging to the class from the previous iteration^24^, and (d) a random forest model, LGBM RF, capturing similar characteristics to the SVM ensemble described by Claesen et al.^23^. The base classifier for PUBag and AdaSample was a default LGBM classifier with 10 trees. For the vanilla LGBM, the default LGBM classifier had 200 trees and for LGBM RF we used the random forest mode (“boosting_type = ’rf’”), 200 trees, subsampling of 0.5, feature sampling of 0.5, and a deeper tree with 128 “max leaves”. Performance was measured by area under the receiver operator characteristics curve (auROC) and by inspecting the probability distribution of positive, random negatives and hidden positives gene pairs. PUBag was run using a github implementation by R. Wright^64^ with subsampling of 0.5 and feature sampling of 0.5. AdaSample was implemented in Python by the authors based on the R implementation^65,66^ and ran for two iterations with a subsampling of 0.3 and a resulting ensemble of 20 models. We performed cross-validation and compared the models with four different hidden proportions: 0.1, 0.3, 0.5, and 0.7. For 0.3, 30% of positive pairs in the training set were considered as negatives (Supplementary Fig. 2).

### Phylogenetic profiling comparison

We compared our method (described in Section 8.3) to four established phylogenetic profiling methods: Normalized Phylogenetic Profiling^1,2^, Singular Value Decomposition of Phylogenetic Profiling (SVD-Phy)^25^, the Hamming distance on a binary phylogenetic profile (BPP)^9,26^, and PrePhyloPro^26^. For NPP, the matrix was prepared as described above (Section 8.1) and then further normalized by first taking the log_2_ of the matrix and performing standard scaling on the columns (species) by subtracting the column mean and dividing by the column standard deviation. Similarity between genes was calculated using Pearson correlation. For SVD-Phy, the matrix was prepared as described above and a truncated-SVD was calculated taking the first 35% of components as described in the original paper^25^. Similarity between genes was calculated by taking the Pearson correlation between the components. For the Hamming and PrePhyloPro matrices, blast output was taken as an *E*-value instead of a bitscore and was binarized by assigning 1 (ortholog present) to *E*-values less than 10^−3^ and 0 (ortholog lost) otherwise. For the Hamming distance binary profiles, the similarity between genes was calculated using the Hamming distance. For PrePhyloPro, the similarity between genes was calculated using the rank of the Jaccard index for a single gene against all other genes. As described in Niu et al.^26^, wherever the Pearson correlation between the profiles was less than 0, the rank was considered to be last.

Comparisons between our approach and other PP approaches are presented for functional interactions in Reactome (Fig. 1A, Supplementary Fig. 5, Supplementary Table 4), Interaction context models – Reactome complexes (Fig. 1B, Supplementary Fig. 5, Supplementary Table 4), pathway types from Reactome (Supplementary Data 2), pathway types from GO (Supplementary Data 2). Comparisons are also shown for “young genes” (Supplementary Fig. 4), Reactome temporal splits (Supplementary Fig. 6), external validations on various databases (Supplementary Figs. 7–10) and comparisons on the pathway level analyses (Fig. 2, Supplementary Fig.11).

### PathScore—random-walk based prioritization

To identify the centrality of genes in an interaction context-specific network, a pathway score (PathScore) was devised. The PathScore is the stationary distribution of random walks on the predicted interaction network for each label. Specifically, the predicted probabilities for a specific label are first sparsified by turning all values below the 75% percentile to 0. Then each row is divided by its sum (i.e. node degree) to create a stochastic matrix. Next, the stationary distribution is calculated using the power method and then scaled to the range [0–1] to generate the PathScore. In addition to the PathScore value, its decreasing order rank is used, e.g. see Fig. 4C. For each pathway type, the precision of PathScore at rank 100 was calculated with pathway types with precision less than 10% discarded, yielding 30 pathway types (Supplementary Data 3, Supplementary Fig. 15).

### The ignorome—less studied genes

Less studied genes were defined as uncharacterized proteins in neXtProt (Duek et al. 2018, Accessed 03/04/2019, https://www.nextprot.org/about/human-proteome page bottom, or query ID NXQ_00022) and genes with low PubMed mentions from gene2pubmed (Accessed 03.04.2019, ftp://ftp.ncbi.nlm.nih.gov/gene/DATA/gene2pubmed.gz). Both were filtered by genes present in the phylogenetic profiling matrix by a gene symbol and having no functional annotation in UniProt. Gene2pubmed was further filtered to keep only the bottom 20% of PubMed mentions, resulting in genes with 10 publication mentions or less.

### Clade importance

As the model of choice was lightGBM, feature importance could be calculated using Shapley values as implemented in the SHAP method for decision-tree-based models^41,42^. The SHAP method uses the Shapley value, a game-theory based method of credit attribution in a multi-agent collaborative process. In the case of feature importance, each feature (in this case covariance in a specific clade) is considered as an agent and each individual prediction a collaborative process involving these features. SHAP values can be positive or negative and can be thought of as “does this particular feature increase or decrease the likelihood of the prediction?”. To calculate SHAP values, the method “predict_proba(X, pred_contrib = True)” of the lightGBM classifier object was used. As the model was a random forest model, the SHAP values are given as the sum of probability contributions for each of the trees in the ensemble. Accordingly, the SHAP value was averaged across the trees to obtain the predicted probability contribution for the ensemble prediction.

Clade-importance projections were made using the UMAP^44^ python package. The parameters used were n_neighbors = 10, min_dist = 1, spread = 3. A UMAP projection into two dimensions was applied to the mean SHAP values corresponding to the 49 clades for each gene pair in each pathway in Reactome. It was then plotted with a custom matplotlib script to create Fig. 6B. For Supplementary Fig. 17, clade SHAP values were used for all gene pairs in the test set of the first cross-validation fold.

### Young genes

For analysis of model performance by gene “age”, genes were categorized into genes first appearing in Chordates, Metazoa or all genes. This categorization uses a BPP matrix as presented above (Section 8.5) i.e. phylogenetic matrix binarized by a threshold of BLAST *E*-value. Thus, genes were categorized as Metazoa-specific if no species distant to Metazoa had any gene orthologs based on the BLAST *E*-value criteria, and similarly for Chordata.

### Parasite analysis

A list of parasitic organisms and clades was manually prepared using annotations from The Encyclopedia of Life^67^ and GloBI^68^. Some species’ parasitism was further manually disambiguated. The list is available in Supplementary Table 5. Six major clades containing parasites were selected based on clade size and the number of parasites—Alveolata, Nematoda, Stramenopiles, Microsporidia, Platyhelminthes and Kinetoplastida. These clades contain both parasites and non-parasites (symbiotic or free-living organisms). For the gene enrichment analysis, genes were first filtered to genes conserved in eukaryotes (found in more than 75% of all eukaryotes in the data) and non-conserved in at least one parasitic clade (found in less than 25% of the parasitic organisms in a clade). Then for each gene in each of the selected clades, it was considered found if it occurred in at least 50% of the clade, and otherwise non-found. This yielded a binary occurrence matrix. Intersections and UpSet plots^69^ were produced by the UpSetPlot Python package^70^. GO term enrichment was conducted using the ClusterProfiler package in R^71^.

For parasite exclusion analysis, models were retrained as described above using all clades, excluding clades with any parasites and excluding all parasitic species as described above. Species were excluded by setting values to NA. The models were trained to predict functional interactions in Reactome and were stratified similar to other analyses in the paper. These stratifications are on gene presence in the test set (i.e. Park-Marcotte inspired splits) and for paralogous pairs exclusion. Results are shown in Supplementary Table 6. Clade importance analysis was performed as described above (Section 8.7) and shown in Supplementary Fig. 18. As models are retrained, some differences were noted between these models and the main ones, for example, in slight variations in performance metrics and clade importance attribution.

### Reporting summary

Further information on research design is available in the Nature Research Reporting Summary linked to this article.

## Supplementary information

Supplementary Information

Description of Additional Supplementary Files

Supplementary Data 1

Supplementary Data 2

Supplementary Data 3

Reporting Summary

## Data Availability

Data are available for exploration through the accompanying webserver found at: https://mlpp.cs.huji.ac.il. This webserver allows the user to explore predictions for a single gene, a gene set and functional annotations for a specific gene using the PathScore annotations. Usability is further described in Supplementary Note 2. Gene-pair and gene sets predictions are available exclusively through the webserver described above. PathScore predictions are available through the webserver and are additionally attached as Supplementary Data 3. Phylogenetic profiles, models and other raw data used to produce the analyses presented in this work can be found on Zenodo at: 10.5281/zenodo.5111607. Source data are provided with this paper.

## Source data

Source Data

## Code availability

Code is available via a Github repository at: https://github.com/dst1/MLPP. This includes the pipeline to train the models and produce predictions as well as an example benchmark that reproduces Fig. 1A and Supplementary Fig. 3.
