# Supplementary material for: Co-evolution based machine-learning for predicting functional interactions between human genes
Source: Nat Commun. 2021 Nov 9;12:6454. doi: 10.1038/s41467-021-26792-w (PMC8578642; doi:10.1038/s41467-021-26792-w)
Supplement: Supplementary file 1 — Supplementary Information [file 41467_2021_26792_MOESM1_ESM.pdf]

Supplementary Information

Co-evolution based machine-learning for predicting functional interactions between human genes

Stupp et al.

Table of Contents

Supplementary Note 1 – Comparison to Random Clades 3

Supplementary Note 2 – Description of the Webserver Functionalities 4

Supplementary Tables 5

- Supplementary Table 1 - Clades and Abbreviations 5
- Supplementary Table 2 - Random Clades 7
- Supplementary Table 3 – Performance per Phylogenetic Profile Generation Parameters 9
- Supplementary Table 4 - Model Performance 11
- Supplementary Table 5 - Parasitic Organisms 12
- Supplementary Table 6 – Model Performance Excluding Parasites 16

Supplementary Figures 17

- Supplementary Figure 1 - Clademap 17
- Supplementary Figure 2 – Positive-Unlabeled Learning 19
- Supplementary Figure 3 - Comparing Machine Learning Algorithms 21
- Supplementary Figure 4 – “Young” Genes 24
- Supplementary Figure 5 - Model Performance - Comparing Phylogenetic Profiling Approaches 25
- Supplementary Figure 6 – Reactome Temporal Splits 30
- Supplementary Figure 7 - External Validation – PPI 31
- Supplementary Figure 8 - External Validation – KEGG 33
- Supplementary Figure 9 - External Validation – Complexes (Functional Interaction Model) 34
- Supplementary Figure 10 - External Validation – Complexes (In Complex Model) 36
- Supplementary Figure 11 - Performance Comparison on the Pathway Level 38
- Supplementary Figure 12 - Clusters 40
- Supplementary Figure 13 - Distribution of Pubmed Mentions 41
- Supplementary Figure 14 – PathScore Distribution 42
- Supplementary Figure 15 – PathScore Performance 45
- Supplementary Figure 16 - Clade Importance - Interaction Context Models 47
- Supplementary Figure 17 - UMAP Projection of SHAP Values for Gene Pairs 48
- Supplementary Figure 18 – Clade Importance of Models Excluding Parasites 49

Supplementary References 50

Supplementary Note 1 – Comparison to Random Clades

Local co-evolution tries to identify functionally interacting genes by their phylogenetic profiling similarity in specific clades. However, it remains unclear how sensitive is the model to the phylogenetic tree structure, i.e. how does clade composition affect performance. To investigate this question, we trained the model on randomized species permutations, thus the composition of each clade is random and independent of evolution. We also compared the original model to a model trained with 20% of the species by taking every fifth species and duplicating each of these species five times. This “small” tree model has the covariance for each gene-pair in each clade scaled by a multiple of five from a true subsampling, and thus directly proportional, and provides the same information for a decision-tree based model. This sampling procedure is important, as some clades are as large as a third of the eukaryotic tree. Thus, a random sample of species with the size of a third of the species is likely to be more informative than the “small” (subsampling) tree model for all species. The models were trained in 4-fold cross-validation and performance was measured by the area under the receiver operator characteristics curve (auROC) for all gene-pairs and for gene-pairs excluding paralogs. Results (supp. Table 1) show that the original model, trained on the true permutation and all species, outperformed the random and the “small” models in most comparisons. For some of the labels tested such as Reactome (functional interaction as pathway co-occurrence, see Methods), Reactome complex, and signal transduction, the model shows little to no improvement over the random clades. This may be related to the high clade importance of the bigger clades, particularly Eukaryota and Fungi, in the original model for these labels (Figure 4a,c, Reactome complex not shown). For the other labels, “small” is close in performance to the original model while random permutations are lower in both.

Supplementary Note 2 – Description of the Webserver Functionalities

This manuscript is accompanied by a webserver found at: https://mlpp.cs.huji.ac.il This webserver enables the user to explore the predictions made by the models presented in this manuscript. In addition to predictions, this site features contextual information from various sources to facilitate a deeper understanding of the presented predictions. The webserver enables the user to explore 3 main functionalities which recapitulate analyses performed in this manuscript:

1. Prediction of functional interaction between pairs of human genes – In the tab "Functional Interaction Prediction" the user can select a gene of interest and explore the predicted functional interactions of this gene with all other human genes. The user can select to view predictions for functional interaction as well as each of the other labels described in the paper – complexes, Reactome pathway types, and GO pathway types from the dropdown menu at the top of the page. Only the top 100 predicted genes are displayed, to get the full list the user can click to download a CSV file.
2. Prediction of functional interactions for gene sets – In the tab "Gene Set Interaction Prediction" the user can select a gene set and display the prediction of interactions between them. The webserver generates plots that display the phylogenetic profile of these genes (similar to Figure 3, Supp. Figure 12), and the prediction scores heatmap (similar to Figure 2). The user can choose to explore both established gene sets from Reactome, KEGG, GO and other sources (by selecting known gene set), or select genes to assemble a gene set of interest (by selecting custom gene set). For known gene sets, the webserver displays a description of the gene set when available.
3. Functional annotation of genes using PathScore – In the tab "Functional Annotation ("PathScore")" the user can select a gene and explore its association with the various pathway types considered in this manuscript. This yields information akin to Figure 4C per gene. The tab is further augmented with information about the gene from uniport and GO when available, with links to the source.

Further instructions and examples can be found on the homepage of the webserver.

Supplementary Tables

Supplementary Table 1 - Clades and Abbreviations

Clade	Abbreviation
Aconoidasida	Acono.
Agaricales	Agari.4
Agaricomycetes	Agari.2
Agaricomycetidae	Agari.3
Agaricomycotina	Agari.1
Alveolata	Alveo.
Anopheles	Anoph.1
Archelosauria	Arch.1
Arthropoda	Arthro.
Ascomycota	Asco.
Aspergillus	Asper.2
Asterids	aster.
Basidiomycota	Basido.
Boletales	Bolet.
Bop_Clade	BOP
Brachycera	Barchy.
Capnodiales	Capno.
Catarrhini	Catarr.
Chaetothyriomycetidae	Chaeto.1
Chlorophyta	Chloro.
Chordata	Chord.
Chromadorea	Chroma.
Clavicipitaceae	Clavici.
Conoidasida	Conoi.
Debaryomycetaceae	Debar.
Digenea	Digen.
Diptera	Diptera
Dorylaimia	Doryl.
Dothideomycetes	Dothid.1
Dothideomycetidae	Dothid.2
Ecdysozoa	Ecdys.
Ephydroidea	Ephyd.
Euarchontoglires	Euarcho.
Eudicotyledons	eudicot.
Euglenozoa	Euglen.

Clade (cont.)	Abbreviation (cont.)
Glomerellales	Glomer.1
Helotiales	Helo.
Hymenoptera	Hymen.
Hypocreaceae	Hypoc.3
Hypocreales	Hypoc.2
Hypocreomycetidae	Hypoc.1
Lamiids	lamiids
Laurasiatheria	Lauras.
Leotiomycetes	Leo.
Liliopsida	Lilio.
Malvids	malvids
Mammalia	Mammalia
Metazoa	Metazoa
Microsporidia	Micro.
Mucoromycota	Mucor.
Mycosphaerellaceae	Mycos.
Nectriaceae	Nectr.
Nematocera	Nematocera
Nematoda	Nematoda
Neopterygii	Neop.
Onygenales	Onyg.
Oomycetes	Oomyc.
Penicillium	Penicill.
Pezizomycotina	Pezizo
Plasmodiidae	Plasm.2
Platyhelminthes	Platy.
Pleosporineae	Pleos.3
Pleosporomycetidae	Pleos.2
Poales	Poales
Polyporales	Polyp.
Primates	Primates
Pucciniomycotina	Pucci.
Rhabditina	Rhabd.2
Rosids	rosids
Saccharomycetaceae	Sacch.4

Eukaryota	Eukaryota	Saccharomycotina	Sacch.3
Eurotiales	Euro.3	Sordariomycetes	Sord.1
Eurotiomycetes	Euro.1	Sordariomycetidae	Sord.2
Eurotiomycetidae	Euro.2	Spirurina	Spirur.
Fabids	fabids	Stramenopiles	Stram.
Formicidae	Formi.2	Taphrinomycotina	Taphir.
Fungi	Fungi	Tremellomycetes	Tremel.1
Fungi_Incertae_Sedis	Fungi.IS	Ustilaginomycotina	Ustil.
Glires	Glires	Viridiplantae	Virid.

Clades and Abbreviations - Clades and abbreviations used in this paper. Clades marked in blue are the 49 clades used for generating the models (see Methods).

Supplementary Table 2 - Random Clades

		Unfiltered					Paralogs filtered				
**Target**	**Clades**	**Train**	**Test**	**C1**	**C2**	**C3**	**Train**	**Test**	**C1**	**C2**	**C3**
KEGG	orig	0.842	0.800	0.838	0.792	0.783	0.839	0.795	0.835	0.789	0.762
KEGG	small	0.842	0.805	0.837	0.797	0.793	0.839	0.799	0.835	0.794	0.771
KEGG	rand_1	0.806	0.756	0.797	0.752	0.706	0.807	0.757	0.798	0.751	0.712
KEGG	rand_2	0.817	0.766	0.812	0.760	0.720	0.818	0.767	0.813	0.760	0.720
KEGG	rand_3	0.817	0.764	0.812	0.757	0.717	0.817	0.763	0.811	0.756	0.714
KEGG	rand_4	0.805	0.757	0.799	0.753	0.708	0.807	0.760	0.801	0.753	0.719
Reactome	orig	0.741	0.724	0.737	0.713	0.736	0.733	0.713	0.729	0.708	0.713
Reactome	small	0.733	0.717	0.729	0.706	0.730	0.726	0.708	0.722	0.703	0.710
Reactome	rand_1	0.717	0.720	0.711	0.713	0.734	0.715	0.717	0.709	0.714	0.727
Reactome	rand_2	0.725	0.722	0.720	0.714	0.732	0.723	0.718	0.717	0.714	0.726
Reactome	rand_3	0.728	0.717	0.724	0.708	0.723	0.725	0.711	0.720	0.707	0.708
Reactome	rand_4	0.719	0.719	0.712	0.711	0.732	0.717	0.715	0.710	0.712	0.724
Complexes Reactome	orig	0.787	0.771	0.777	0.760	0.787	0.782	0.766	0.772	0.761	0.779
Complexes Reactome	small	0.776	0.764	0.764	0.756	0.780	0.772	0.761	0.761	0.756	0.776
Complexes Reactome	rand_1	0.753	0.765	0.742	0.763	0.781	0.754	0.767	0.743	0.766	0.796
Complexes Reactome	rand_2	0.764	0.771	0.754	0.767	0.788	0.765	0.772	0.754	0.771	0.800
Complexes Reactome	rand_3	0.767	0.759	0.757	0.755	0.767	0.768	0.760	0.758	0.758	0.772
Complexes Reactome	rand_4	0.756	0.760	0.745	0.756	0.774	0.757	0.761	0.745	0.759	0.782
Developmental Biology	orig	0.896	0.873	0.868	0.864	0.883	0.896	0.865	0.866	0.861	0.857
Developmental Biology	small	0.898	0.871	0.867	0.862	0.873	0.897	0.864	0.862	0.860	0.849
Developmental Biology	rand_1	0.885	0.853	0.856	0.843	0.850	0.883	0.849	0.853	0.845	0.829
Developmental Biology	rand_2	0.889	0.858	0.862	0.851	0.856	0.887	0.854	0.859	0.850	0.842
Developmental Biology	rand_3	0.891	0.856	0.859	0.847	0.855	0.889	0.849	0.856	0.845	0.830
Developmental Biology	rand_4	0.889	0.858	0.865	0.847	0.854	0.887	0.853	0.861	0.846	0.834
Metabolism	orig	0.837	0.832	0.825	0.821	0.864	0.834	0.820	0.820	0.817	0.819
Metabolism	small	0.839	0.840	0.826	0.831	0.881	0.835	0.829	0.821	0.827	0.839
Metabolism	rand_1	0.801	0.787	0.787	0.780	0.789	0.801	0.784	0.786	0.781	0.772
Metabolism	rand_2	0.807	0.789	0.793	0.779	0.800	0.807	0.783	0.793	0.779	0.770
Metabolism	rand_3	0.812	0.791	0.797	0.778	0.801	0.811	0.783	0.797	0.777	0.765
Metabolism	rand_4	0.801	0.782	0.784	0.774	0.784	0.802	0.778	0.784	0.774	0.765
DNA Repair	orig	0.929	0.901	0.907	0.897	0.916	0.929	0.899	0.907	0.898	0.888
DNA Repair	small	0.922	0.895	0.898	0.890	0.918	0.924	0.893	0.898	0.890	0.885
DNA Repair	rand_1	0.906	0.863	0.866	0.854	0.897	0.906	0.857	0.866	0.849	0.847
DNA Repair	rand_2	0.916	0.876	0.882	0.867	0.901	0.916	0.871	0.881	0.865	0.855
DNA Repair	rand_3	0.897	0.848	0.857	0.836	0.878	0.898	0.845	0.856	0.838	0.830
DNA Repair	rand_4	0.903	0.854	0.863	0.846	0.878	0.904	0.852	0.865	0.846	0.840

Signal Transduction	orig	0.794	0.780	0.775	0.770	0.793	0.789	0.770	0.768	0.765	0.779
Signal Transduction	small	0.795	0.781	0.778	0.772	0.789	0.791	0.775	0.773	0.770	0.782
Signal Transduction	rand_1	0.794	0.773	0.782	0.769	0.763	0.790	0.772	0.776	0.767	0.771
Signal Transduction	rand_2	0.803	0.776	0.789	0.772	0.767	0.799	0.775	0.784	0.770	0.776
Signal Transduction	rand_3	0.798	0.770	0.786	0.759	0.764	0.793	0.762	0.780	0.756	0.749
Signal Transduction	rand_4	0.800	0.775	0.787	0.771	0.766	0.796	0.774	0.782	0.769	0.775

Random Clades – Model performance as measured by the mean auROC across four cross-validations for Functional Interaction models trained on KEGG or Reactome, or Interaction Context models for Complexes, Developmental Biology, Metabolism, DNA Repair or Signal Transduction compared for using all clades (“orig”), five random permutations (“rand_1”-“rand_4”) or a subsample of 20% of species (“small”). A blue gradient ranks the best performing models from best to worst (blue to white, respectively). C1, C2, and C3 are stratifications for pairwise prediction for pairs of genes appearing both in the training set, one appearing in the training set, and not appearing in the training set, respectively (see Methods, based on [1]). Model evaluation was conducted with pairs of paralogs (unfiltered) or without (Paralogs filtered). More details of the comparison are given in the Supp. Text.

Supplementary Table 3 – Performance per Phylogenetic Profile Generation Parameters

Paralog filtering	Method Type	PP Matrix	Distance	Train AUC mean	Train AUC std	Train pAUC 0.1 mean	Train pAUC 0.1 std	Train AP mean	Train AP std	Test AUC mean	Test AUC std	Test pAUC 0.1 mean	Test pAUC 0.1 std	Test AP mean	Test AP std
Paralogs Unfiltered	All Eukaryotes	BLAST E-value	Hamming	0.640	±0.01	0.562	±0.00	0.640	±0.00	0.644	±0.00	0.578	±0.00	0.674	±0.00
		BLAST E-value	Jaccard	0.689	±0.01	0.568	±0.00	0.672	±0.00	0.675	±0.00	0.575	±0.00	0.688	±0.00
		NPP	Pearson	0.608	±0.01	0.550	±0.00	0.620	±0.01	0.599	±0.00	0.568	±0.00	0.651	±0.00
		BLAST E-value	Cov.	0.481	±0.01	0.524	±0.00	0.517	±0.01	0.460	±0.00	0.525	±0.00	0.538	±0.00
		Len. norm. (T=bs40)	Cov.	0.571	±0.01	0.527	±0.00	0.564	±0.01	0.573	±0.01	0.537	±0.00	0.600	±0.01
		Len. norm. (T=bs60)	Cov.	0.571	±0.01	0.526	±0.00	0.563	±0.01	0.570	±0.01	0.535	±0.00	0.595	±0.01
		Len. norm. (T=bs100)	Cov.	0.578	±0.01	0.526	±0.00	0.566	±0.01	0.571	±0.01	0.533	±0.00	0.593	±0.01
	MLPP	BLAST E-value	Hamming	0.744	±0.00	0.583	±0.00	0.716	±0.01	0.706	±0.00	0.578	±0.00	0.707	±0.00
		BLAST E-value	Jaccard	0.756	±0.00	0.588	±0.00	0.726	±0.01	0.713	±0.00	0.573	±0.00	0.707	±0.00
		NPP	Pearson	0.711	±0.01	0.598	±0.00	0.717	±0.00	0.697	±0.00	0.601	±0.00	0.726	±0.00
		BLAST E-value	Cov.	0.707	±0.00	0.582	±0.00	0.699	±0.01	0.674	±0.01	0.558	±0.00	0.678	±0.01
		Len. norm. (T=bs40)	Cov.	0.746	±0.00	0.608	±0.01	0.741	±0.01	0.727	±0.00	0.595	±0.00	0.737	±0.00
		Len. norm. (T=bs60)	Cov.	0.742	±0.00	0.610	±0.01	0.741	±0.01	0.725	±0.00	0.593	±0.00	0.734	±0.00
		Len. norm. (T=bs100)	Cov.	0.735	±0.00	0.609	±0.00	0.735	±0.00	0.716	±0.00	0.588	±0.00	0.725	±0.00
Paralogs Filtered	All Eukaryotes	BLAST E-value	Hamming	0.620	±0.01	0.546	±0.00	0.596	±0.00	0.609	±0.00	0.550	±0.00	0.604	±0.00
		BLAST E-value	Jaccard	0.672	±0.01	0.556	±0.00	0.634	±0.00	0.644	±0.00	0.552	±0.00	0.626	±0.00
		NPP	Pearson	0.586	±0.01	0.529	±0.00	0.563	±0.01	0.561	±0.00	0.533	±0.00	0.564	±0.01
		BLAST E-value	Cov.	0.463	±0.01	0.511	±0.00	0.471	±0.01	0.431	±0.00	0.504	±0.00	0.462	±0.00
		Len. norm. (T=bs40)	Cov.	0.564	±0.01	0.524	±0.00	0.539	±0.01	0.558	±0.01	0.530	±0.00	0.556	±0.01
		Len. norm. (T=bs60)	Cov.	0.563	±0.01	0.523	±0.00	0.538	±0.01	0.556	±0.01	0.528	±0.00	0.551	±0.01
		Len. norm. (T=bs100)	Cov.	0.571	±0.01	0.523	±0.00	0.541	±0.01	0.557	±0.01	0.527	±0.00	0.550	±0.01
	MLPP	BLAST E-value	Hamming	0.729	±0.00	0.565	±0.00	0.677	±0.01	0.678	±0.00	0.550	±0.00	0.642	±0.01
		BLAST E-value	Jaccard	0.743	±0.00	0.573	±0.00	0.693	±0.01	0.687	±0.00	0.554	±0.00	0.651	±0.00
		NPP	Pearson	0.694	±0.01	0.577	±0.00	0.673	±0.00	0.668	±0.00	0.568	±0.00	0.659	±0.00
		BLAST E-value	Cov.	0.691	±0.00	0.563	±0.00	0.657	±0.01	0.648	±0.01	0.531	±0.00	0.611	±0.01
		Len. norm. (T=bs40)	Cov.	0.738	±0.00	0.601	±0.01	0.720	±0.01	0.716	±0.00	0.587	±0.00	0.703	±0.00
		Len. norm. (T=bs60)	Cov.	0.734	±0.00	0.603	±0.01	0.719	±0.01	0.712	±0.00	0.585	±0.00	0.700	±0.00
		Len. norm. (T=bs100)	Cov.	0.727	±0.00	0.602	±0.00	0.712	±0.00	0.702	±0.00	0.580	±0.00	0.690	±0.00

Paralog filtering	Method Type	PP Matrix	Distance	C1						C2						C3					
				AUC		pAUC 0.1		AP		AUC		pAUC 0.1		AP		AUC		pAUC 0.1		AP	
				mean	std	mean	std	mean	std	mean	std	mean	std	mean	std	mean	std	mean	std	mean	std
Paralogs Unfiltered	All Eukaryotes	BLAST E-value	Hamming	0.641	±0.01	0.563	±0.00	0.654	±0.01	0.622	±0.00	0.559	±0.00	0.626	±0.01	0.698	±0.01	0.625	±0.01	0.779	±0.00
		BLAST E-value	Jaccard	0.690	±0.01	0.567	±0.00	0.685	±0.01	0.656	±0.00	0.559	±0.00	0.644	±0.01	0.709	±0.01	0.614	±0.01	0.781	±0.00
		NPP	Pearson	0.609	±0.01	0.553	±0.00	0.634	±0.01	0.574	±0.00	0.547	±0.00	0.597	±0.01	0.657	±0.01	0.625	±0.01	0.771	±0.01
		BLAST E-value	Cov.	0.483	±0.01	0.525	±0.00	0.534	±0.01	0.444	±0.00	0.515	±0.00	0.494	±0.00	0.489	±0.01	0.547	±0.01	0.649	±0.01
		Len. norm. (T=bs40)	Cov.	0.574	±0.02	0.528	±0.00	0.580	±0.01	0.565	±0.01	0.534	±0.00	0.569	±0.01	0.588	±0.01	0.552	±0.01	0.687	±0.02
		Len. norm. (T=bs60)	Cov.	0.574	±0.01	0.526	±0.00	0.578	±0.01	0.563	±0.01	0.532	±0.00	0.565	±0.01	0.585	±0.01	0.548	±0.00	0.681	±0.02
		Len. norm. (T=bs100)	Cov.	0.581	±0.01	0.526	±0.00	0.582	±0.01	0.564	±0.01	0.530	±0.00	0.563	±0.01	0.582	±0.01	0.545	±0.00	0.678	±0.01
	MLPP	BLAST E-value	Hamming	0.733	±0.01	0.579	±0.00	0.718	±0.01	0.693	±0.01	0.561	±0.00	0.667	±0.01	0.721	±0.01	0.617	±0.00	0.787	±0.01
		BLAST E-value	Jaccard	0.744	±0.01	0.582	±0.00	0.727	±0.01	0.701	±0.00	0.561	±0.00	0.671	±0.00	0.717	±0.01	0.589	±0.01	0.773	±0.01
		NPP	Pearson	0.686	±0.01	0.586	±0.00	0.704	±0.01	0.678	±0.00	0.581	±0.00	0.684	±0.00	0.750	±0.01	0.649	±0.00	0.821	±0.01
		BLAST E-value	Cov.	0.691	±0.01	0.569	±0.00	0.691	±0.00	0.663	±0.01	0.546	±0.00	0.643	±0.01	0.685	±0.01	0.578	±0.01	0.750	±0.01
		Len. norm. (T=bs40)	Cov.	0.730	±0.01	0.594	±0.00	0.732	±0.01	0.719	±0.00	0.588	±0.00	0.710	±0.01	0.744	±0.01	0.613	±0.01	0.804	±0.01
		Len. norm. (T=bs60)	Cov.	0.729	±0.01	0.595	±0.01	0.732	±0.01	0.716	±0.00	0.586	±0.00	0.706	±0.00	0.745	±0.01	0.608	±0.01	0.802	±0.01
		Len. norm. (T=bs100)	Cov.	0.725	±0.01	0.595	±0.01	0.728	±0.01	0.708	±0.00	0.580	±0.00	0.699	±0.00	0.725	±0.01	0.598	±0.01	0.787	±0.01
Paralogs Filtered	All Eukaryotes	BLAST E-value	Hamming	0.621	±0.01	0.547	±0.00	0.610	±0.01	0.605	±0.00	0.547	±0.00	0.591	±0.01	0.613	±0.01	0.565	±0.00	0.642	±0.01
		BLAST E-value	Jaccard	0.673	±0.01	0.554	±0.01	0.646	±0.01	0.642	±0.00	0.549	±0.00	0.614	±0.01	0.633	±0.01	0.567	±0.00	0.653	±0.01
		NPP	Pearson	0.587	±0.01	0.531	±0.00	0.577	±0.01	0.556	±0.00	0.531	±0.00	0.551	±0.01	0.561	±0.01	0.541	±0.01	0.598	±0.02
		BLAST E-value	Cov.	0.465	±0.01	0.512	±0.00	0.487	±0.01	0.428	±0.01	0.504	±0.00	0.452	±0.01	0.414	±0.01	0.500	±0.00	0.482	±0.02
		Len. norm. (T=bs40)	Cov.	0.567	±0.01	0.525	±0.00	0.555	±0.01	0.557	±0.01	0.530	±0.00	0.548	±0.01	0.554	±0.01	0.534	±0.01	0.583	±0.02
		Len. norm. (T=bs60)	Cov.	0.567	±0.01	0.523	±0.00	0.553	±0.01	0.555	±0.01	0.528	±0.00	0.544	±0.01	0.550	±0.01	0.530	±0.01	0.576	±0.02
		Len. norm. (T=bs100)	Cov.	0.574	±0.01	0.523	±0.00	0.557	±0.01	0.556	±0.01	0.527	±0.00	0.542	±0.01	0.546	±0.01	0.528	±0.00	0.570	±0.02
	MLPP	BLAST E-value	Hamming	0.717	±0.01	0.561	±0.01	0.678	±0.01	0.679	±0.01	0.548	±0.00	0.634	±0.01	0.645	±0.01	0.557	±0.00	0.652	±0.01
		BLAST E-value	Jaccard	0.730	±0.01	0.567	±0.01	0.692	±0.01	0.689	±0.01	0.551	±0.00	0.643	±0.01	0.652	±0.01	0.561	±0.00	0.658	±0.01
		NPP	Pearson	0.667	±0.01	0.563	±0.00	0.656	±0.01	0.664	±0.00	0.565	±0.00	0.645	±0.01	0.683	±0.01	0.583	±0.00	0.701	±0.01
		BLAST E-value	Cov.	0.674	±0.01	0.550	±0.00	0.646	±0.00	0.649	±0.01	0.531	±0.00	0.603	±0.01	0.625	±0.01	0.517	±0.00	0.609	±0.02
		Len. norm. (T=bs40)	Cov.	0.722	±0.01	0.588	±0.00	0.709	±0.01	0.713	±0.00	0.585	±0.00	0.693	±0.01	0.723	±0.01	0.593	±0.01	0.731	±0.01
		Len. norm. (T=bs60)	Cov.	0.720	±0.01	0.589	±0.01	0.709	±0.01	0.709	±0.00	0.582	±0.00	0.689	±0.01	0.721	±0.01	0.590	±0.01	0.727	±0.01
		Len. norm. (T=bs100)	Cov.	0.715	±0.01	0.588	±0.00	0.705	±0.01	0.701	±0.00	0.577	±0.00	0.681	±0.01	0.698	±0.01	0.585	±0.01	0.711	±0.01

Phylogenetic Profiling Generation Performance – blast bitscore (40, 60, 100) and E-value (1e-3) thresholds as well as various normalization (NPP, length normalization, binarization – "BLAST E-value") and distance metrics ("Hamming", "Jaccard", "Pearson" – Pearson correlation, and "Cov." - covariance) are compared based on performance in predicting functional interactions in Reactome. Comparisons are conducted with the complete machine learning pipeline (MLPP) or using just one clade – all Eukaryotes. Comparisons are stratified based on including pairs of paralogous genes ("Paralogs Unfiltered") or filtering them ('Paralogs Filtered"). C1, C2, and C3 are stratifications for pairwise prediction with both, one or none of the genes in a pair appear in the training set, respectively (see Methods, based on [1]). Performance is measured as auROC, partial ROC AUC at FPR 0.1 (pAUC) and average precision (AP), and presented as the mean ± standard deviation across five cross validation folds.

Supplementary Table 4 - Model Performance

		Unf lt					Paralogs f ltered			
**Metric**	**Split**	**MLPP**	**NPP**	**SVDp**	**PPP**	**BPP**	**MLPP**	**NPP**	**SVDp**	**PPP**
AUC	Train	0.759	0.617	0.617	0.626	0.639	0.747	0.593	0.594	0.599
	Test	0.731	0.596	0.592	0.632	0.643	0.717	0.559	0.554	0.594
	C1	0.744	0.615	0.618	0.626	0.640	0.732	0.592	0.597	0.600
	C2	0.720	0.570	0.563	0.605	0.619	0.713	0.554	0.548	0.589
	C3	0.738	0.646	0.637	0.690	0.694	0.715	0.552	0.541	0.600
pAUC FPR 0.1	Train	0.626	0.554	0.544	0.564	0.561	0.614	0.531	0.528	0.536
	Test	0.599	0.564	0.554	0.572	0.575	0.588	0.530	0.526	0.535
	C1	0.610	0.552	0.545	0.563	0.561	0.599	0.529	0.528	0.536
	C2	0.591	0.541	0.533	0.547	0.552	0.586	0.528	0.524	0.531
	C3	0.610	0.619	0.602	0.625	0.619	0.588	0.536	0.529	0.543
AP	Train	0.729	0.586	0.571	0.605	0.599	0.699	0.522	0.513	0.536
	Test	0.756	0.664	0.651	0.689	0.688	0.722	0.581	0.572	0.605
	C1	0.753	0.637	0.629	0.657	0.653	0.724	0.578	0.576	0.595
	C2	0.723	0.601	0.584	0.627	0.630	0.706	0.563	0.553	0.584
	C3	0.830	0.796	0.784	0.818	0.807	0.765	0.641	0.629	0.672

Model Performance – Functional Interaction model performance as measured by the mean across five-fold cross-validation. Phylogenetic profiling algorithms were compared on the basis of predicting gene-pair functional interaction (co-occurrence in a Reactome pathway). C1, C2, and C3 are stratifications for pairwise prediction for pairs of genes as in Supp. Table 3. Model evaluation is conducted with pairs of paralogs (Unfiltered) or without (Paralogs Filtered). MLPP - machine learning phylogenetic profiling (the method presented in this paper), NPP - normalized phylogenetic profiling, SVDp – SVD-Phy, PPP - PrePhyloPro, BPP - Hamming distance on binarized phylogenetic profiles, AUC - area under the ROC curve, pAUC - partial AUC at false positive rate (FPR) of 0.1. AP – average precision.

Supplementary Table 5 - Parasitic Organisms

NCBI taxid	Taxonomical Name	Clade	Ref. Clade
241478	*Soboliphyme baturini*	Nematoda	Metazoa
70415	*Trichuris muris*	Nematoda	Metazoa
36087	*Trichuris trichiura*	Nematoda	Metazoa
68888	*Trichuris suis*	Nematoda	Metazoa
6336	*Trichinella nelsoni*	Nematoda	Metazoa
45882	*Trichinella britovi*	Nematoda	Metazoa
268475	*Trichinella zimbabwensis*	Nematoda	Metazoa
268474	*Trichinella papuae*	Nematoda	Metazoa
144512	*Trichinella murrelli*	Nematoda	Metazoa
990121	*Trichinella patagoniensis*	Nematoda	Metazoa
6335	*Trichinella nativa*	Nematoda	Metazoa
6337	*Trichinella pseudospiralis*	Nematoda	Metazoa
6334	*Trichinella spiralis*	Nematoda	Metazoa
181606	*Trichinella sp. T9*	Nematoda	Metazoa
334426	*Angiostrongylus costaricensis*	Nematoda	Metazoa
6313	*Angiostrongylus cantonensis*	Nematoda	Metazoa
27835	*Nippostrongylus brasiliensis*	Nematoda	Metazoa
375939	*Heligmosomoides polygyrus bakeri*	Nematoda	Metazoa
29172	*Dictyocaulus viviparus*	Nematoda	Metazoa
45464	*Teladorsagia circumcincta*	Nematoda	Metazoa
6290	*Haemonchus placei*	Nematoda	Metazoa
6289	*Haemonchus contortus*	Nematoda	Metazoa
318479	*Dracunculus medinensis*	Nematoda	Metazoa
451379	*Syphacia muris*	Nematoda	Metazoa
51028	*Enterobius vermicularis*	Nematoda	Metazoa
6269	*Anisakis simplex*	Nematoda	Metazoa
6252	*Ascaris lumbricoides*	Nematoda	Metazoa
6265	*Toxocara canis*	Nematoda	Metazoa
103827	*Thelazia callipaeda*	Nematoda	Metazoa
637853	*Gongylonema pulchrum*	Nematoda	Metazoa
6293	*Wuchereria bancrofti*	Nematoda	Metazoa
7209	*Loa loa*	Nematoda	Metazoa
6279	*Brugia malayi*	Nematoda	Metazoa
42155	*Brugia timori*	Nematoda	Metazoa
6280	*Brugia pahangi*	Nematoda	Metazoa
6282	*Onchocerca volvulus*	Nematoda	Metazoa
387005	*Onchocerca flexuosa*	Nematoda	Metazoa
42157	*Onchocerca ochengi*	Nematoda	Metazoa
6326	*Bursaphelenchus xylophilus*	Nematoda	Metazoa
36090	*Globodera pallida*	Nematoda	Metazoa
6305	*Meloidogyne hapla*	Nematoda	Metazoa
37863	*Steinernema glaseri*	Nematoda	Metazoa
131310	*Parastrongyloides trichosuri*	Nematoda	Metazoa
75913	*Strongyloides venezuelensis*	Nematoda	Metazoa
34506	*Strongyloides ratti*	Nematoda	Metazoa
174720	*Strongyloides papillosus*	Nematoda	Metazoa
61180	*Oesophagostomum dentatum*	Nematoda	Metazoa
37862	*Heterorhabditis bacteriophora*	Nematoda	Metazoa
51022	*Ancylostoma duodenale*	Nematoda	Metazoa
53326	*Ancylostoma ceylanicum*	Nematoda	Metazoa
51031	*Necator americanus*	Nematoda	Metazoa
418985	*Tropilaelaps mercedesae*	Arthropoda	

6945	*Ixodes scapularis*	Arthropoda	
52283	*Sarcoptes scabiei*	Arthropoda	
121224	*Pediculus humanus subsp. corporis*	Arthropoda	
7425	*Nasonia vitripennis*	Arthropoda	
70667	*Schistocephalus solidus*	Platyhelminthes	Metazoa
53468	*Mesocestoides corti*	Platyhelminthes	Metazoa
6216	*Hymenolepis diminuta*	Platyhelminthes	Metazoa
102285	*Hymenolepis nana*	Platyhelminthes	Metazoa
85433	*Hymenolepis microstoma*	Platyhelminthes	Metazoa
60517	*Taenia asiatica*	Platyhelminthes	Metazoa
6205	*Hydatigena taeniaeformis*	Platyhelminthes	Metazoa
6211	*Echinococcus multilocularis*	Platyhelminthes	Metazoa
6210	*Echinococcus granulosus*	Platyhelminthes	Metazoa
6192	*Fasciola hepatica*	Platyhelminthes	Metazoa
27848	*Echinostoma caproni*	Platyhelminthes	Metazoa
6198	*Opisthorchis viverrini*	Platyhelminthes	Metazoa
79923	*Clonorchis sinensis*	Platyhelminthes	Metazoa
157069	*Trichobilharzia regenti*	Platyhelminthes	Metazoa
6185	*Schistosoma haematobium*	Platyhelminthes	Metazoa
48269	*Schistosoma margrebowiei*	Platyhelminthes	Metazoa
6188	*Schistosoma rodhaini*	Platyhelminthes	Metazoa
6183	*Schistosoma mansoni*	Platyhelminthes	Metazoa
6186	*Schistosoma curassoni*	Platyhelminthes	Metazoa
31246	*Schistosoma mattheei*	Platyhelminthes	Metazoa
669202	*Thelohanellus kitauei*	Other Metazoa	
478820	*Blastocystis sp. subtype 1*	Stramenopiles	Eukaryota
12968	*Blastocystis hominis*	Stramenopiles	Eukaryota
65357	*Albugo candida*	Stramenopiles	Eukaryota
114742	*Pythium insidiosum*	Stramenopiles	Eukaryota
4781	*Plasmopara halstedii*	Stramenopiles	Eukaryota
559515	*Hyaloperonospora arabidopsidis*	Stramenopiles	Eukaryota
29920	*Phytophthora cactorum*	Stramenopiles	Eukaryota
403677	*Phytophthora infestans (strain T30-4)*	Stramenopiles	Eukaryota
4790	*Phytophthora nicotianae*	Stramenopiles	Eukaryota
164328	*Phytophthora ramorum*	Stramenopiles	Eukaryota
4795	*Phytophthora megakarya*	Stramenopiles	Eukaryota
611791	*Phytophthora palmivora var. palmivora*	Stramenopiles	Eukaryota
1317063	*Phytophthora parasitica CJ01A1*	Stramenopiles	Eukaryota
423536	*Perkinsus marinus*	Alveolata	Eukaryota
266149	*Pseudocohnilembus persalinus*	Alveolata	Eukaryota
110365	*Gregarina niphandrodes*	Alveolata	Eukaryota
353152	*Cryptosporidium parvum (strain Iowa II)*	Alveolata	Eukaryota
857276	*Cryptosporidium ubiquitum*	Alveolata	Eukaryota
441375	*Cryptosporidium muris (strain RN66)*	Alveolata	Eukaryota
483139	*Cystoisospora suis*	Alveolata	Eukaryota
94643	*Besnoitia besnoiti*	Alveolata	Eukaryota
99158	*Hammondia hammondi*	Alveolata	Eukaryota
432359	*Toxoplasma gondii (strain ATCC 50861 / VEG)*	Alveolata	Eukaryota
572307	*Neospora caninum (strain Liverpool)*	Alveolata	Eukaryota
88456	*Cyclospora cayetanensis*	Alveolata	Eukaryota
51316	*Eimeria praecox*	Alveolata	Eukaryota
5802	*Eimeria tenella*	Alveolata	Eukaryota
5801	*Eimeria acervulina*	Alveolata	Eukaryota
44415	*Eimeria mitis*	Alveolata	Eukaryota
51314	*Eimeria brunetti*	Alveolata	Eukaryota

5804	*Eimeria maxima*	Alveolata	Eukaryota
51315	*Eimeria necatrix*	Alveolata	Eukaryota
1537102	*Theileria equi strain WA*	Alveolata	Eukaryota
869250	*Theileria orientalis strain Shintoku*	Alveolata	Eukaryota
5875	*Theileria parva*	Alveolata	Eukaryota
5874	*Theileria annulata*	Alveolata	Eukaryota
5865	*Babesia bovis*	Alveolata	Eukaryota
462227	*Babesia sp. Xinjiang*	Alveolata	Eukaryota
1133968	*Babesia microti (strain RI)*	Alveolata	Eukaryota
5866	*Babesia bigemina*	Alveolata	Eukaryota
189622	*Babesia ovata*	Alveolata	Eukaryota
208452	*Plasmodium coatneyi*	Alveolata	Eukaryota
85471	*Plasmodium relictum*	Alveolata	Eukaryota
1036723	*Plasmodium falciparum Vietnam Oak-Knoll*	Alveolata	Eukaryota
647221	*Plasmodium gaboni*	Alveolata	Eukaryota
5821	*Plasmodium berghei*	Alveolata	Eukaryota
31271	*Plasmodium chabaudi chabaudi*	Alveolata	Eukaryota
73239	*Plasmodium yoelii yoelii*	Alveolata	Eukaryota
5851	*Plasmodium knowlesi (strain H)*	Alveolata	Eukaryota
5858	*Plasmodium malariae*	Alveolata	Eukaryota
77519	*Plasmodium gonderi*	Alveolata	Eukaryota
126793	*Plasmodium vivax (strain Salvador I)*	Alveolata	Eukaryota
1120755	*Plasmodium cynomolgi strain B*	Alveolata	Eukaryota
864141	*Plasmodium ovale curtisi*	Alveolata	Eukaryota
1237626	*Plasmodium inui San Antonio 1*	Alveolata	Eukaryota
5857	*Plasmodium fragile*	Alveolata	Eukaryota
1291522	*Helicosporidium sp. ATCC 50920*	Viridiplantae	
948595	*Vavraia culicis (isolate floridensis)*	Microsporidia	Fungi
1240240	*Anncaliia algerae PRA109*	Microsporidia	Fungi
1485682	*Mitosporidium daphniae*	Microsporidia	Fungi
1003232	*Edhazardia aedis (strain USNM 41457)*	Microsporidia	Fungi
1081669	*Hepatospora eriocheir*	Microsporidia	Fungi
40302	*Nosema ceranae*	Microsporidia	Fungi
578461	*Nosema bombycis (strain CQ1 / CVCC 102059)*	Microsporidia	Fungi
907965	*Encephalitozoon hellem (strain ATCC 50504)*	Microsporidia	Fungi
284813	*Encephalitozoon cuniculi (strain GB-M1)*	Microsporidia	Fungi
1178016	*Encephalitozoon romaleae*	Microsporidia	Fungi
876142	*Encephalitozoon intestinalis*	Microsporidia	Fungi
1408658	*Pneumocystis carinii (strain B80)*	Ascomycota	
1069680	*Pneumocystis murina (strain B123)*	Ascomycota	
1408657	*Pneumocystis jirovecii (strain RU7)*	Ascomycota	
59799	*Angomonas deanei*	Kinetoplastida	Eukaryota
28005	*Strigomonas culicis*	Kinetoplastida	Eukaryota
134006	*Phytomonas sp. isolate EM1*	Kinetoplastida	Eukaryota
157538	*Leptomonas pyrrhocoris*	Kinetoplastida	Eukaryota
5684	*Leptomonas seymouri*	Kinetoplastida	Eukaryota
5660	*Leishmania braziliensis*	Kinetoplastida	Eukaryota
929439	*Leishmania mexicana*	Kinetoplastida	Eukaryota
5664	*Leishmania major*	Kinetoplastida	Eukaryota
5671	*Leishmania infantum*	Kinetoplastida	Eukaryota
429131	*Trypanosoma rangeli SC58*	Kinetoplastida	Eukaryota
67003	*Trypanosoma theileri*	Kinetoplastida	Eukaryota
1416333	*Trypanosoma cruzi Dm28c*	Kinetoplastida	Eukaryota
1055687	*Trypanosoma vivax (strain Y486)*	Kinetoplastida	Eukaryota
185431	*Trypanosoma brucei brucei*	Kinetoplastida	Eukaryota

1068625	*Trypanosoma congolense (strain IL3000)*	Kinetoplastida	Eukaryota
5722	*Trichomonas vaginalis*	Other Eukaryotes	
1144522	*Tritrichomonas foetus*	Other Eukaryotes	
348837	*Spironucleus salmonicida*	Other Eukaryotes	
184922	*Giardia intestinalis*	Other Eukaryotes	
598745	*Giardia intestinalis*	Other Eukaryotes	
370354	*Entamoeba dispar*	Amoebozoa	
370355	*Entamoeba invadens IP1*	Amoebozoa	
5759	*Entamoeba histolytica*	Amoebozoa	

Parasitic organisms - List of all parasitic organisms included in the analysis. Designation as parasitic was decided by manual curation based on several databases (see Methods). Clade denotes the clade displayed in Figure 7 and are arbitrarily chosen from the lineage of the organism for visualization purposes. The reference clade is a parent clade used for comparison of percent conservation (Figure 7b,c, see Methods).

Supplementary Table 6 – Model Performance Excluding Parasites

			BPP		NPP		MLPP		
			All species	No para-sites	All species	No para-sites	All species	No para-sites	No parasitic clades
Paralogs	Metric	Split	mean std	mean std	mean std	mean std	mean std	mean std	mean std
Unfilt.	AUC	train	0.64±0.00	0.64±0.00	0.61±0.01	0.60±0.01	0.74±0.01	0.73±0.01	0.73±0.01
		test	0.64±0.00	0.64±0.00	0.60±0.00	0.59±0.00	0.72±0.00	0.72±0.00	0.71±0.00
		C1	0.64±0.01	0.64±0.01	0.61±0.01	0.60±0.01	0.73±0.01	0.73±0.01	0.72±0.01
		C2	0.62±0.00	0.62±0.00	0.57±0.00	0.57±0.00	0.71±0.00	0.71±0.00	0.70±0.00
		C3	0.69±0.01	0.69±0.01	0.65±0.01	0.65±0.01	0.73±0.01	0.72±0.01	0.72±0.01
	pAUC (FPR0.1)	train	0.56±0.00	0.56±0.00	0.55±0.00	0.55±0.00	0.61±0.00	0.61±0.00	0.59±0.00
		test	0.58±0.00	0.57±0.00	0.57±0.00	0.56±0.00	0.60±0.00	0.59±0.00	0.58±0.00
		C1	0.56±0.01	0.56±0.01	0.55±0.00	0.55±0.00	0.60±0.01	0.60±0.01	0.59±0.01
		C2	0.56±0.00	0.55±0.00	0.54±0.00	0.54±0.00	0.59±0.00	0.59±0.00	0.58±0.00
		C3	0.62±0.01	0.61±0.01	0.62±0.00	0.62±0.00	0.61±0.02	0.61±0.02	0.59±0.01
	AP	train	0.46±0.01	0.45±0.01	0.44±0.01	0.44±0.01	0.58±0.01	0.58±0.01	0.56±0.01
		test	0.53±0.01	0.52±0.01	0.51±0.00	0.50±0.00	0.60±0.01	0.60±0.01	0.58±0.01
		C1	0.49±0.01	0.49±0.01	0.47±0.01	0.47±0.01	0.60±0.01	0.60±0.01	0.58±0.01
		C2	0.47±0.01	0.46±0.01	0.44±0.00	0.44±0.00	0.57±0.01	0.56±0.01	0.54±0.01
		C3	0.67±0.02	0.65±0.02	0.67±0.01	0.66±0.01	0.69±0.02	0.68±0.02	0.67±0.02
Para. Filt.	AUC	train	0.62±0.00	0.62±0.00	0.59±0.00	0.58±0.00	0.73±0.01	0.73±0.01	0.72±0.01
		test	0.61±0.00	0.61±0.00	0.56±0.00	0.56±0.00	0.71±0.00	0.71±0.00	0.70±0.00
		C1	0.62±0.01	0.62±0.01	0.59±0.01	0.58±0.01	0.72±0.01	0.72±0.01	0.71±0.01
		C2	0.61±0.00	0.61±0.00	0.56±0.00	0.55±0.00	0.71±0.00	0.70±0.00	0.70±0.00
		C3	0.61±0.01	0.61±0.01	0.56±0.01	0.55±0.01	0.71±0.01	0.70±0.01	0.69±0.01
	pAUC (FPR0.1)	train	0.55±0.00	0.55±0.00	0.53±0.00	0.53±0.00	0.60±0.01	0.60±0.01	0.59±0.00
		test	0.55±0.00	0.55±0.00	0.53±0.00	0.53±0.00	0.59±0.00	0.58±0.00	0.57±0.00
		C1	0.54±0.01	0.55±0.01	0.53±0.00	0.53±0.00	0.59±0.01	0.59±0.01	0.58±0.01
		C2	0.55±0.00	0.55±0.00	0.53±0.00	0.53±0.00	0.58±0.00	0.58±0.00	0.57±0.00
		C3	0.56±0.01	0.56±0.01	0.54±0.00	0.54±0.00	0.59±0.02	0.59±0.01	0.57±0.01
	AP	train	0.41±0.01	0.41±0.01	0.38±0.01	0.38±0.01	0.56±0.01	0.55±0.01	0.53±0.01
		test	0.45±0.01	0.45±0.01	0.41±0.00	0.41±0.00	0.56±0.01	0.56±0.01	0.54±0.01
		C1	0.44±0.01	0.44±0.01	0.41±0.01	0.41±0.01	0.57±0.01	0.57±0.01	0.55±0.01
		C2	0.44±0.00	0.43±0.00	0.39±0.00	0.39±0.00	0.55±0.01	0.54±0.01	0.52±0.01
		C3	0.50±0.03	0.50±0.03	0.45±0.01	0.46±0.01	0.60±0.03	0.59±0.03	0.57±0.03

Performance of functional interaction model when parasites are excluded. The functional interaction model (MLPP) is compared with two other phylogenetic profiling approaches (BPP, NPP). The approaches are compared on three levels, having all species, excluding only parasitic species (“No parasites”) and excluding all clades that contain parasitic species (“No parasitic clades”). The approaches are compared for three metrics – AUC, pROC AUC (pAUC) at FPR of 0.1 and AP, and on five stratifications (see Methods). Values are shown as the mean over cross-validation folds ± standard deviation. MLPP - machine learning phylogenetic profiling (the method presented in this paper), NPP - normalized phylogenetic profiling, BPP - Hamming distance on binarized phylogenetic profiles.

Supplementary Figure 1 - Clademap

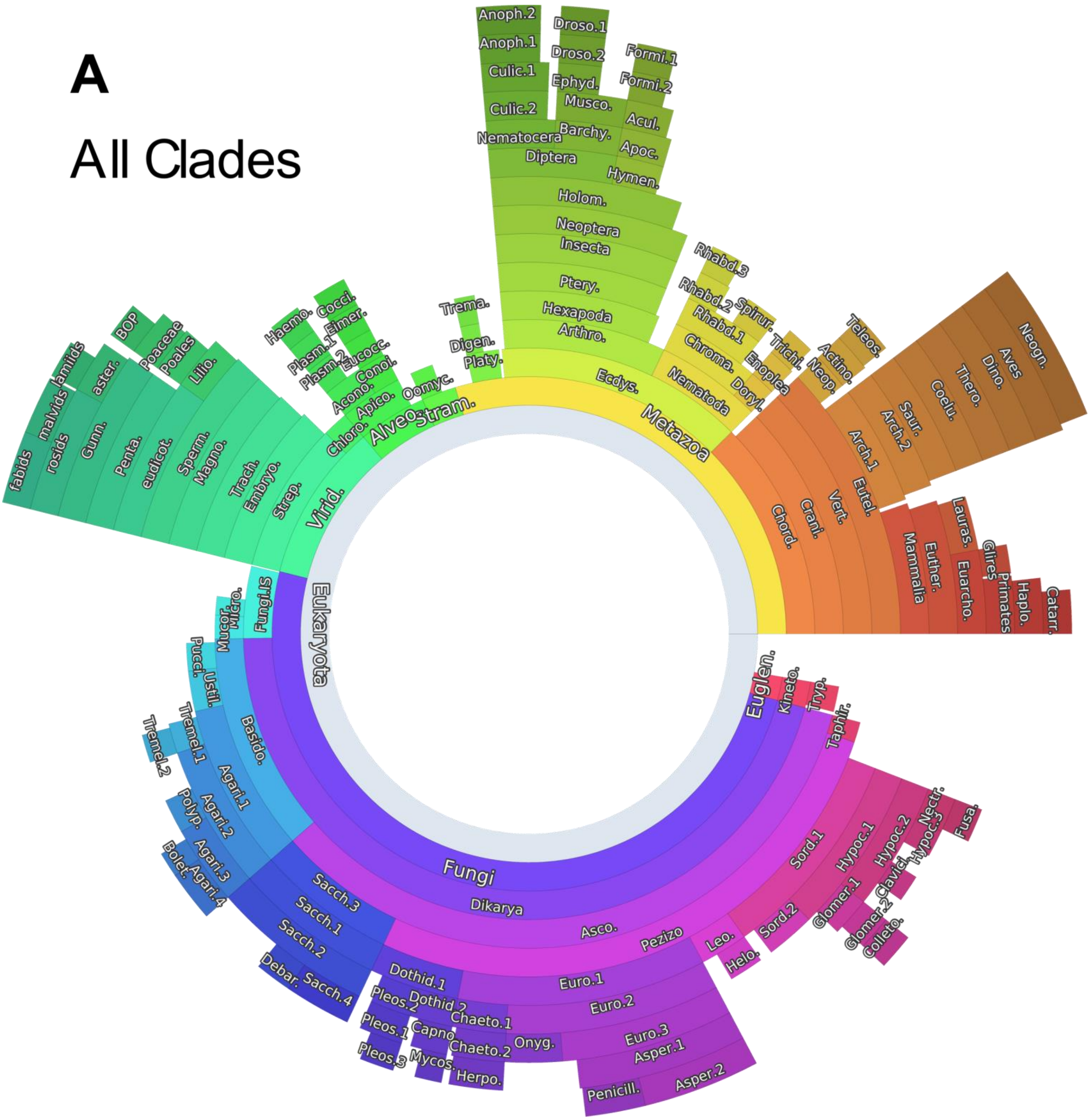

B

Non-Redundant Clades

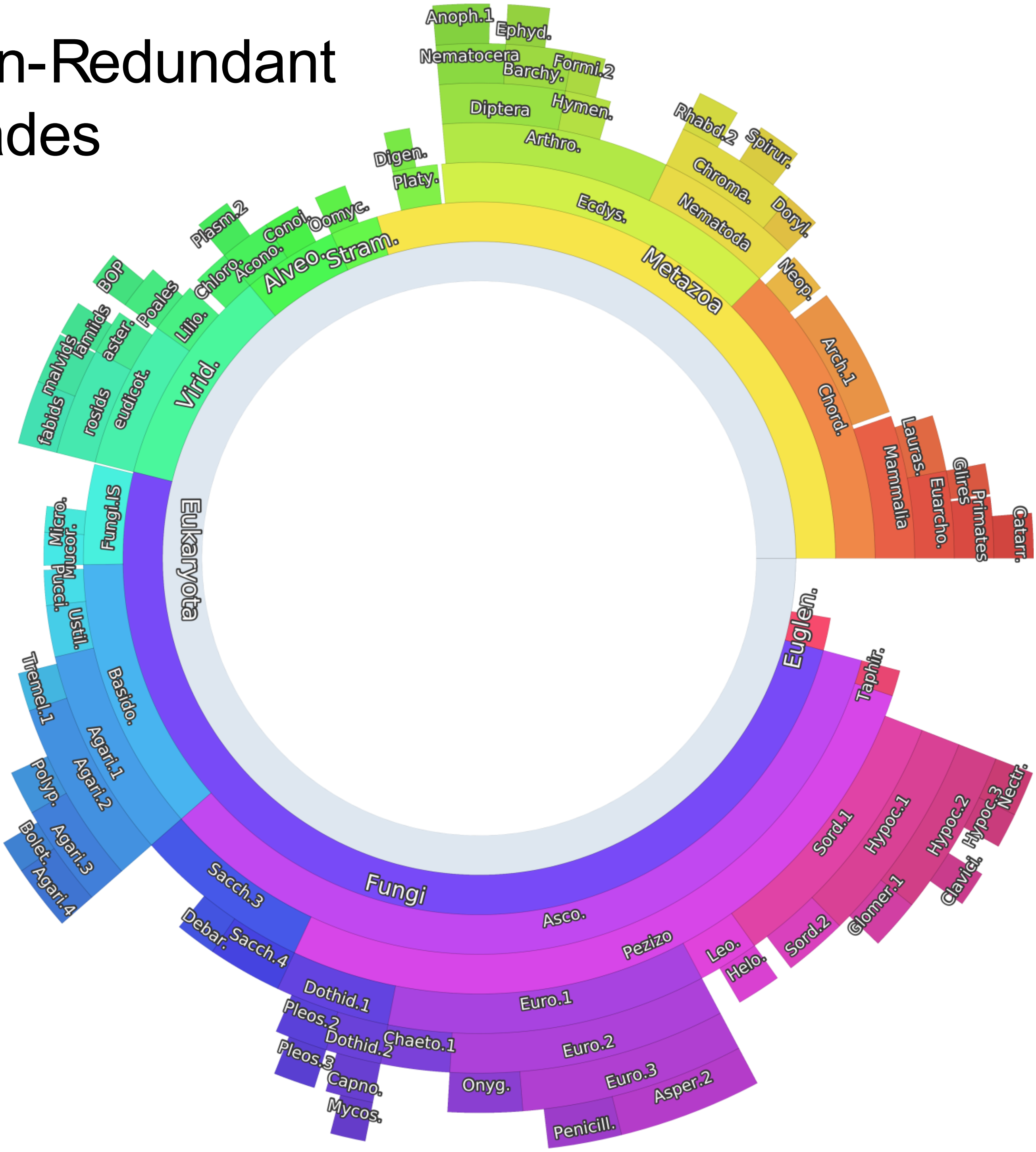

Clademap – Clades are shown hierarchically and sorted by distance from human starting from the 0-degree position (middle right) and colored accordingly by the middle of the clade. Each species can belong to multiple clades, which are sorted by the size of the clade from the inside outward. (A) All clades with more than 10 species. (B) Clades were filtered to remove redundancy.

Supplementary Figure 2 – Positive-Unlabeled Learning

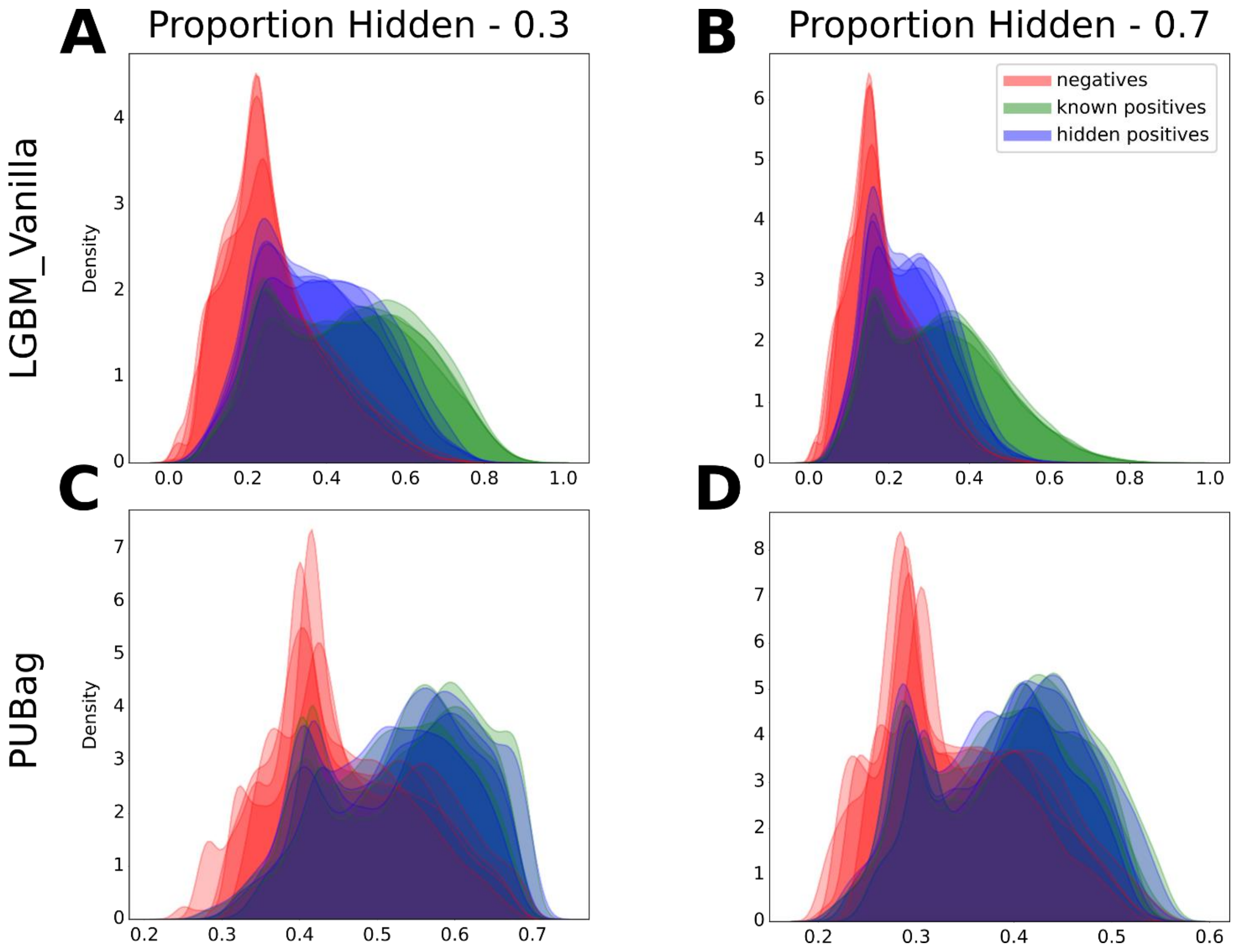

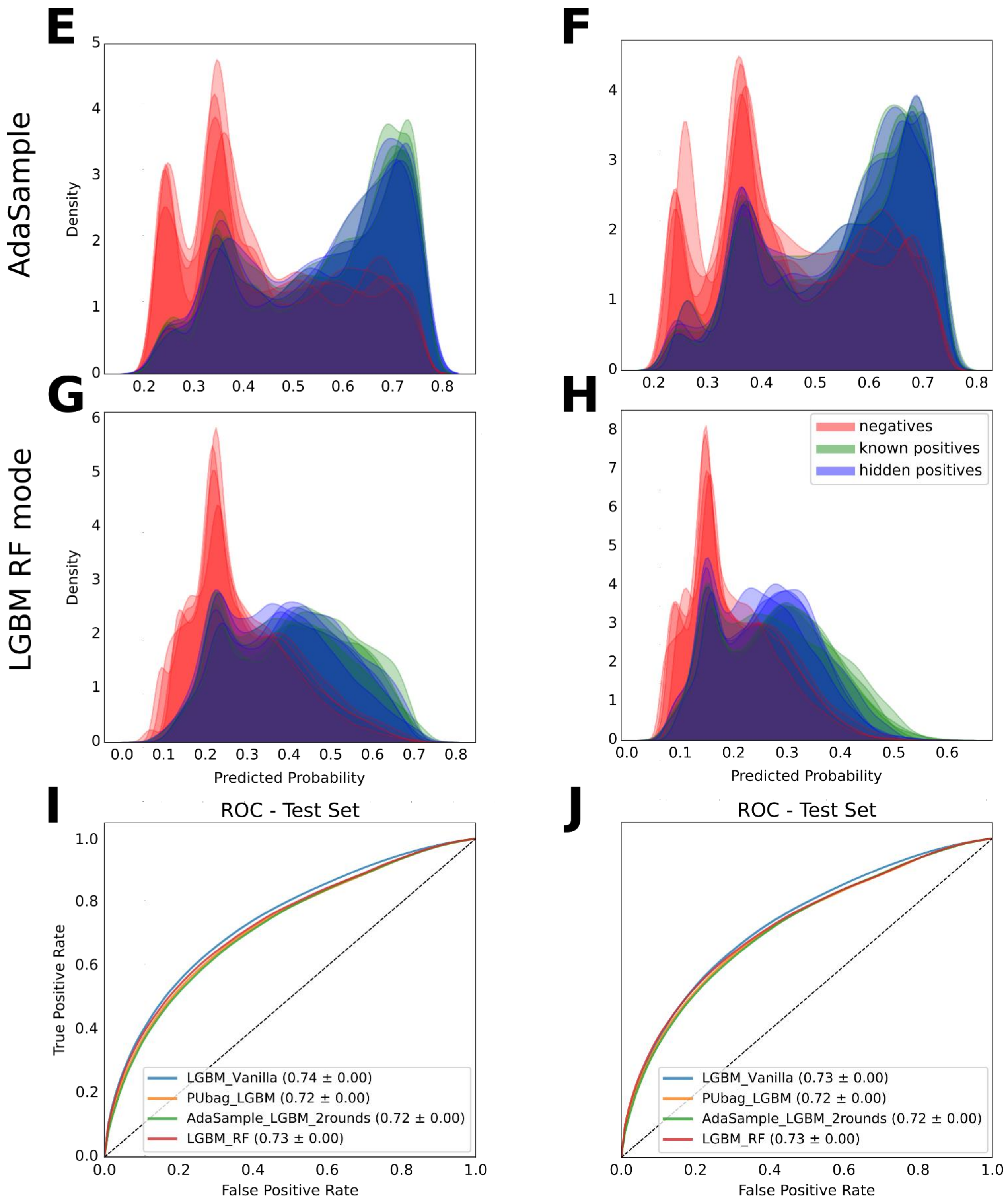

Positive-Unlabeled Learning – (A-H) The probability distributions for gene-pairs belonging to the random negative pairs (red), known positives (green) and hidden positives (blue) are shown for each of the four models tested – LGBM_Vanilla (A,B), PUBag (C,D), AdaSample (E, F) and LGBM RF (G,H) for two probabilities of hiding positives: 0.3 (A,C,E,G) and 0.7 (B,D,F,H). (I-J) ROC curves describing the performance of the different models, trained with 0.3 of the positives hidden (I) or 0.7 (J). Positives are only hidden in the training set (see Supp. Methods). Numbers in brackets are the AUC and the standard deviation of the AUC across cross-validation. LGBM – light gbm, PUBag – positive unlabeled bagging classifier, AdaSample – Adaptive Sampling, RF – random forest, ROC – receiver operator characteristics, AUC – area under the curve. Source data are provided as a Source Data file.

Supplementary Figure 3 - Comparing Machine Learning Algorithms

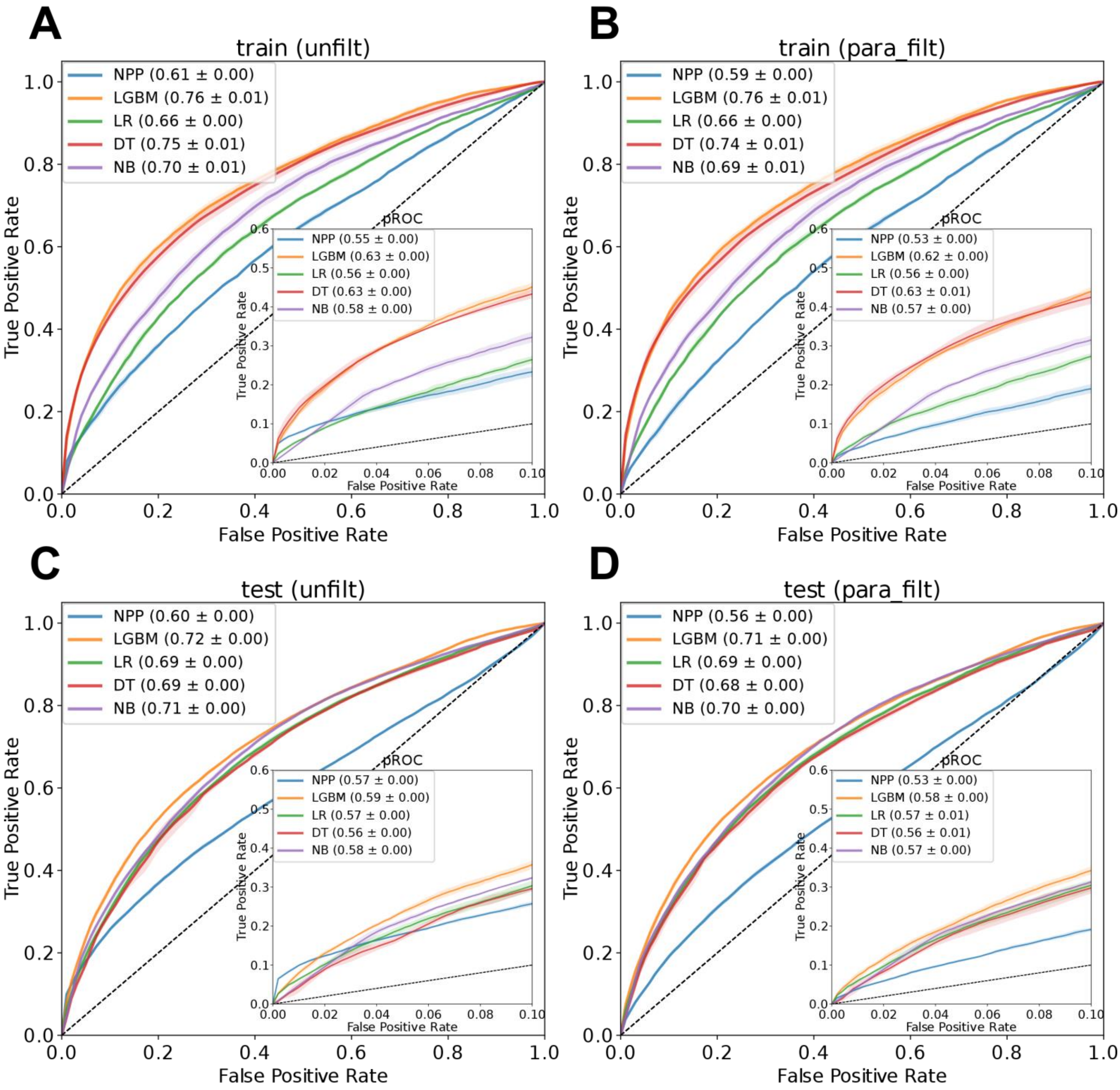

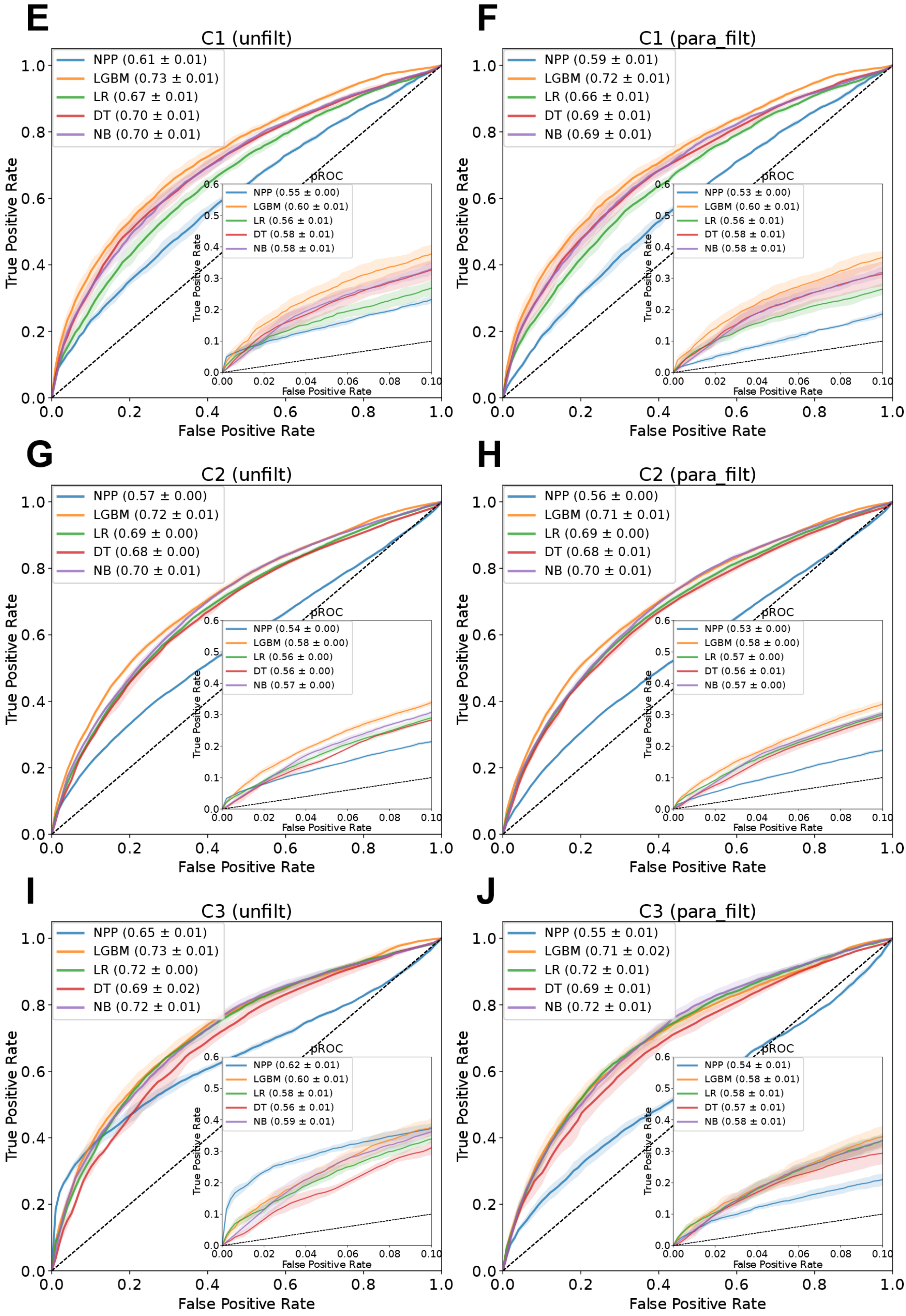
E
C1 (unfilt)
NPP (0.61 ± 0.01)
LGBM (0.73 ± 0.01)
LR (0.67 ± 0.01)
DT (0.70 ± 0.01)
NB (0.70 ± 0.01)
pROC
NPP (0.55 ± 0.00)
LGBM (0.60 ± 0.01)
LR (0.56 ± 0.01)
DT (0.58 ± 0.01)
NB (0.58 ± 0.01)
F
C1 (para_filt)
NPP (0.59 ± 0.01)
LGBM (0.72 ± 0.01)
LR (0.66 ± 0.01)
DT (0.69 ± 0.01)
NB (0.69 ± 0.01)
pROC
NPP (0.53 ± 0.00)
LGBM (0.60 ± 0.01)
LR (0.56 ± 0.01)
DT (0.58 ± 0.01)
NB (0.58 ± 0.01)
G
C2 (unfilt)
NPP (0.57 ± 0.00)
LGBM (0.72 ± 0.01)
LR (0.69 ± 0.00)
DT (0.68 ± 0.00)
NB (0.70 ± 0.01)
pROC
NPP (0.54 ± 0.00)
LGBM (0.58 ± 0.00)
LR (0.56 ± 0.00)
DT (0.56 ± 0.00)
NB (0.57 ± 0.00)
H
C2 (para_filt)
NPP (0.56 ± 0.00)
LGBM (0.71 ± 0.01)
LR (0.69 ± 0.00)
DT (0.68 ± 0.01)
NB (0.70 ± 0.01)
pROC
NPP (0.53 ± 0.00)
LGBM (0.58 ± 0.00)
LR (0.57 ± 0.00)
DT (0.56 ± 0.01)
NB (0.57 ± 0.00)
I
C3 (unfilt)
NPP (0.65 ± 0.01)
LGBM (0.73 ± 0.01)
LR (0.72 ± 0.00)
DT (0.69 ± 0.02)
NB (0.72 ± 0.01)
pROC
NPP (0.62 ± 0.01)
LGBM (0.60 ± 0.01)
LR (0.58 ± 0.01)
DT (0.56 ± 0.01)
NB (0.59 ± 0.01)
J
C3 (para_filt)
NPP (0.55 ± 0.01)
LGBM (0.71 ± 0.02)
LR (0.72 ± 0.01)
DT (0.69 ± 0.01)
NB (0.72 ± 0.01)
pROC
NPP (0.54 ± 0.01)
LGBM (0.58 ± 0.01)
LR (0.58 ± 0.01)
DT (0.57 ± 0.01)
NB (0.58 ± 0.01)
True Positive Rate
False Positive Rate

Model Performance (Functional Interaction Model) – Comparing Machine Learning Algorithms. (A-J) ML algorithms were compared on the basis of predicting gene pair functional interaction (co-occurrence in Reactome pathway). Performance was measured using a ROC curve and the AUC. In the inset of each panel is a pROC curve for the FPR range (0-0.1). LGBM was chosen based on superior performance. C1 (E-F), C2 (G-H), and C3 (I-J) are stratifications for pairwise prediction for pairs of genes appearing both in the training set, one appearing in the training set, and not appearing in the training set, respectively (see Methods, based on [1]). Model evaluation was conducted with pairs of paralogs (A, C, E, G, I) or without (B, D, F, H, J). Numbers in brackets are the AUC and the standard deviation of the AUC in cross-validations. NPP - normalized phylogenetic profiling, LGBM - light gradient boosting machine (lightGBM) in random forest mode, LR - logistic regression, DT - decision tree, NB - naive bayes, ROC - receiver operator characteristics, AUC - area under the curve, pROC - partial ROC curve, FPR - false positive rate. Source data are provided as a Source Data file.

Supplementary Figure 4 – “Young” Genes

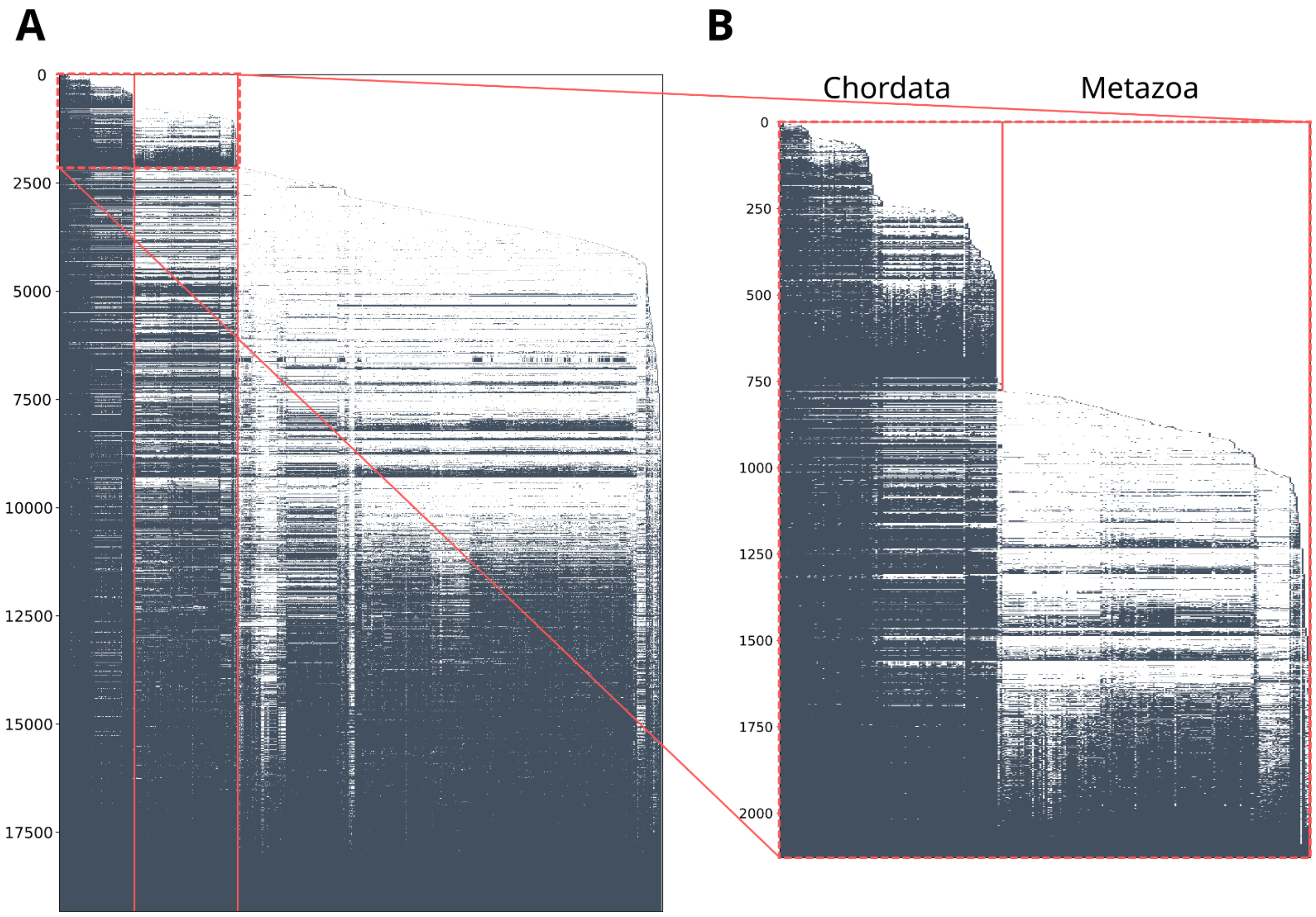

C

		ROC AUC			pROC AUC			AP		
		All genes – 1.7e5 pairs	Metazoa – 2.4e3 pairs	Chordata – 4.5e2 pairs	All genes – 1.7e5 pairs	Metazoa – 2.4e3 pairs	Chordata – 4.5e2 pairs	All genes – 1.7e5 pairs	Metazoa – 2.4e3 pairs	Chordata – 4.5e2 pairs
Unfilt	MLPP	0.73 ±0.00	0.69 ±0.02	0.70 ±0.04	0.60 ±0.00	0.58 ±0.01	0.54 ±0.02	0.76 ±0.00	0.76 ±0.03	0.88 ±0.03
	NPP	0.60 ±0.00	0.75 ±0.00	0.77 ±0.03	0.56 ±0.00	0.67 ±0.01	0.65 ±0.04	0.66 ±0.00	0.83 ±0.00	0.92 ±0.02
	SVD-Phy	0.59 ±0.00	0.73 ±0.01	0.76 ±0.02	0.55 ±0.00	0.59 ±0.01	0.64 ±0.02	0.65 ±0.00	0.78 ±0.02	0.92 ±0.01
	PPP	0.63 ±0.00	0.79 ±0.01	0.81 ±0.03	0.57 ±0.00	0.71 ±0.01	0.67 ±0.04	0.69 ±0.00	0.86 ±0.00	0.94 ±0.02
	Hamming	0.64 ±0.00	0.79 ±0.01	0.82 ±0.02	0.58 ±0.00	0.70 ±0.01	0.65 ±0.03	0.69 ±0.00	0.86 ±0.01	0.93 ±0.01

D

		ROC AUC			pROC AUC			AP		
		All genes – 1.6e5 pairs	Metazoa – 1.9e3 pairs	Chordata – 3.7e2 pairs	All genes – 1.6e5 pairs	Metazoa – 1.9e3 pairs	Chordata – 3.7e2 pairs	All genes – 1.6e5 pairs	Metazoa – 1.9e3 pairs	Chordata – 3.7e2 pairs
Para. filt.	MLPP	0.72 ±0.00	0.65 ±0.02	0.69 ±0.04	0.59 ±0.00	0.54 ±0.01	0.55 ±0.02	0.72 ±0.00	0.64 ±0.03	0.85 ±0.03
	NPP	0.56 ±0.00	0.67 ±0.01	0.72 ±0.04	0.53 ±0.00	0.58 ±0.01	0.59 ±0.04	0.58 ±0.00	0.69 ±0.01	0.87 ±0.03
	SVD-Phy	0.55 ±0.00	0.64 ±0.01	0.70 ±0.02	0.53 ±0.00	0.52 ±0.01	0.57 ±0.02	0.57 ±0.00	0.60 ±0.02	0.85 ±0.02
	PPP	0.59 ±0.00	0.70 ±0.01	0.77 ±0.04	0.53 ±0.00	0.61 ±0.01	0.60 ±0.04	0.60 ±0.00	0.73 ±0.01	0.89 ±0.03
	Hamming	0.61 ±0.00	0.71 ±0.01	0.78 ±0.02	0.55 ±0.00	0.60 ±0.01	0.59 ±0.02	0.62 ±0.00	0.72 ±0.01	0.89 ±0.02

Young Genes – (A) Phylogenetic profiles as binary presence or absence of genes. The genes are stratified for genes found exclusively in Chordata or Metazoa (inclusive). (B) Zoom-in on the inset in (A). In both A-B, rows are genes and columns are organisms ordered as in Supp. Figure 1. Blue represents presence. Genes are ordered by the farthest shared ancestor with human from the closest (e.g. mammalian specific genes) to farthest (e.g. genes found in all eukaryotes) and from sparsely present to highly present.

Supplementary Figure 5 - Model Performance - Comparing Phylogenetic Profiling Approaches

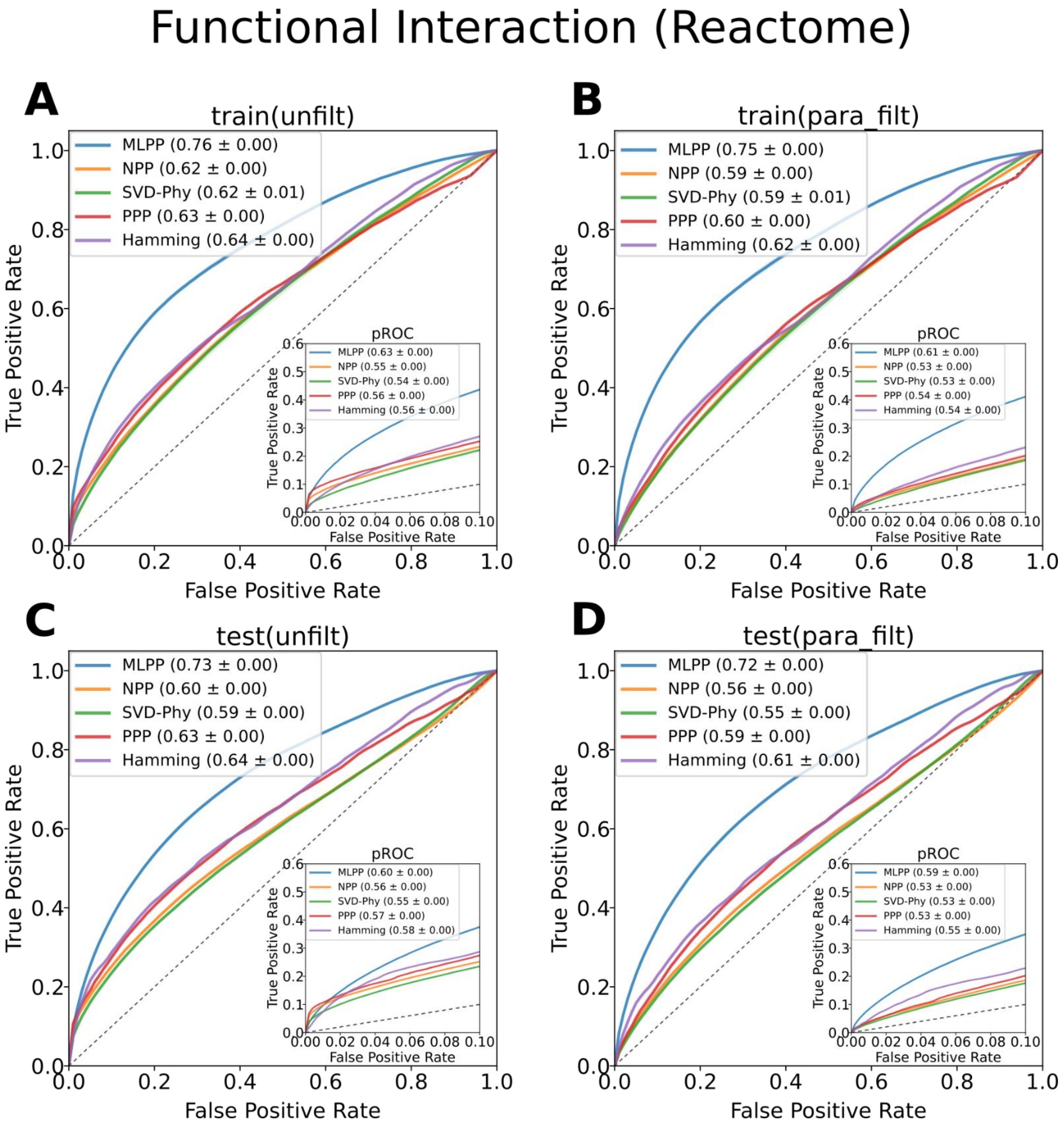

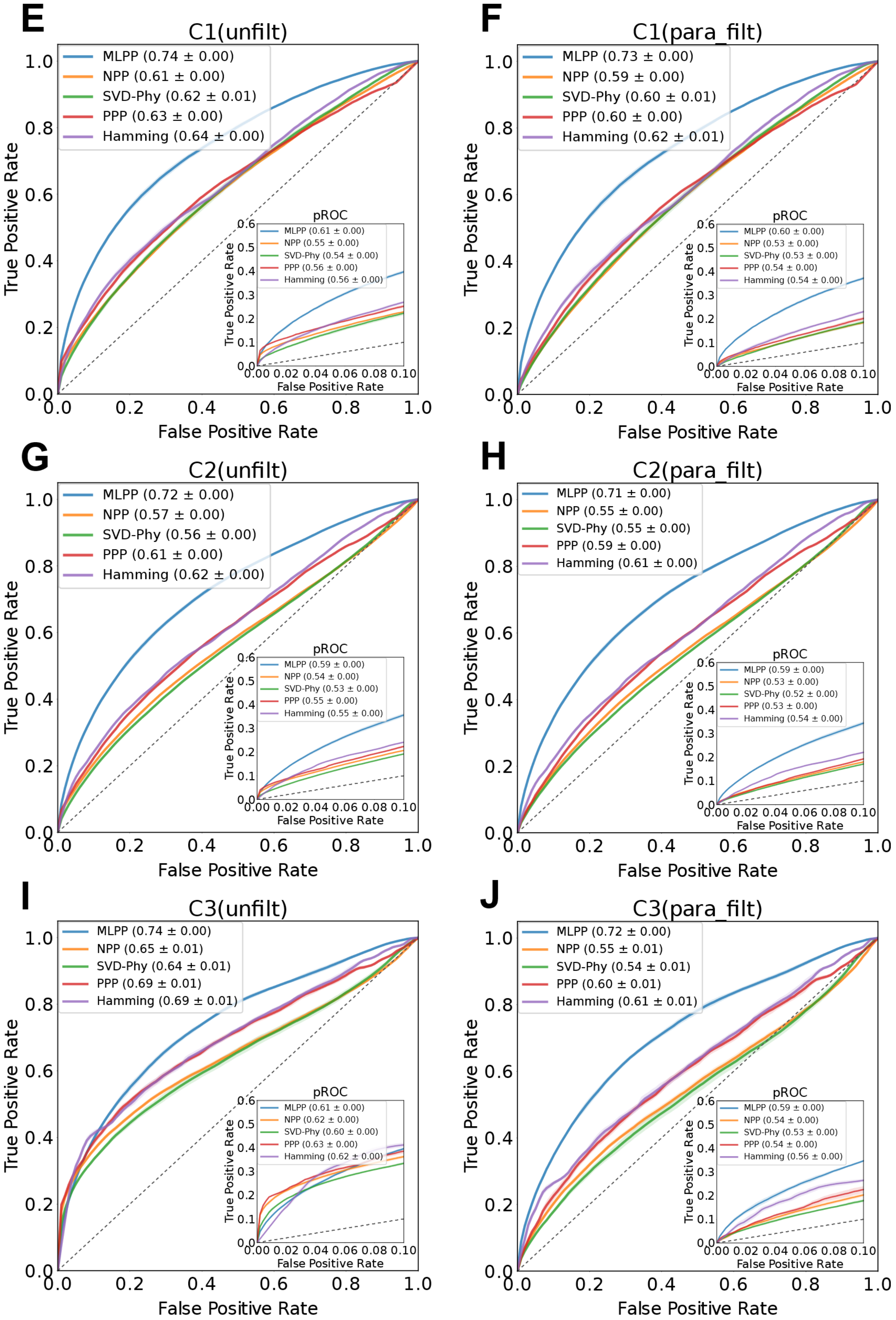
E
C1(unfilt)
MLPP (0.74 ± 0.00)
NPP (0.61 ± 0.00)
SVD-Phy (0.62 ± 0.01)
PPP (0.63 ± 0.00)
Hamming (0.64 ± 0.00)
pROC
MLPP (0.61 ± 0.00)
NPP (0.55 ± 0.00)
SVD-Phy (0.54 ± 0.00)
PPP (0.56 ± 0.00)
Hamming (0.56 ± 0.00)
F
C1(para_filt)
MLPP (0.73 ± 0.00)
NPP (0.59 ± 0.00)
SVD-Phy (0.60 ± 0.01)
PPP (0.60 ± 0.00)
Hamming (0.62 ± 0.01)
pROC
MLPP (0.60 ± 0.00)
NPP (0.53 ± 0.00)
SVD-Phy (0.53 ± 0.00)
PPP (0.54 ± 0.00)
Hamming (0.54 ± 0.00)
G
C2(unfilt)
MLPP (0.72 ± 0.00)
NPP (0.57 ± 0.00)
SVD-Phy (0.56 ± 0.00)
PPP (0.61 ± 0.00)
Hamming (0.62 ± 0.00)
pROC
MLPP (0.59 ± 0.00)
NPP (0.54 ± 0.00)
SVD-Phy (0.53 ± 0.00)
PPP (0.55 ± 0.00)
Hamming (0.55 ± 0.00)
H
C2(para_filt)
MLPP (0.71 ± 0.00)
NPP (0.55 ± 0.00)
SVD-Phy (0.55 ± 0.00)
PPP (0.59 ± 0.00)
Hamming (0.61 ± 0.00)
pROC
MLPP (0.59 ± 0.00)
NPP (0.53 ± 0.00)
SVD-Phy (0.52 ± 0.00)
PPP (0.53 ± 0.00)
Hamming (0.54 ± 0.00)
I
C3(unfilt)
MLPP (0.74 ± 0.00)
NPP (0.65 ± 0.01)
SVD-Phy (0.64 ± 0.01)
PPP (0.69 ± 0.01)
Hamming (0.69 ± 0.01)
pROC
MLPP (0.61 ± 0.00)
NPP (0.62 ± 0.00)
SVD-Phy (0.60 ± 0.00)
PPP (0.63 ± 0.00)
Hamming (0.62 ± 0.00)
J
C3(para_filt)
MLPP (0.72 ± 0.00)
NPP (0.55 ± 0.01)
SVD-Phy (0.54 ± 0.01)
PPP (0.60 ± 0.01)
Hamming (0.61 ± 0.01)
pROC
MLPP (0.59 ± 0.00)
NPP (0.54 ± 0.00)
SVD-Phy (0.53 ± 0.00)
PPP (0.54 ± 0.00)
Hamming (0.56 ± 0.00)
True Positive Rate
False Positive Rate

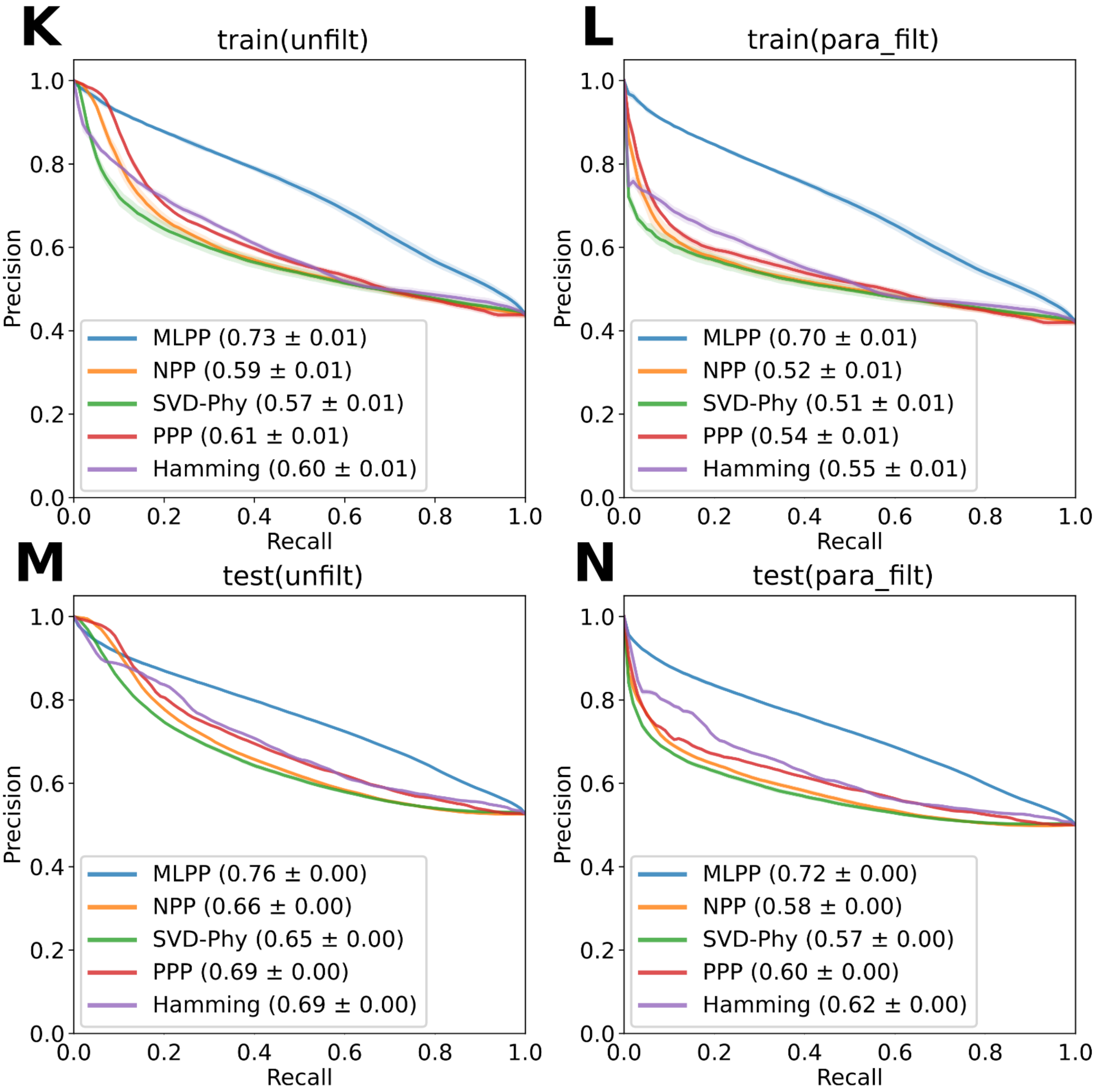
K
train(unfilt)
Precision
Recall
MLPP (0.73 ± 0.01)
NPP (0.59 ± 0.01)
SVD-Phy (0.57 ± 0.01)
PPP (0.61 ± 0.01)
Hamming (0.60 ± 0.01)
L
train(para_filt)
MLPP (0.70 ± 0.01)
NPP (0.52 ± 0.01)
SVD-Phy (0.51 ± 0.01)
PPP (0.54 ± 0.01)
Hamming (0.55 ± 0.01)
M
test(unfilt)
MLPP (0.76 ± 0.00)
NPP (0.66 ± 0.00)
SVD-Phy (0.65 ± 0.00)
PPP (0.69 ± 0.00)
Hamming (0.69 ± 0.00)
N
test(para_filt)
MLPP (0.72 ± 0.00)
NPP (0.58 ± 0.00)
SVD-Phy (0.57 ± 0.00)
PPP (0.60 ± 0.00)
Hamming (0.62 ± 0.00)

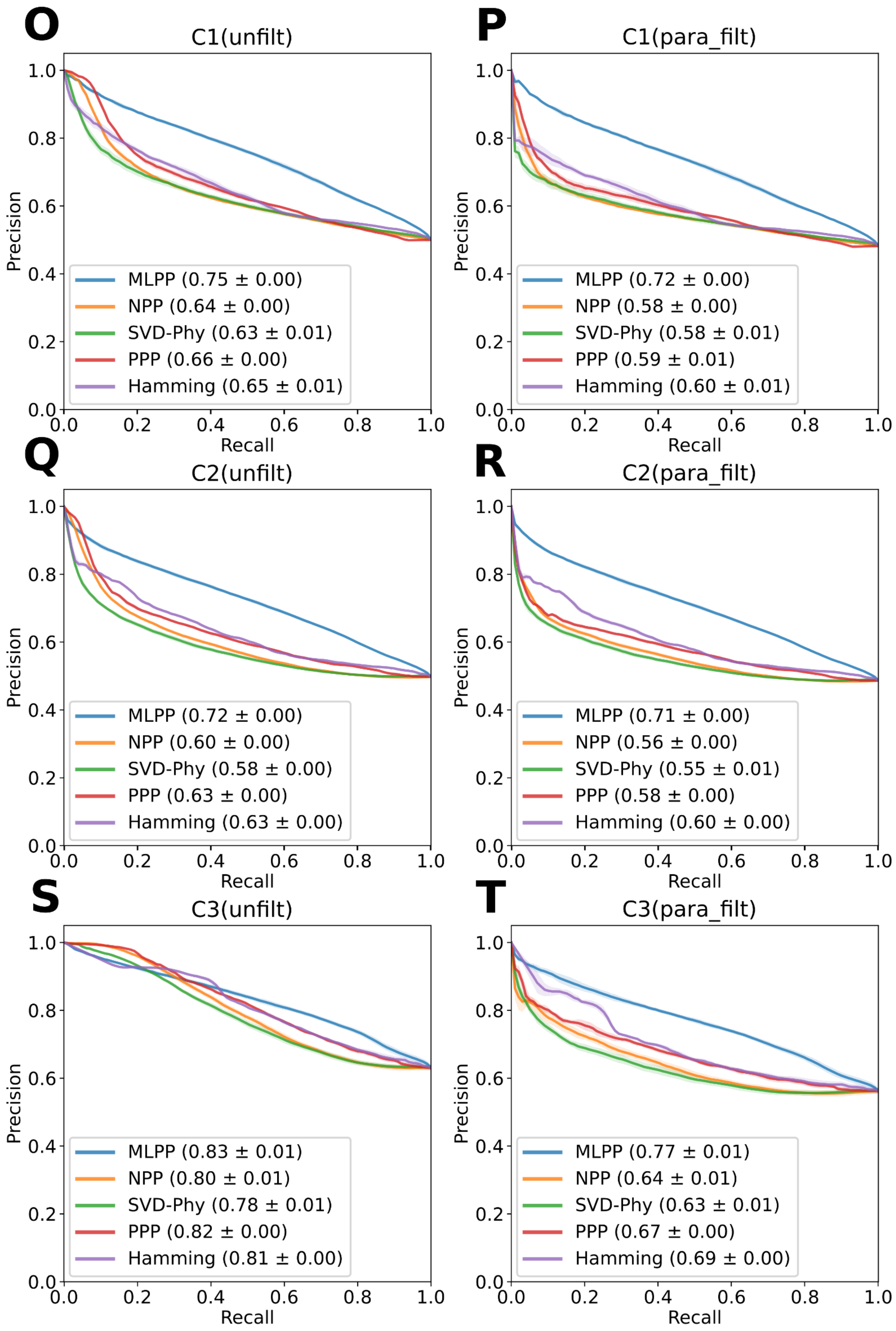
O
C1(unfilt)
MLPP (0.75 ± 0.00)
NPP (0.64 ± 0.00)
SVD-Phy (0.63 ± 0.01)
PPP (0.66 ± 0.00)
Hamming (0.65 ± 0.01)
P
C1(para_filt)
MLPP (0.72 ± 0.00)
NPP (0.58 ± 0.00)
SVD-Phy (0.58 ± 0.01)
PPP (0.59 ± 0.01)
Hamming (0.60 ± 0.01)
Q
C2(unfilt)
MLPP (0.72 ± 0.00)
NPP (0.60 ± 0.00)
SVD-Phy (0.58 ± 0.00)
PPP (0.63 ± 0.00)
Hamming (0.63 ± 0.00)
R
C2(para_filt)
MLPP (0.71 ± 0.00)
NPP (0.56 ± 0.00)
SVD-Phy (0.55 ± 0.01)
PPP (0.58 ± 0.00)
Hamming (0.60 ± 0.00)
S
C3(unfilt)
MLPP (0.83 ± 0.01)
NPP (0.80 ± 0.01)
SVD-Phy (0.78 ± 0.01)
PPP (0.82 ± 0.00)
Hamming (0.81 ± 0.00)
T
C3(para_filt)
MLPP (0.77 ± 0.01)
NPP (0.64 ± 0.01)
SVD-Phy (0.63 ± 0.01)
PPP (0.67 ± 0.00)
Hamming (0.69 ± 0.00)
Precision
Recall
0.0
0.2
0.4
0.6
0.8
1.0

Model Performance (Functional Interaction Model) - Comparing Phylogenetic Profiling Approaches. (A-J) PP algorithms were compared on the basis of predicting gene pair functional interaction (co-occurrence in Reactome pathway). Performance was measured using a ROC curve and the AUC (A-J) as well as the PR curve and its AP (K-T). In the inset of each panel is a pROC curve for the FPR range (0-0.1). C1 (E-F, O-P), C2 (G-H, Q-R), and C3 (I-J, S-T) are stratifications for pairwise prediction where both, one, or none of a pair of genes appears in the training set, respectively (see Methods, based on [1]). Model evaluation was conducted with (A, C, E, G, I, K, M, O, Q, S) or without (B, D, F, H, J, L, N, P, R, T) paralogous pairs. Numbers in brackets are the AUC/AP and the standard deviation of the AUC/AP in cross-validations for ROC/PR curves, respectively. Numbers in brackets for the insets are the pAUC and standard deviation. MLPP - machine learning phylogenetic profiling (the method presented in this paper), NPP - normalized phylogenetic profiling, PPP - PrePhyloPro, Hamming - Hamming distance on binarized phylogenetic profiles, ROC - receiver operator characteristics, AUC - area under the curve, pROC - partial ROC curve, FPR - false positive rate, PR – precision recall, A_ - average precision. Source data are provided as a Source Data file.

Supplementary Figure 6 – Reactome Temporal Splits

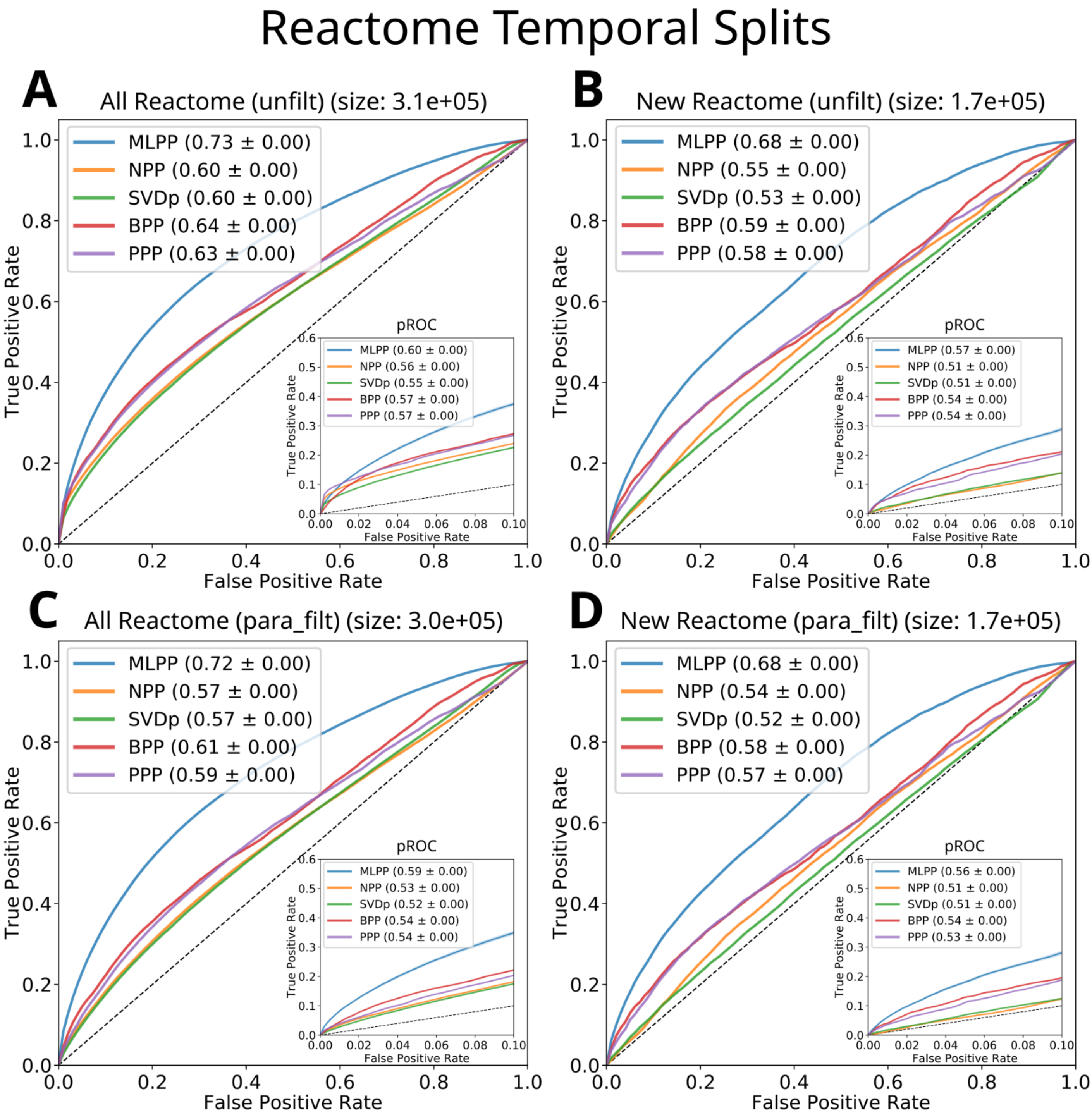

Reactome Temporal Splits- (A-H) Model performance in functional interactions from Reactome (01.2021) using a model trained on an earlier snapshot (02.2019). Performance was measured by a ROC curve and the AUC. In the inset of each panel is a pROC curve for the FPR range (0-0.1). Model evaluation was conducted with (A, B, E, F - “unfilt”) or without (C, D, G, H - “para_filt”) paralogous pairs, and with (A, C, E, G) or without (B, D, F, H) pairs that appear in the older Reactome snapshot used for training. Numbers in brackets are the AUC and the standard deviation of the AUC in cross-validations. ROC - receiver operator characteristics, AUC - area under the curve, pROC - partial ROC curve, FPR - false positive rate. Source data are provided as a Source Data file.

Supplementary Figure 7 - External Validation – PPI

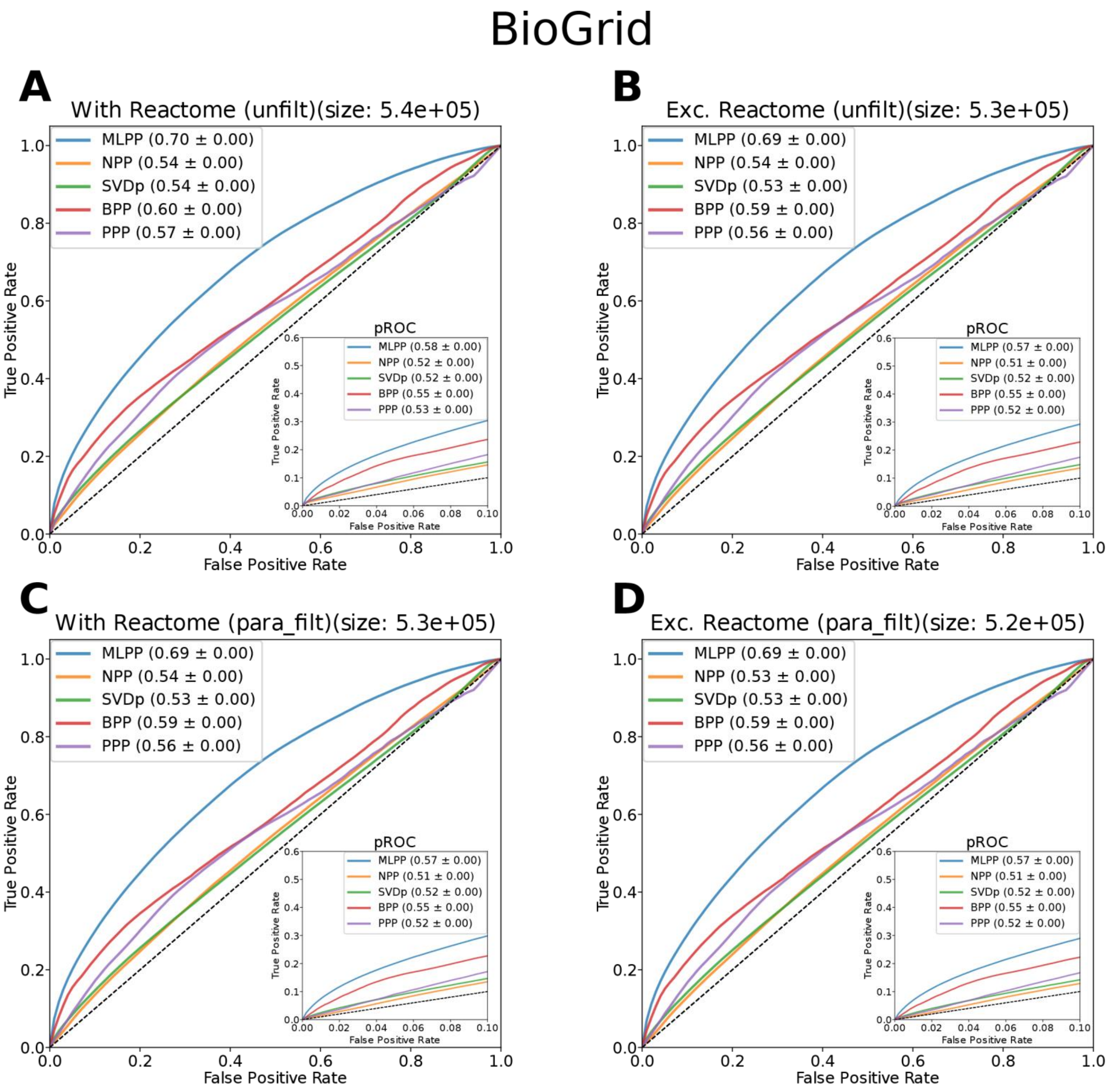

IntAct

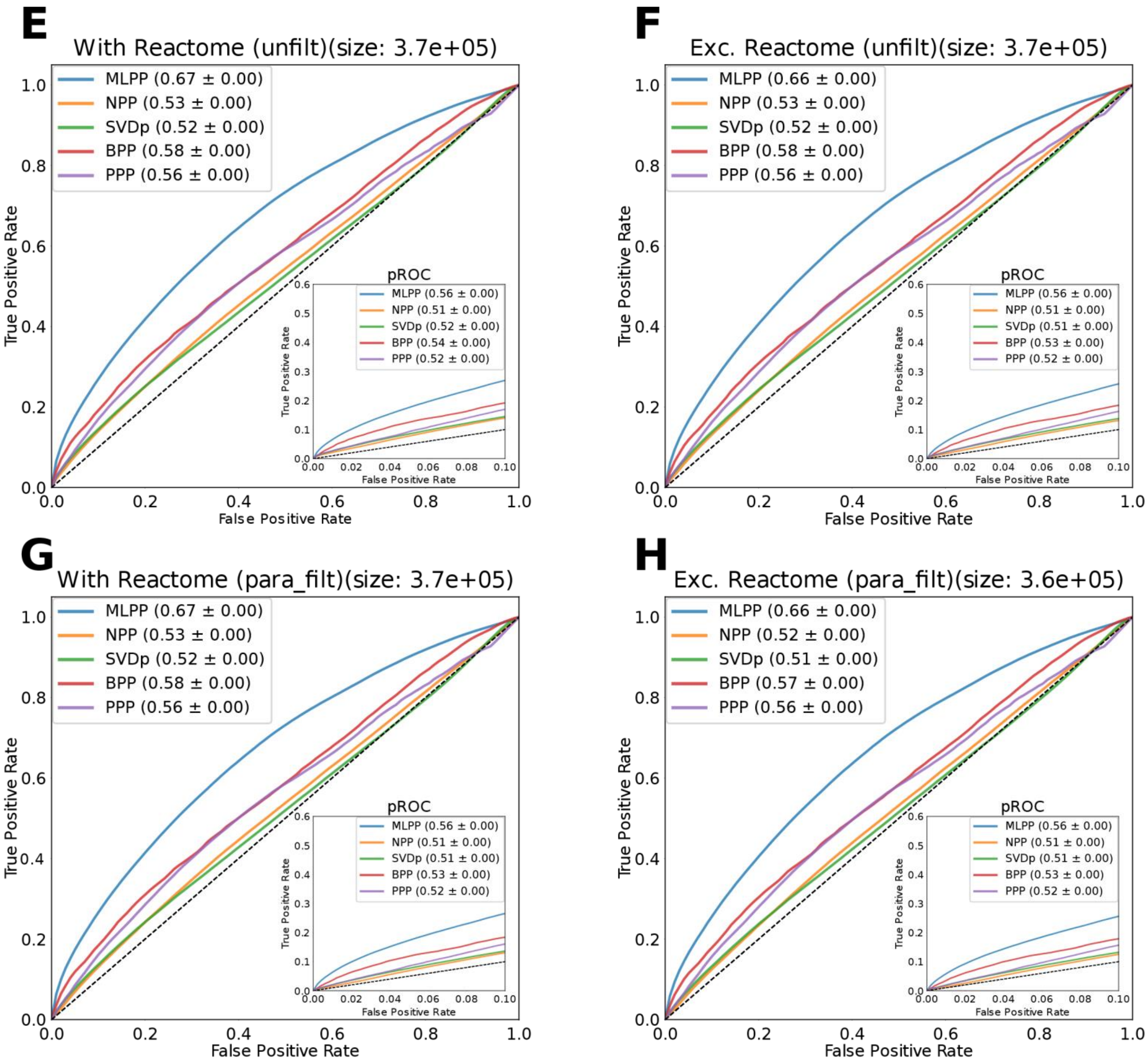

External Validation – PPI - (A-H) Model performance in predicting protein-protein interactions (PPI) in BioGrid (A-D) or the IntAct (E-H) databases. Performance was measured by a ROC curve and the AUC. In the inset of each panel is a pROC curve for the FPR range (0-0.1). Model evaluation was conducted with pairs of paralogs (A, B, E, F - “unfilt”) or without (C, D, G, H - “para_filt”) and with pairs that appear in Reactome, the database used for training (A, C, E, G) or without (B, D, F, H). Numbers in brackets are the AUC and the standard deviation of the AUC in cross-validations. ROC - receiver operator characteristics, AUC - area under the curve, pROC - partial ROC curve, FPR - false positive rate.

Supplementary Figure 8 - External Validation – KEGG

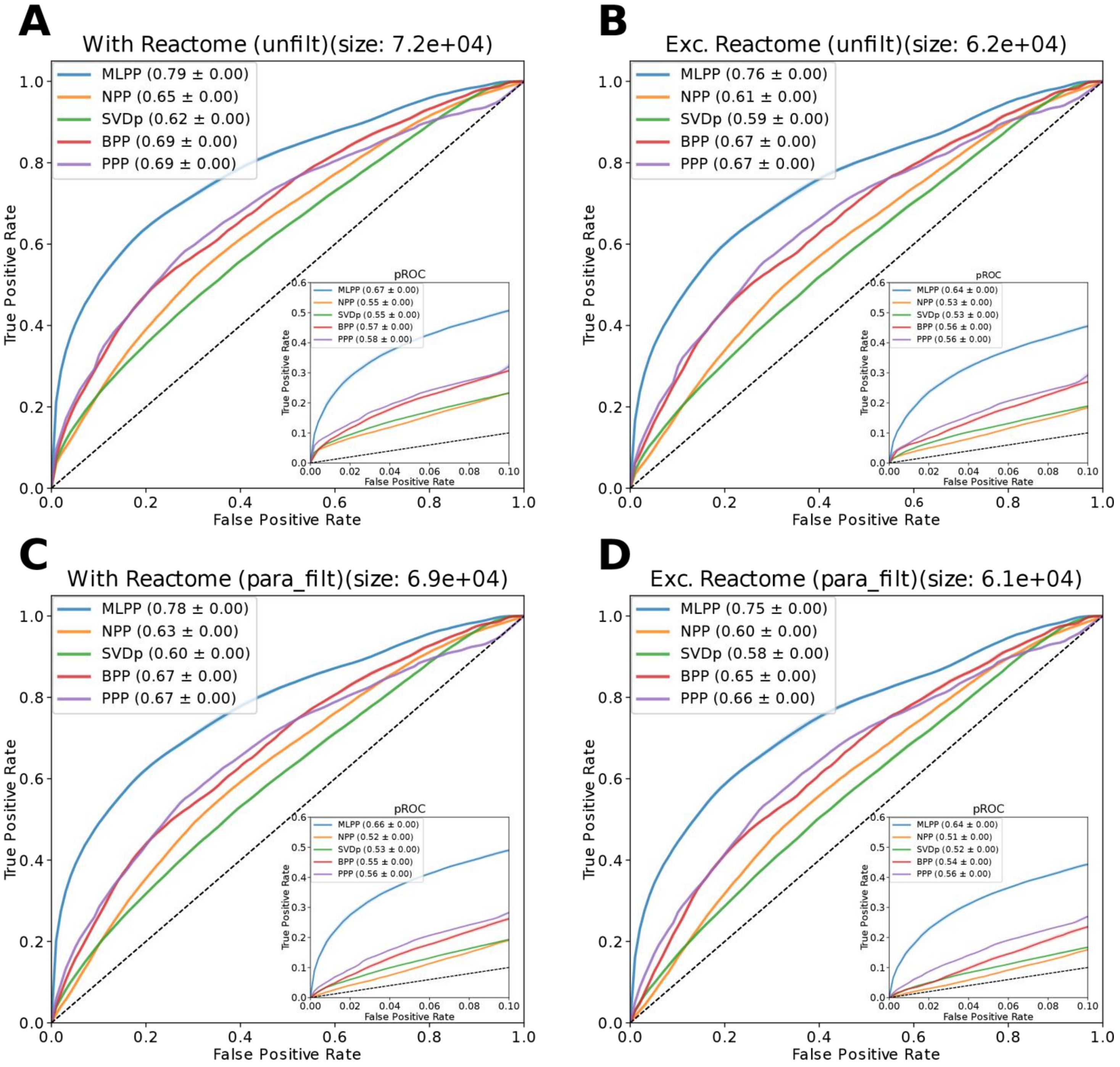

External Validation – KEGG - (A-D) Model performance in predicting gene pair co-occurrence in KEGG pathways as measured by a ROC curve and the AUC. In the inset of each panel is a pROC curve for the FPR range (0-0.1). Model evaluation was conducted with pairs of paralogs (A, B - "unfilt") or without (C, D - "para_filt") and with pairs that appear in Reactome, the database used for training (A,C) or without (B,D). Numbers in brackets are the AUC and the standard deviation of the AUC in cross-validations. ROC - receiver operator characteristics, AUC - area under the curve, pROC - partial ROC curve, FPR - false positive rate.

Supplementary Figure 9 - External Validation – Complexes (Functional Interaction Model)

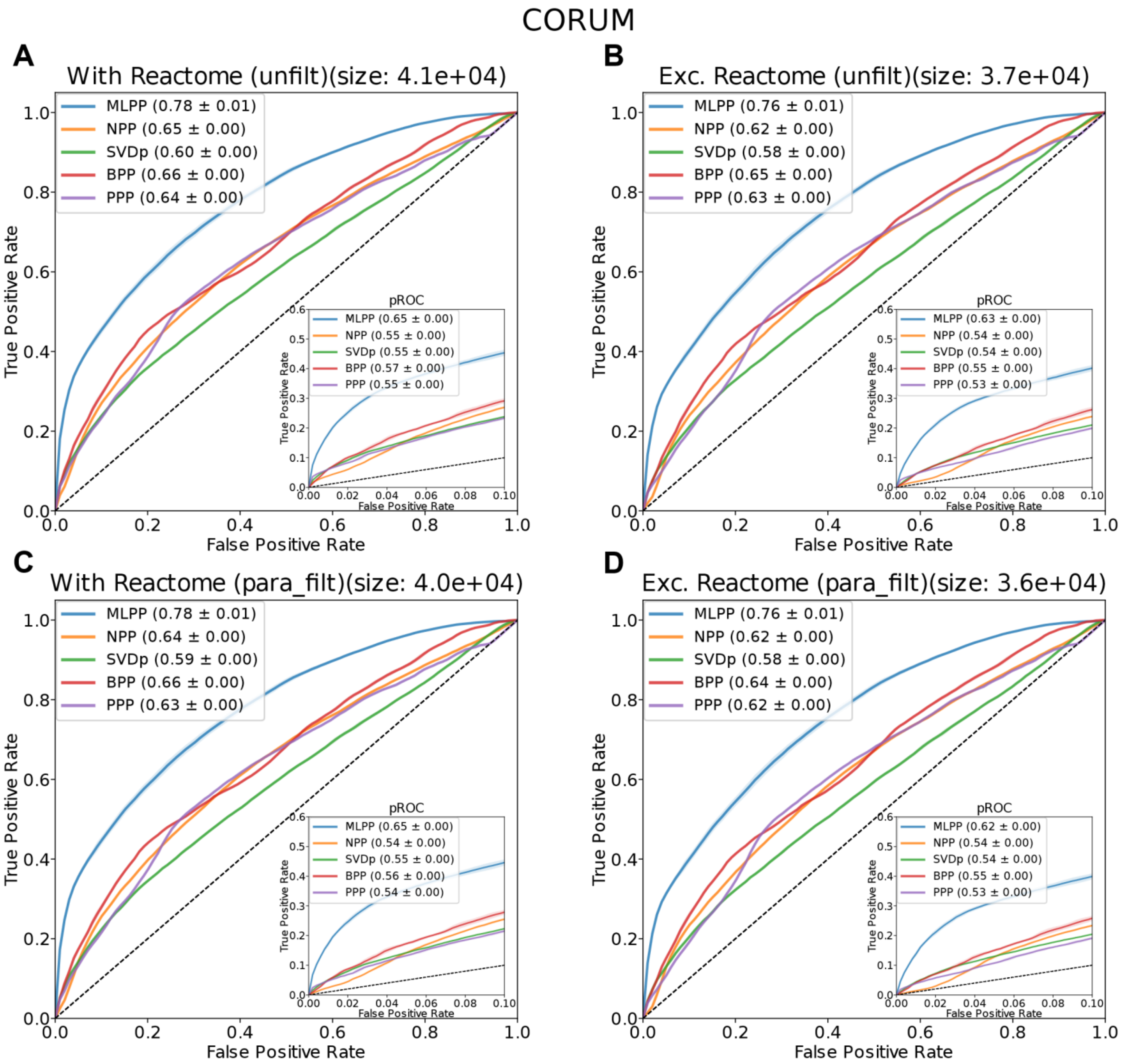

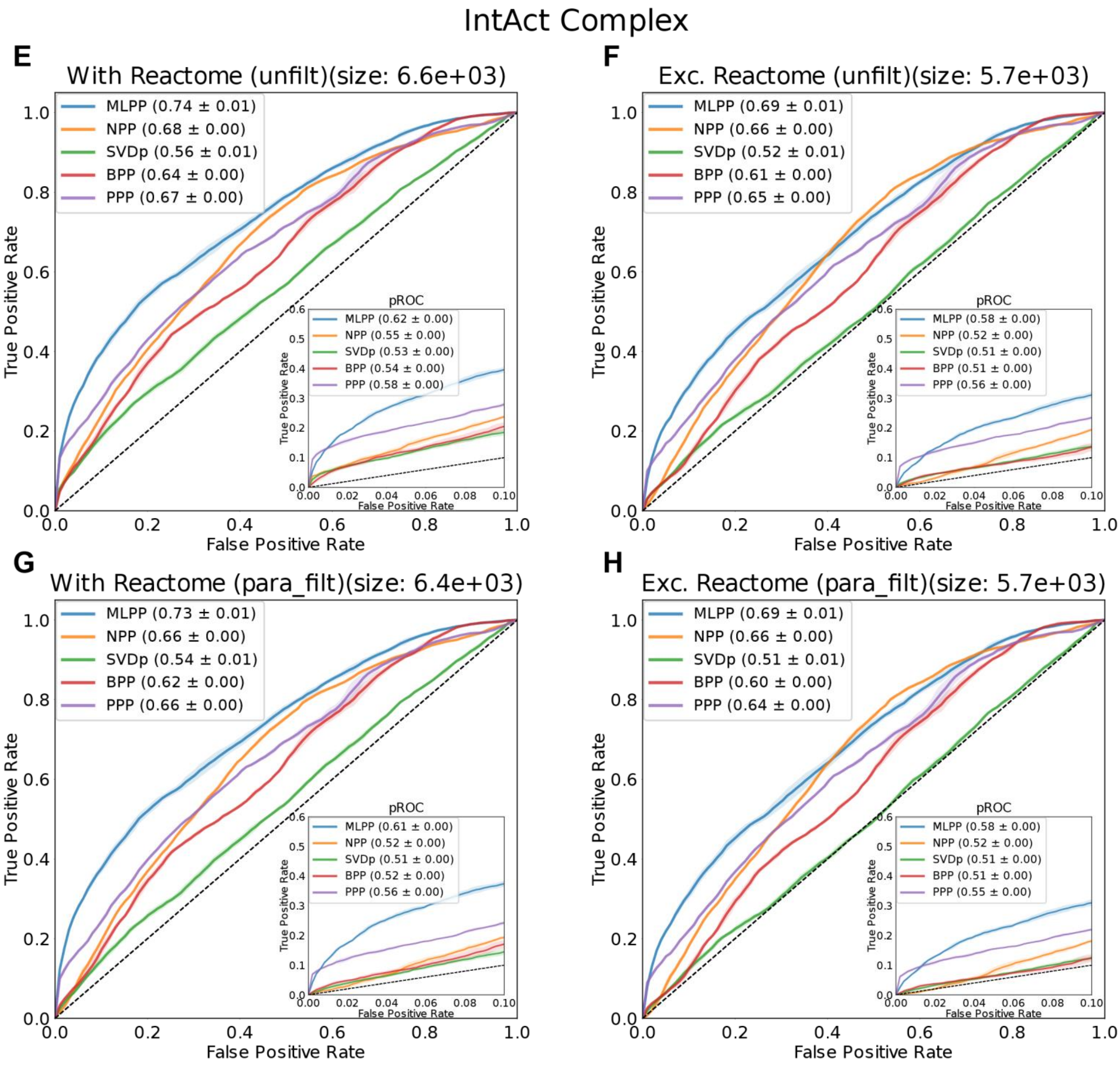

External Validation – Complexes (Functional Interaction Model) - (A-H) Model performance in predicting complex co-occurrence in CORUM (A-D) or the IntAct Complex (E-H) databases. Performance was measured by a ROC curve and the AUC and using the Functional Interaction model. In the inset of each panel is a pROC curve for the FPR range (0-0.1). Model evaluation was conducted with pairs of paralogs (A, B, E, F - "unfilt") or without (C, D, G, H - "para_filt") and with pairs that appear in Reactome, the database used for training (A, C, E, G) or without (B, D, F, H). Numbers in brackets are the AUC and the standard deviation of the AUC in cross-validations. ROC - receiver operator characteristics, AUC - area under the curve, pROC - partial ROC curve, FPR - false positive rate.

Supplementary Figure 10 - External Validation – Complexes (In Complex Model)

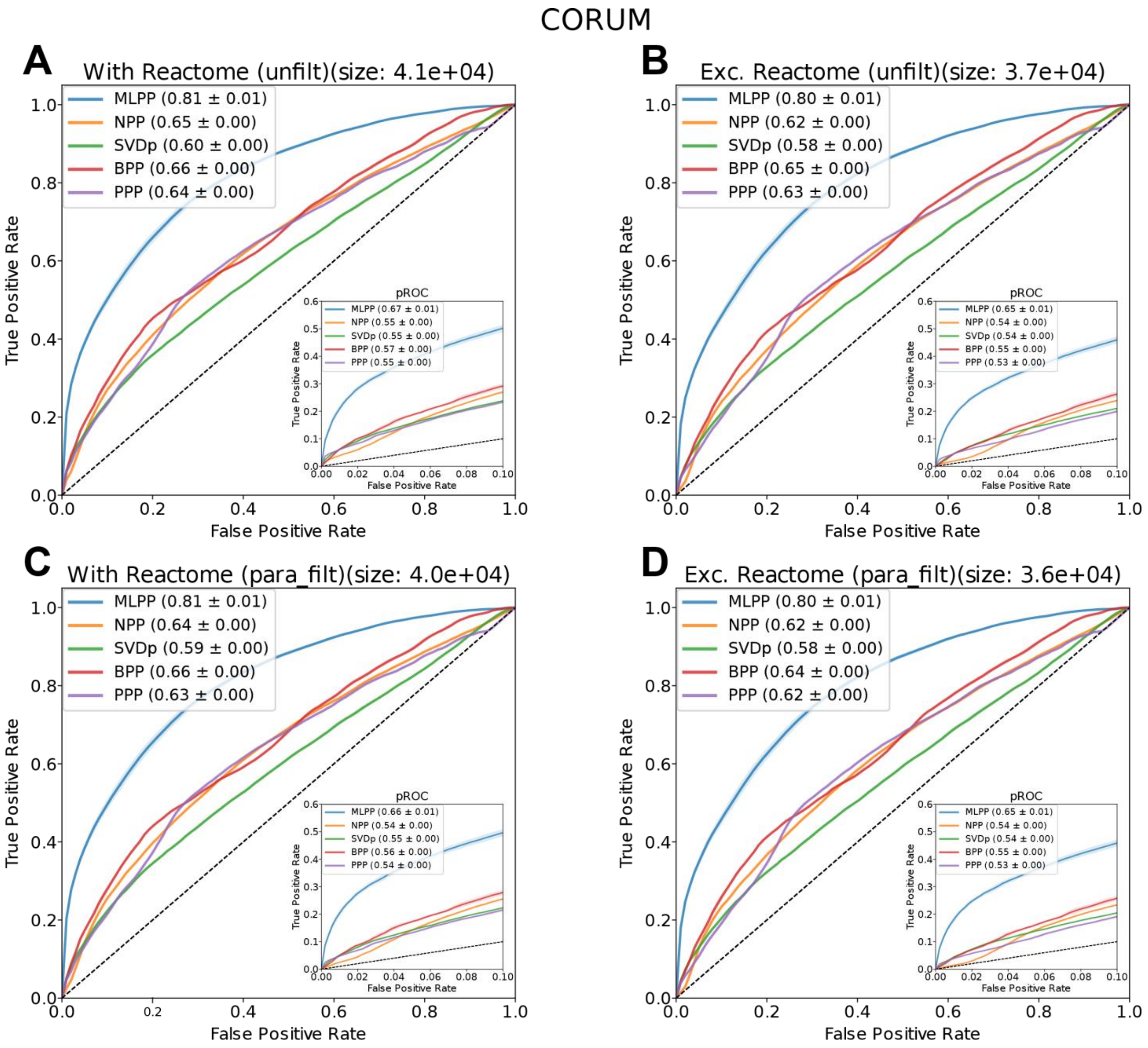

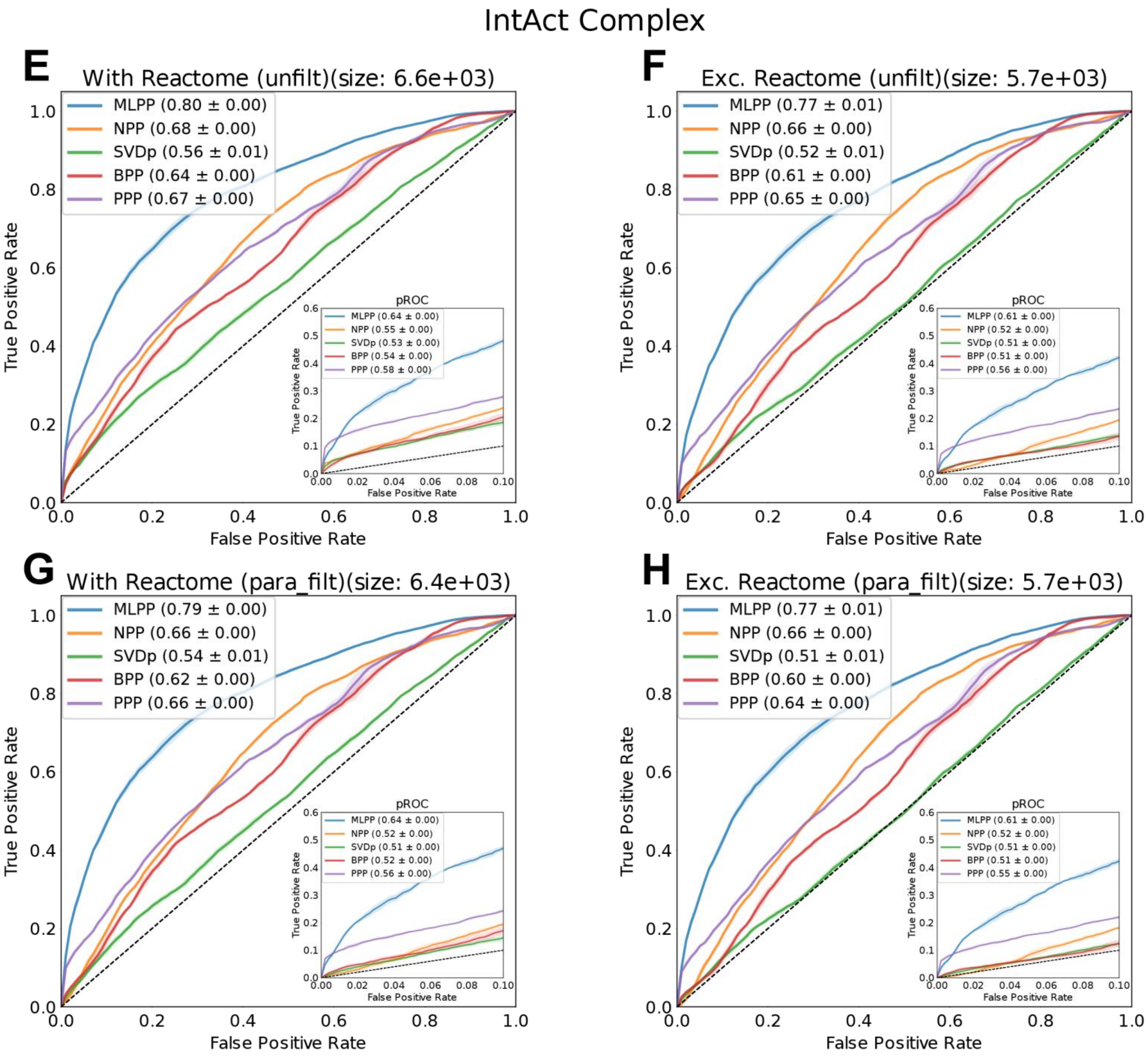

External Validation – Complexes (In Complex Model) - (A-H) Model performance in predicting complex co-occurrence in CORUM (A-D) or the IntAct Complex (E-H) databases. Performance was measured by a ROC curve and the AUC and using the In Complex model. In the inset of each panel is a pROC curve for the FPR range (0-0.1). Model evaluation was conducted with pairs of paralogs (A, B, E, F - “unfilt”) or without (C, D, G, H - “para_filt”) and with pairs that appear in Reactome, the database used for training (A, C, E, G) or without (B, D, F, H). Numbers in brackets are the AUC and the standard deviation of the AUC in cross-validations. ROC - receiver operator characteristics, AUC - area under the curve, pROC - partial ROC curve, FPR - false positive rate.

Supplementary Figure 11 - Performance Comparison on the Pathway Level

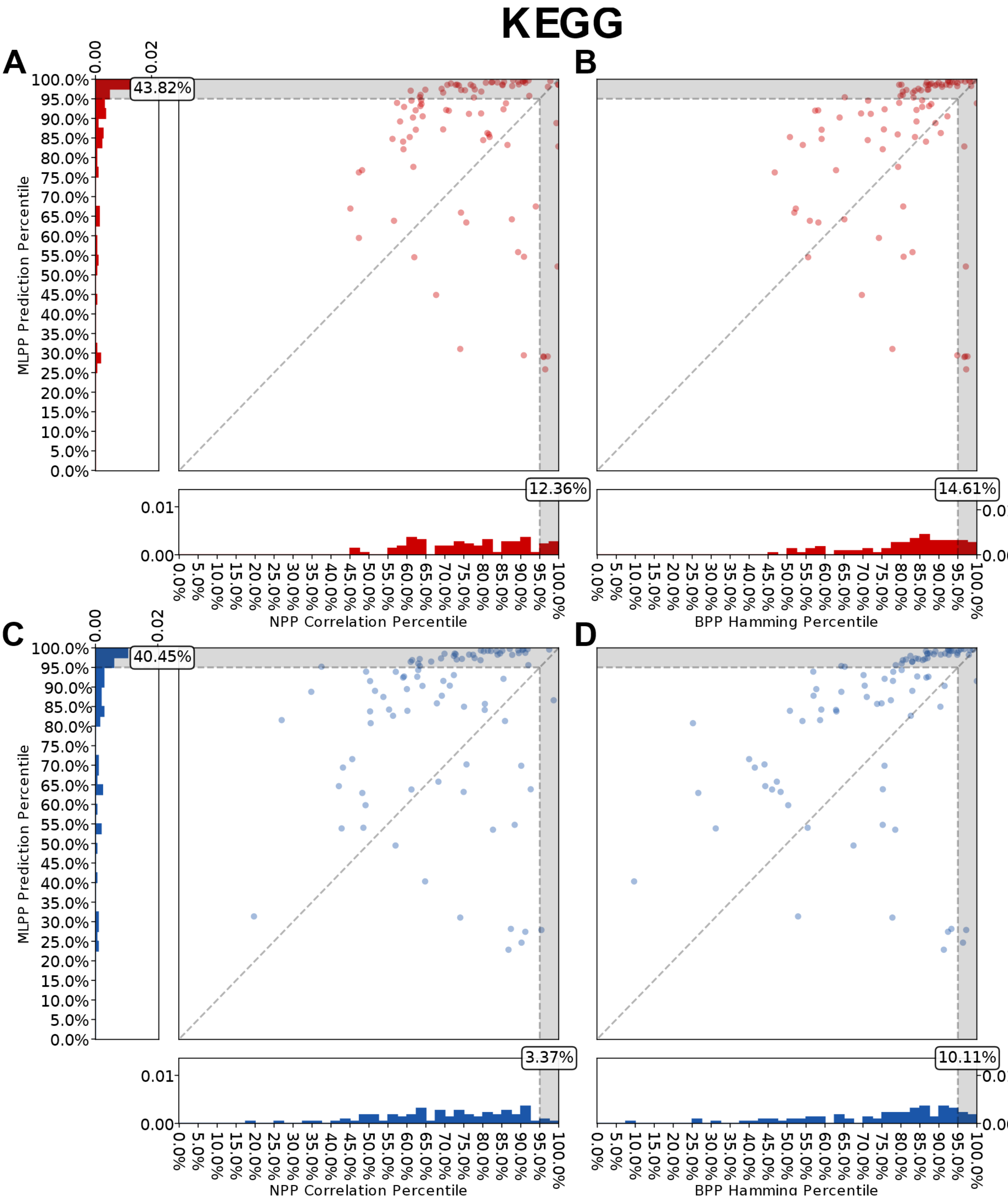

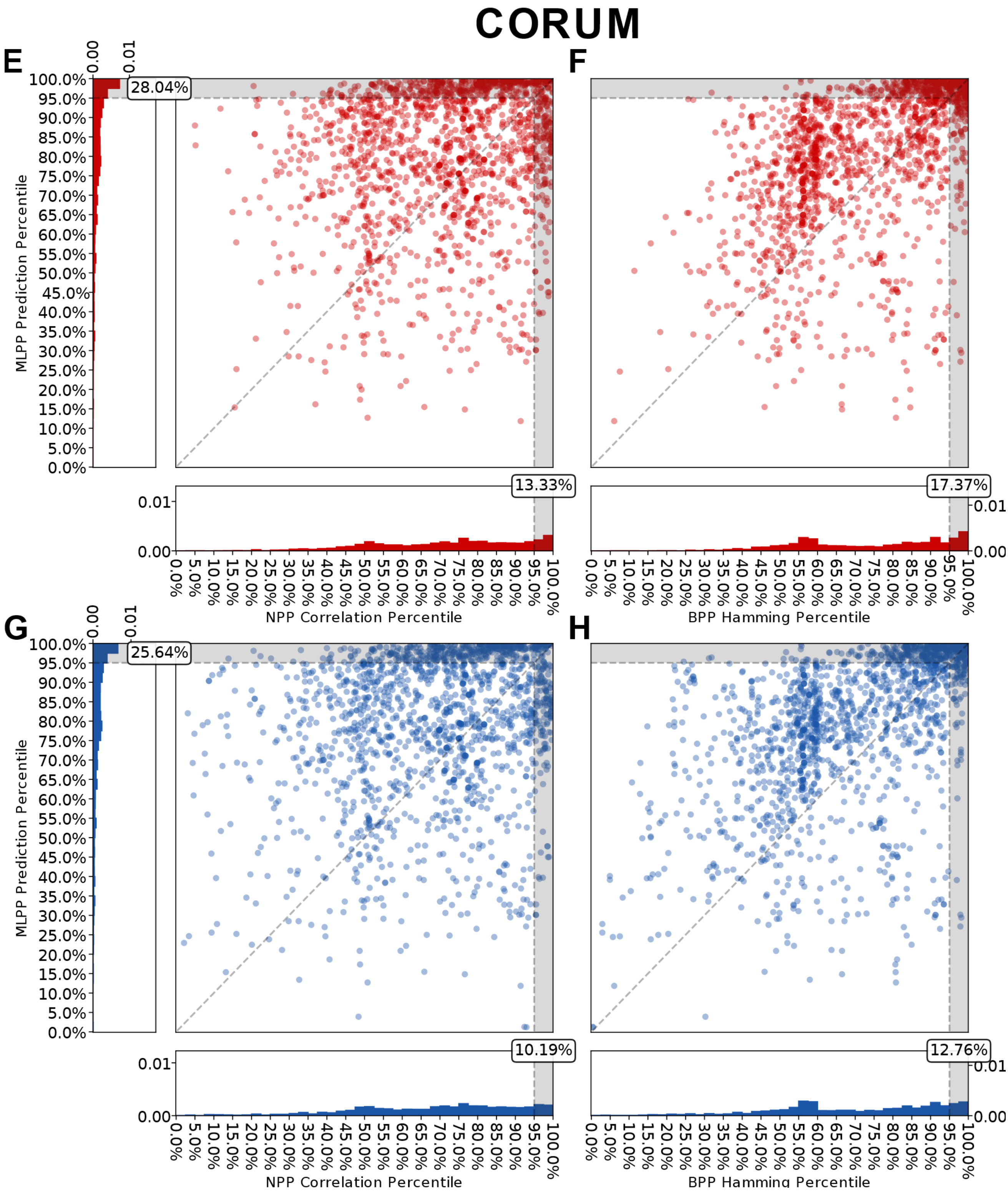

Performance Comparison on the Pathway Level - (A-H) Median score percentile for a gene set. Each dot is a single KEGG pathway (A-D) or CORUM complex (E-H) compared based on the score percentile in MLPP (y-axis) or in a different phylogenetic profiling approach (x-axis) - NPP (A, C, E, G) or BPP (B, D, F, H). Being above the diagonal dashed line indicates that the MLPP outperformed the other method for this gene set and vice versa. Gray percentile boxes show the percent of gene sets with a median score in the top 5% of scores for either MLPP (y-axis) or the compared approach (x-axis). Marginal histograms show the distribution of median score percentile of gene sets on the corresponding axis. Model evaluation was conducted with pairs of paralogs (A, B, E, F - in red) or without (C, D, G, H - in blue). MLPP - machine learning phylogenetic profiling (the method presented in this paper), NPP - normalized phylogenetic profiling, BPP - Hamming distance on binarized phylogenetic profiles. Source data are provided as a Source Data file.

Supplementary Figure 12 - Clusters

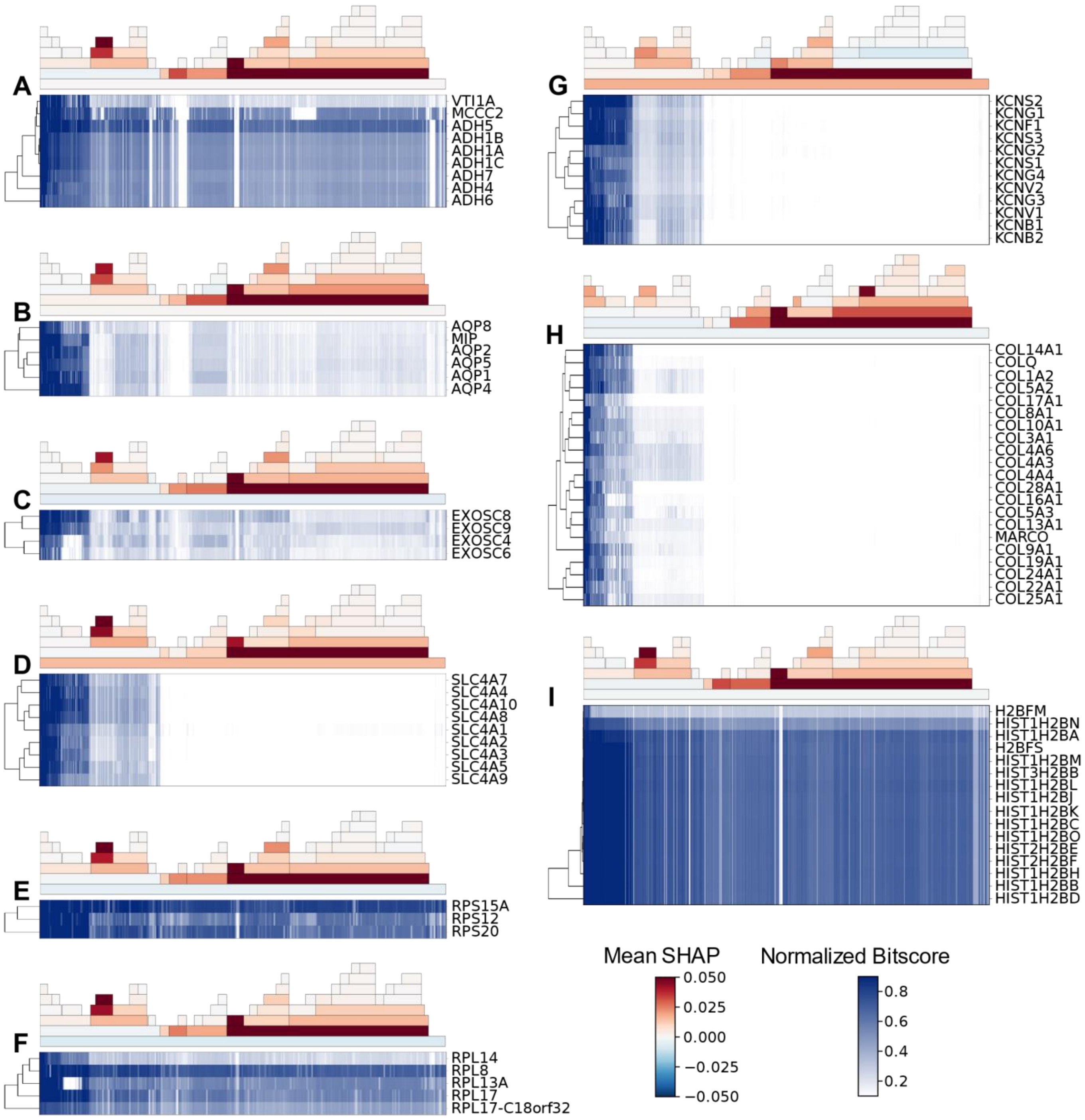

Specific Clusters - Model predictions for functional interaction were clustered using hierarchical clustering and cut at a specific heights corresponding to percentiles (top 0.05%, 0.1%, 0.5%, 1%, 2%, 2.5%) to produce clusters. For each of the clusters A-I, the top part is the clade importance for each clade as calculated using the mean SHAP value with species ordered from close (left) to distant (right) from human. The bottom part is the phylogenetic profile as self-hit normalized bitscores (1 - bitscore equal to self-hit, 0 - non-detected).

Supplementary Figure 13 - Distribution of Pubmed Mentions

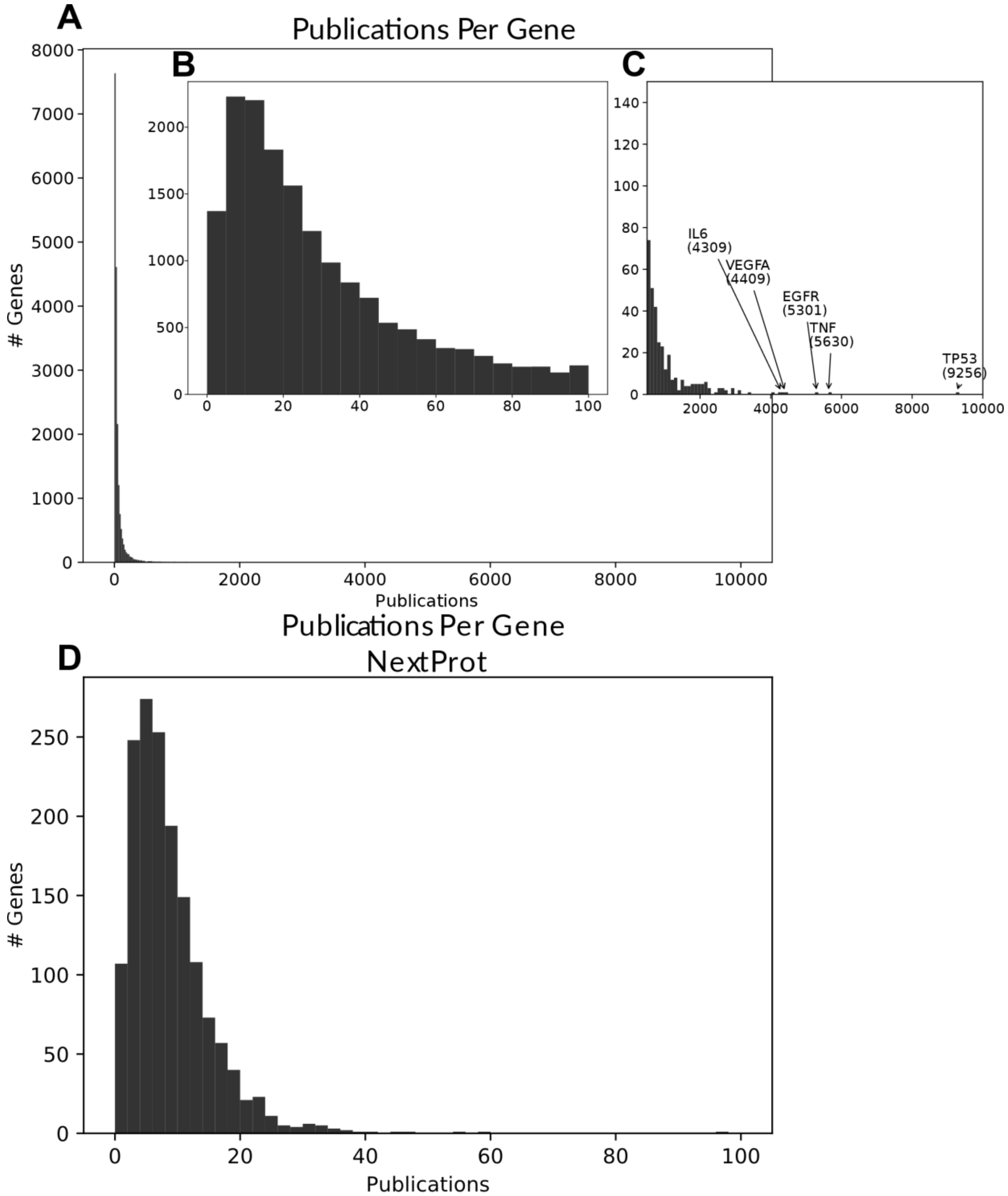

Distribution of Pubmed Mentions - (A-D) Histograms of Gene2Pubmed data mention counts per gene. (A) Distribution of Pubmed mentions for all genes. (B) Distribution of Pubmed mentions for genes with less than 100 mentions. (C) Distributions of Pubmed mentions for genes with more than 500 mentions. The top five most-mentioned genes are denoted by name with literature mention counts in brackets. (D) Distribution of Pubmed mentions for genes denoted as uncharacterized by NeXtProt (see Methods).

Supplementary Figure 14 – PathScore Distribution

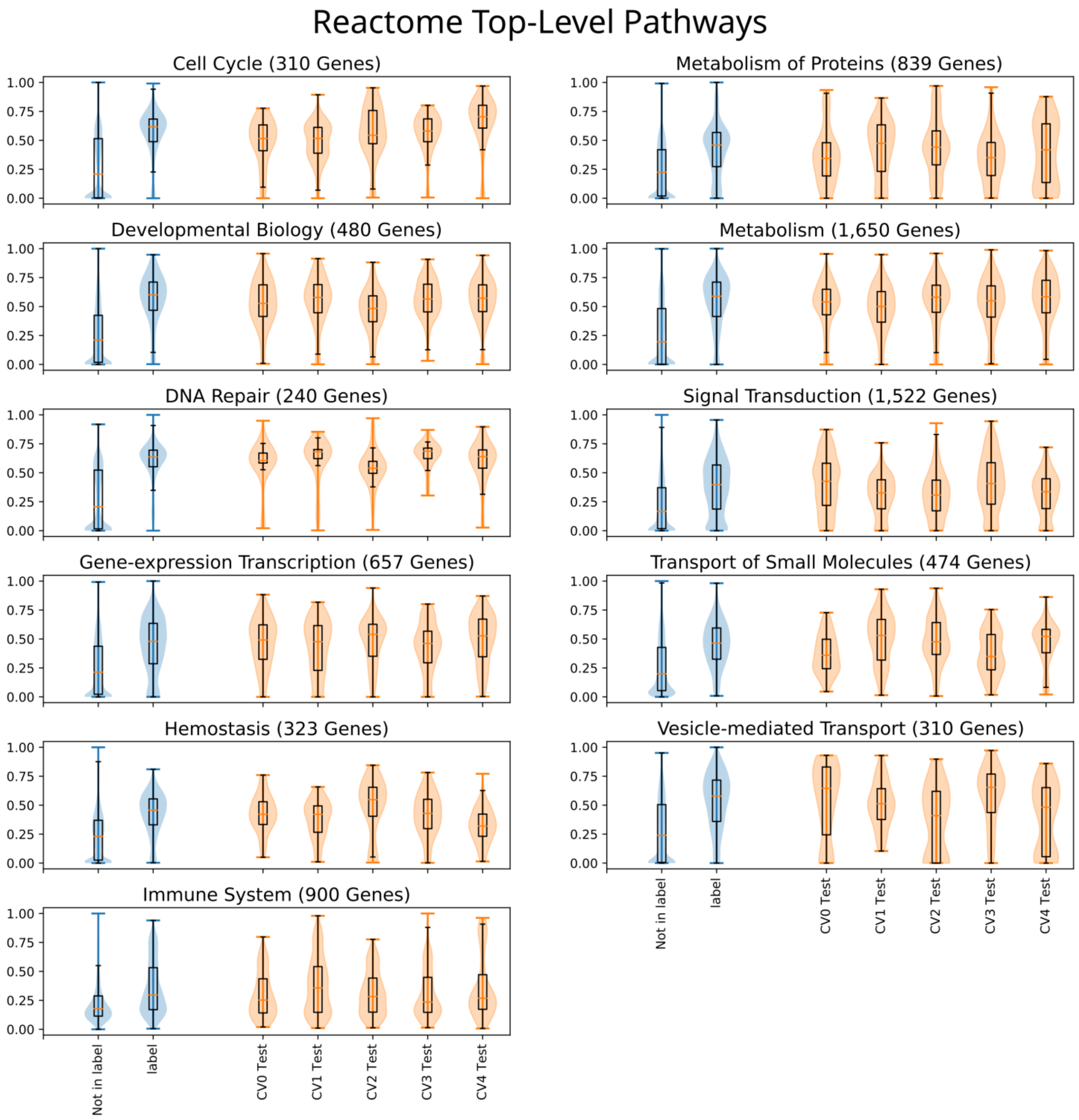

GO-BP

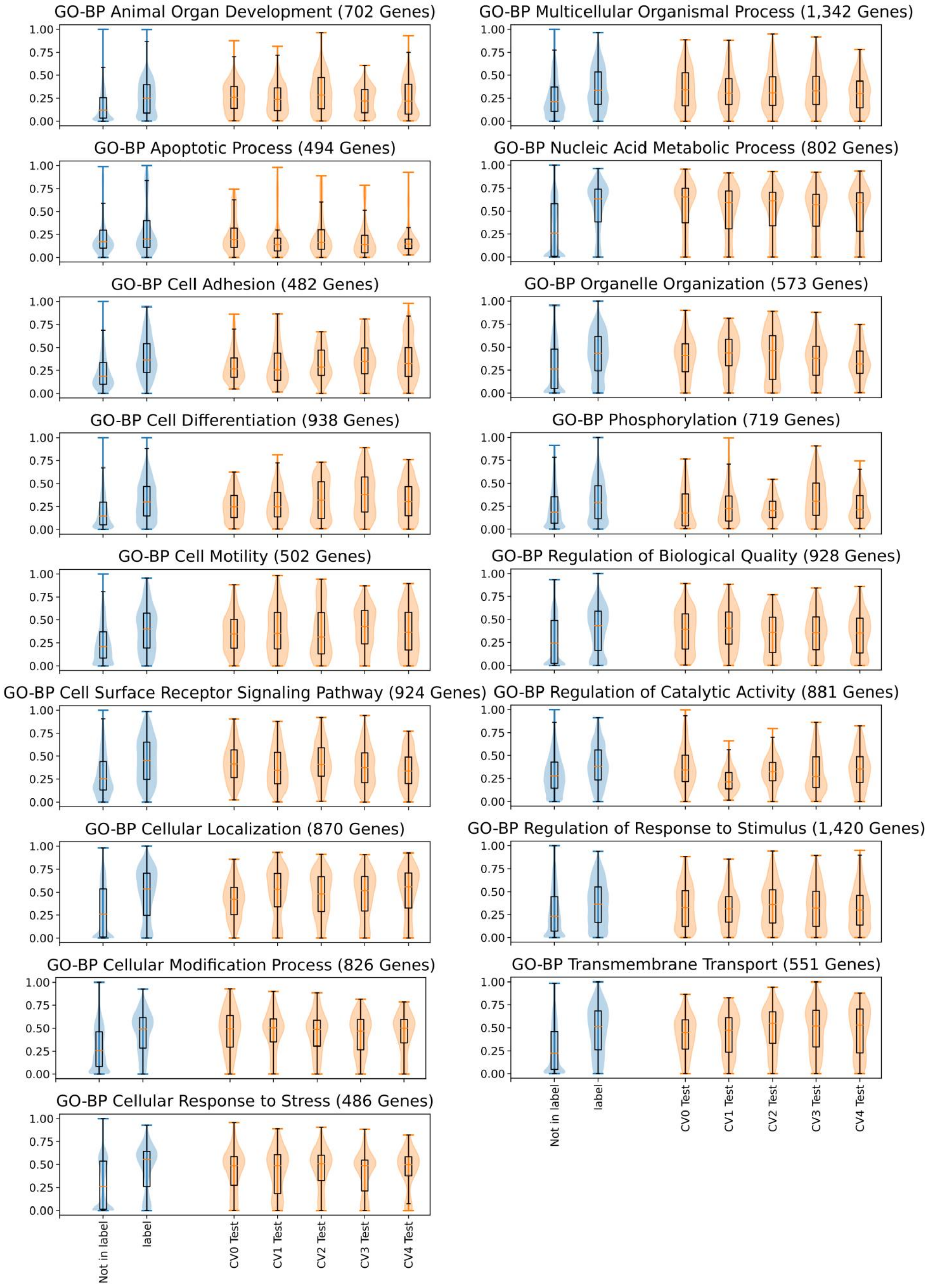
GO-BP Animal Organ Development (702 Genes)
GO-BP Multicellular Organismal Process (1,342 Genes)
GO-BP Apoptotic Process (494 Genes)
GO-BP Nucleic Acid Metabolic Process (802 Genes)
GO-BP Cell Adhesion (482 Genes)
GO-BP Organelle Organization (573 Genes)
GO-BP Cell Differentiation (938 Genes)
GO-BP Phosphorylation (719 Genes)
GO-BP Cell Motility (502 Genes)
GO-BP Regulation of Biological Quality (928 Genes)
GO-BP Cell Surface Receptor Signaling Pathway (924 Genes)
GO-BP Regulation of Catalytic Activity (881 Genes)
GO-BP Cellular Localization (870 Genes)
GO-BP Regulation of Response to Stimulus (1,420 Genes)
GO-BP Cellular Modification Process (826 Genes)
GO-BP Transmembrane Transport (551 Genes)
GO-BP Cellular Response to Stress (486 Genes)
1.00
0.75
0.50
0.25
0.00
Not in label
label
CV0 Test
CV1 Test
CV2 Test
CV3 Test
CV4 Test

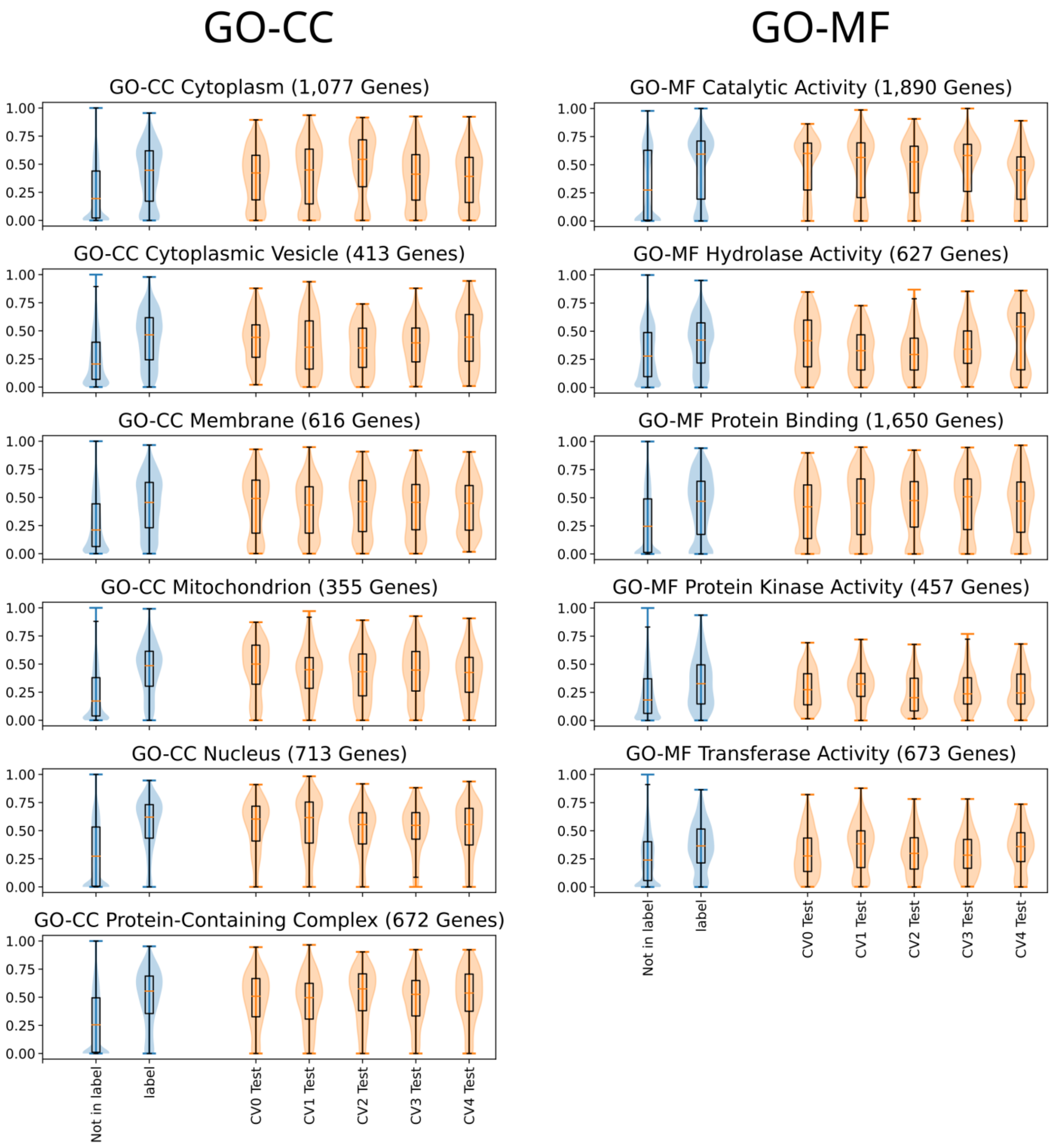

PathScore Distributions – PathScore distributions are presented as violin plots and overlying boxplot for each pathway type ("label", as described by the title of each panel). Violin plots are shown for genes annotated for this pathway-type ("label") and all other genes ("Not in label"). PathScore is also shown for genes in the pathway-type found exclusively in the test-set across five cross-validations ("CV0-CV4 Test"). Genes known to belong to each of the labels have higher PathScore scores then other genes, which is robust when these genes are found exclusively in the test set. The boxplot extends from the lower to upper quartile values of the data, with an orange line at the median. Whiskers denote 1.5 times the interquartile range.

Supplementary Figure 15 – PathScore Performance

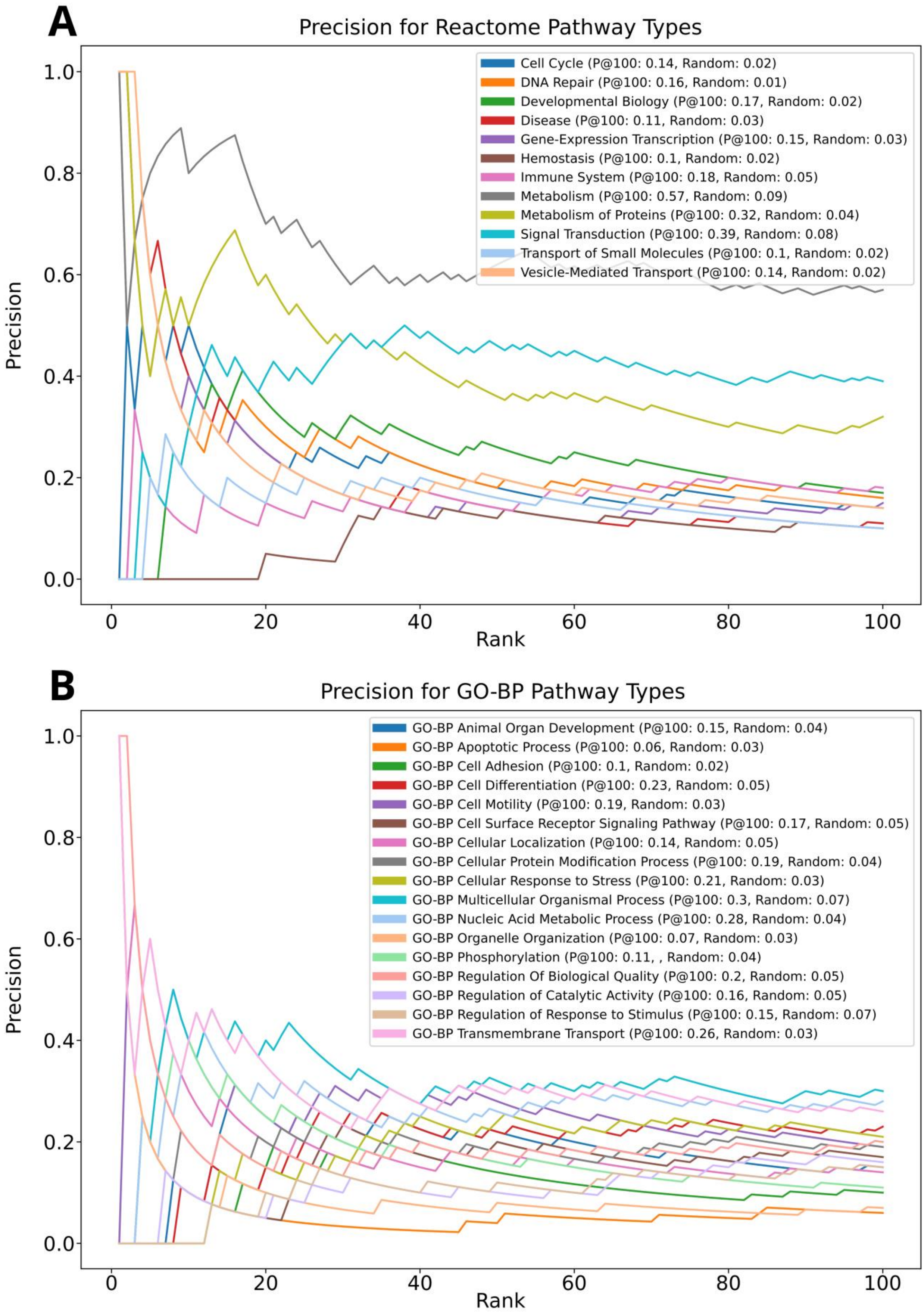

C

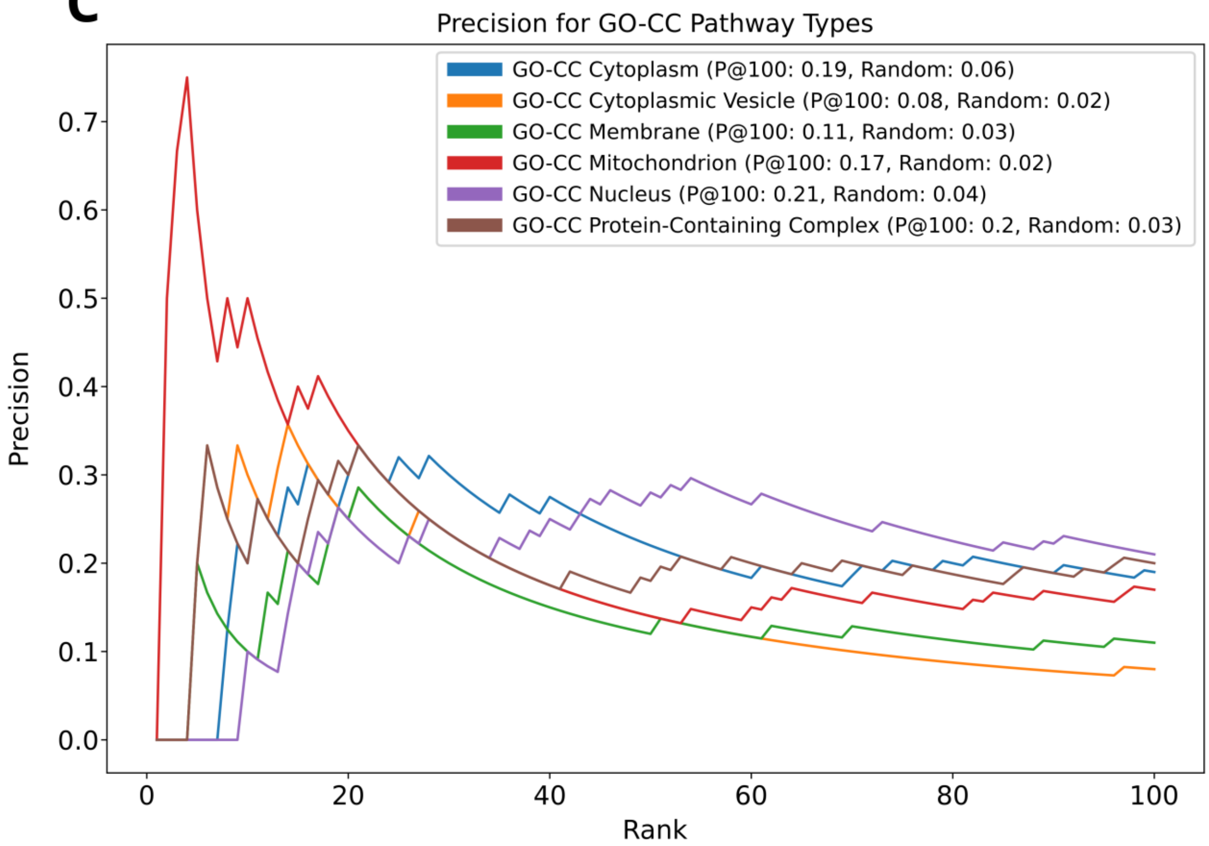

D

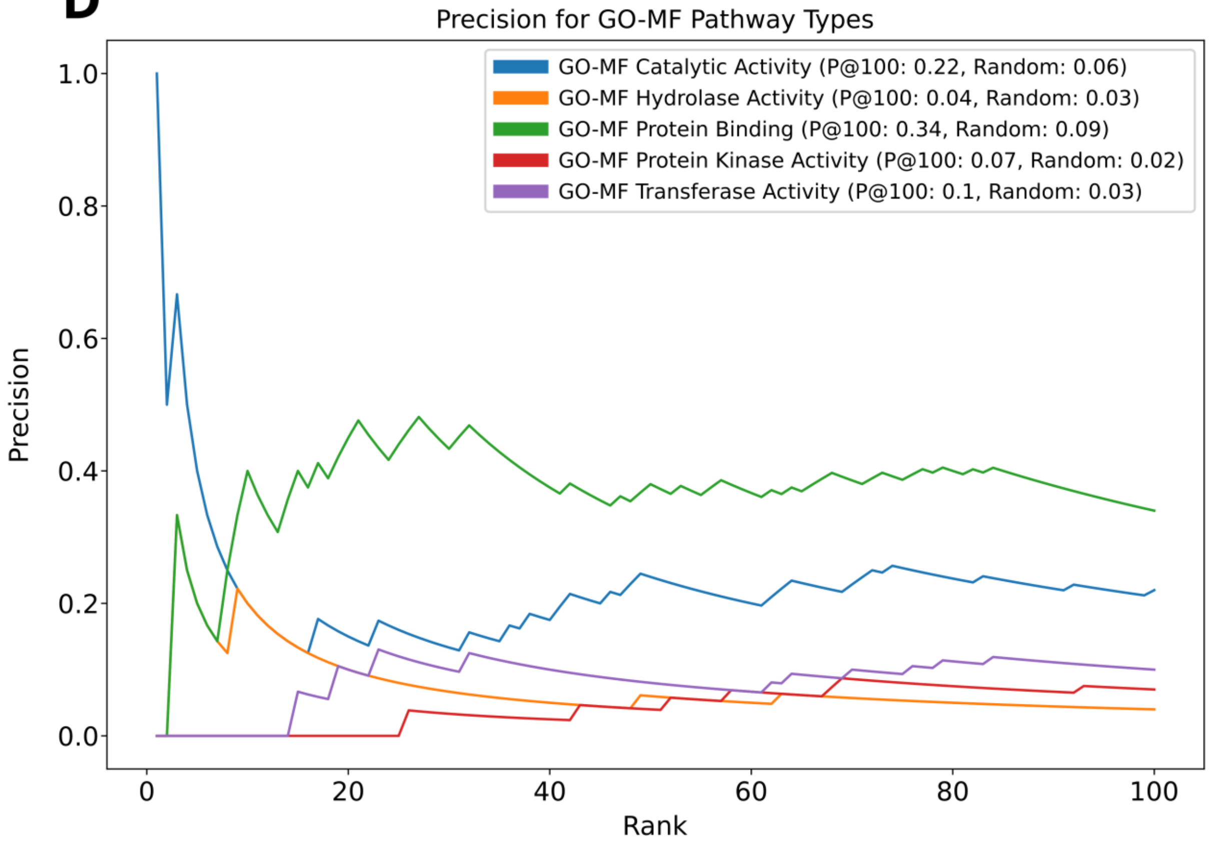

PathScore performance – Performance of PathScore functional annotations measured as precision at rank K for genes associated with the pathway-type. Pathway-types are grouped by source to Reactome (A), GO-BP (B), GO-CC (C), and GO -MF (D). Shown are top 100 ranks. Pathway types are denoted in the legend with the precision at rank 100 compared to random (frequency of genes associated with the pathway type). GO – gene ontology, BP – biological process, CC – cellular compartment, MF – molecular function. Source data are provided as Supplementary Data 3.

Supplementary Figure 16 - Clade Importance - Interaction Context Models

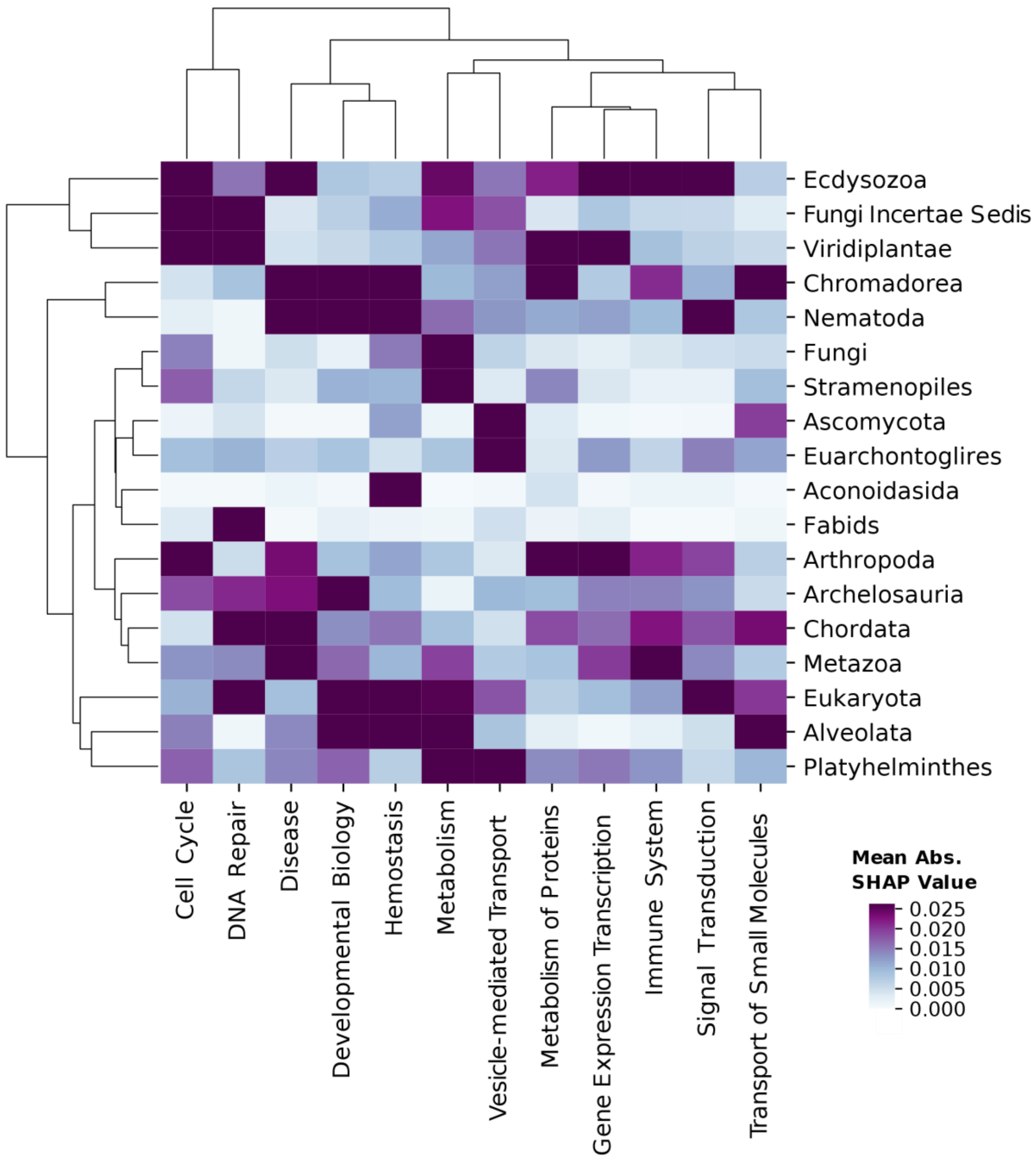

Heatmap of Clade Importance for Interaction Context Models - Clade importance calculated by SHAP values for the test set of a single cross-validation in each of the interaction context models, revealing the clades with the highest mean absolute importance. Shown are only clades with a maximal mean absolute SHAP value above 0.025.

Supplementary Figure 17 - UMAP Projection of SHAP Values for Gene Pairs

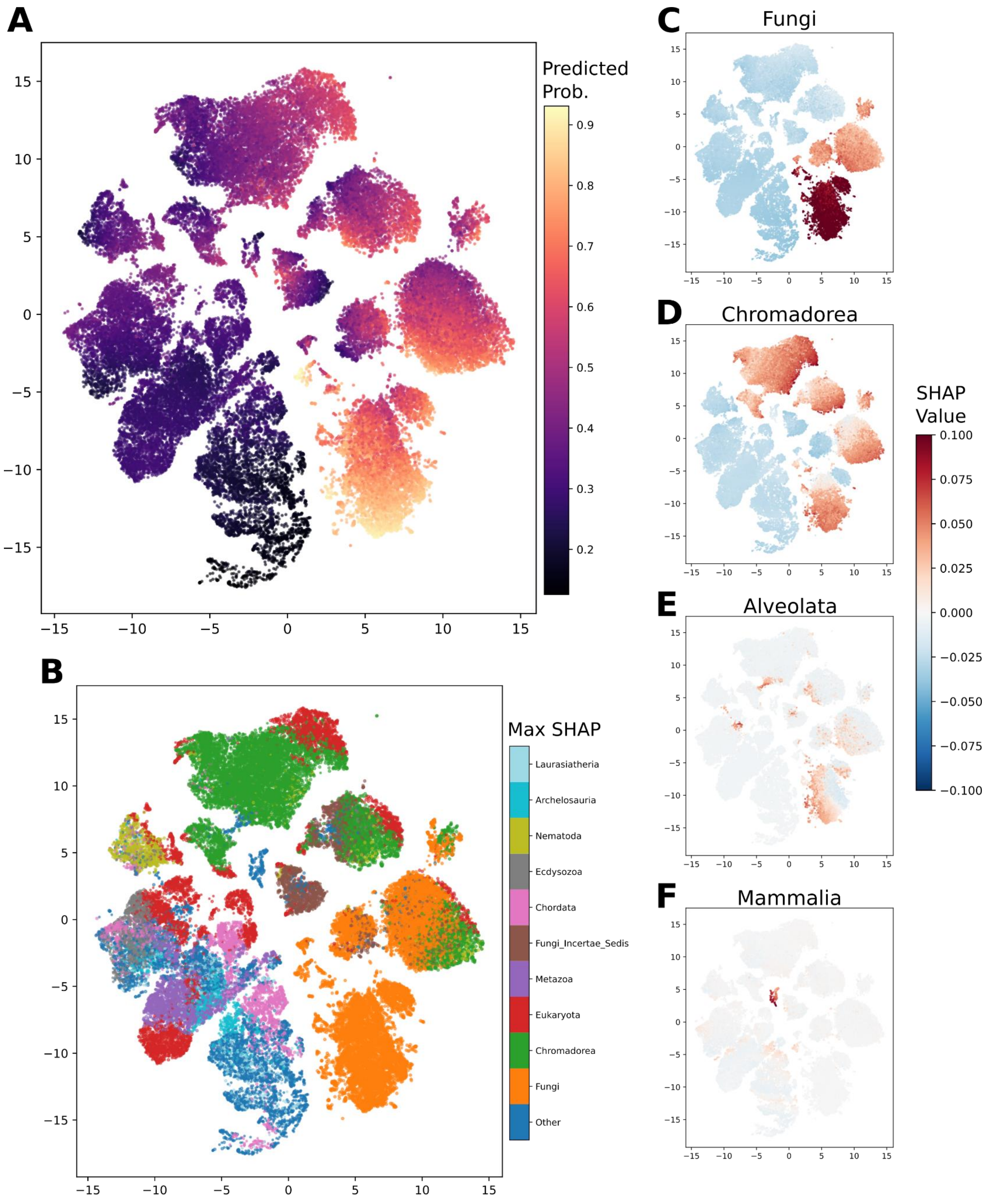

UMAP projection of SHAP values for gene pairs - (A) UMAP projection of SHAP values for gene-pairs in the test set of the Functional Interaction MLPP model (for the first cross-validation). Each dot is a gene-pair, colored by the total predicted probability for functional interaction for that pair. (B) Each gene-pair is colored by the clade with the highest SHAP value for this gene-pair. (C-F) Each gene-pair is colored by the SHAP value of a specific clade - Fungi (C), Chromadorea (D), Alveolata (E), Mammalia (F).

Supplementary Figure 18 – Clade Importance of Models Excluding Parasites

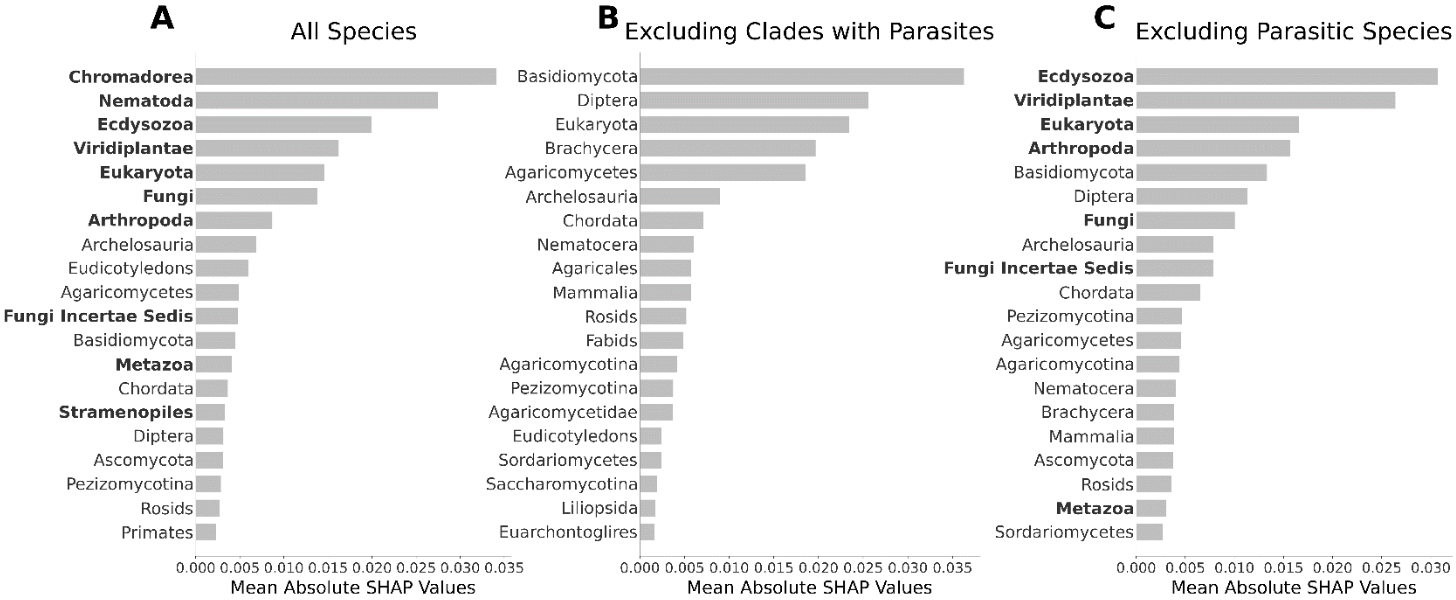

Clade Importance of Models Excluding Parasites – (A-C) Clade importance as mean absolute SHAP values of models trained to predict functional interactions in Reactome with all species (A), excluding parasitic species (B) and excluding all clades with any parasitic species (C). Clades in bold contain parasitic species.

Supplementary References

1. Park, Y. & Marcotte, E. M. Flaws in evaluation schemes for pair-input computational predictions. *Nature Methods* **9**, 1134–1136 (2012).
2. Duek, P., Gateau, A., Bairoch, A. & Lane, L. Exploring the Uncharacterized Human Proteome Using neXtProt. *Journal of Proteome Research* acs.jproteome.8b00537 (2018) doi:10.1021/acs.jproteome.8b00537.
