# Supplementary material for: Co-evolution based machine-learning for predicting functional interactions between human genes
Source: Nat Commun. 2021 Nov 9;12:6454. doi: 10.1038/s41467-021-26792-w (PMC8578642; doi:10.1038/s41467-021-26792-w)
Supplement: Supplementary file 2 — Description of Additional Supplementary Files [file 41467_2021_26792_MOESM2_ESM.pdf]

Description of Additional Supplementary Files

File Name: Supplementary Data 1
Description: **All Species** – taxonomic names and lineage of the 1154 species included in the phylogenetic profiles. Lineage is denoted as a semicolon-separated list of all parent clades. Species are ordered by distance from human.

File Name: Supplementary Data 2
Description: **Model Performance** – ROC AUC, pROC AUC (FPR 0.1), AP for all models presented (see Methods)

File Name: Supplementary Data 3
Description: **Ignorome –** PathScores and ranks for all genes for each of the pathway type models. Genes are annotated as Ignorome by neXtProt 2 or by being at the bottom quantile of PubMed citations (see Methods). Genes are ordered by the number of pathway types in which they are found in the top 250 genes. Known denotes if a gene in found in any Reactome pathway in the specific pathway type.
